# A Facile Synthesis of 3‐Substituted Coumarins and Investigation of Their 3CLpro Inhibition Activity Against SARS‐CoV‐2

**DOI:** 10.1002/open.202400319

**Published:** 2024-11-26

**Authors:** Manoj K. Choudhary, Khalid Ansari, Vivek Junghare, Sandip K. Nayak, Saugata Hazra, Soumyaditya Mula

**Affiliations:** ^1^ Bio-Organic Division Bhabha Atomic Research Centre Mumbai 400085 India; ^2^ Homi Bhabha National Institute, Anushakti Nagar Mumbai 400094 India; ^3^ Department of Physics Indian Institute of Technology Roorkee 247667 India; ^4^ Department of Biosciences and Bioengineering Centre for Nanotechnology Indian Institute of Technology Roorkee 247667 India

**Keywords:** Coumarins, Morita-Baylis-Hillman adducts, Friedel-Crafts alkylation, 3CLpro Inhibitors, SARS-CoV-2, Molecular Dynamics simulation, ADMET study

## Abstract

The major threat to public health due to the outbreak of severe acute respiratory syndrome coronavirus‐2 (SARS‐CoV‐2) infection has been recognised as a global issue. The increase in morbidity is primarily due to the lack of SARS‐CoV‐2 specific drugs. One of the major strategies to combat this threat is to deactivate the enzymes responsible for the replication of corona virus. To this end, 3‐arylidene/3‐hydroxycoumarin induced deactivation of 3‐chymotrypsin like protease (3CLpro) enzyme, which takes the pivotal role in the replication and maturation, was investigated. For ready availability of the compounds for the above investigation, we have developed a user‐friendly protocol for the synthesis 3‐hydroxycoumarin derivatives from cheap and readily available starting materials in two steps; i) Bronsted acid catalysed Friedel‐Crafts alkylation of phenols with Morita‐Baylis‐Hillman adducts followed by intramolecular lactonization to trans‐3‐arylidenechroman‐2‐ones in one‐pot and ii) ozonolysis in reasonably good yields. Pharmacokinetic assessments of coumarin derivatives revealed drug‐like characteristics with moderate or low toxicity values. Notably, these hydroxycoumarins exhibited enhanced binding affinity against the 3CL protease of SARS‐CoV‐2, fitting well into the binding pocket akin to the previously studied inhibitor N3. Furthermore, a molecular dynamics study elucidated the dynamic behaviour of these small molecules when bound to the protein, showcasing intriguing complexities within the active site. Despite backbone variations and residual fluctuations, compounds **3 d–f** and **6 a** exhibited a consistent behaviour, instilling confidence in the therapeutic potential of these coumarins for combating SARS‐CoV‐2.

## Graphical abstract

A user‐friendly protocol for the synthesis 3‐substituted coumarin derivatives was developed using Friedel‐Crafts alkylation and ozonolysis as key steps. Deactivation of 3‐chymotrypsin like protease (3CLpro) enzyme using these coumarin derivatives were investigated to explored their potential as 3CLpro inhibitors for Covid‐19 Therapeutics.

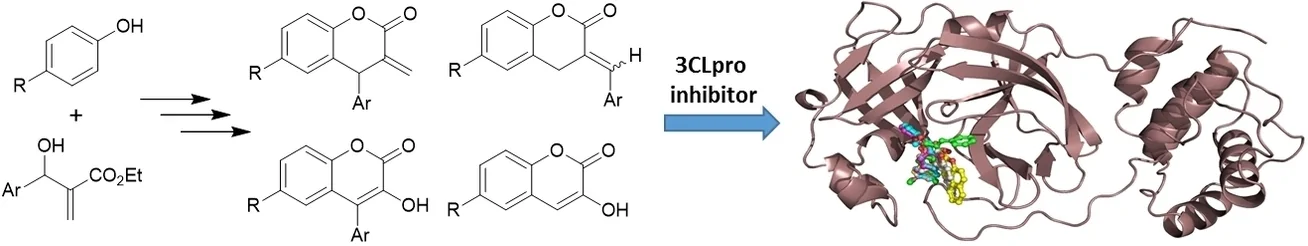

## Introduction

The emerging pandemic outbreak in the present century due to severe acute respiratory syndrome coronavirus‐2 (SARS‐CoV‐2) has become a global threat to public health because of its high rate of infection leading to mortality. The primary cause of morbidity is due to lack of SARS‐CoV‐2 specific drugs or vaccines. The 3‐chymotrypsin like protease (3CLpro) plays a pivotal role in the replication and maturation of coronaviruses, including the severe acute respiratory syndrome coronavirus‐2 (SARS‐CoV‐2) responsible for the COVID‐19 pandemic.^[1]^ To address this issue, development of potential drugs that target the 3‐chymotrypsin like protease (3CLpro), was chossen as main strategy for antiviral drug development. Although synthetic pharmaceuticals have traditionally been explored as 3CLpro inhibitors, there is a growing interest in using naturally derived small organic molecules for their potential inhibitory effects. Natural compounds isolated from plants, herbs, and various origins boast a rich history of medicinal use and present a diverse array of bioactive molecules capable of interacting with the 3CLpro enzyme. Numerous studies have underscored the potential of naturally derived drugs in inhibiting 3CLpro activity and disrupting viral replication. For instance, polyphenols such as quercetin, epigallocatechin gallate (EGCG), and curcumin have exhibited inhibitory effects on 3CLpro activity in vitro.^2^, ^3^ These compounds, present in various plant sources, possess antioxidant and anti‐inflammatory properties that contribute to their potential antiviral activity.

Dietary polyphenols, isolated from plants/plant‐derived foods, are known to display several biological properties, including anti‐oxidative and anti‐inflammatory activities. Naturally occurring flavonoids, have also shown inhibitory effects against 3CLpro. Recent study have shown that the polyhydroxy flavones such as baicalin, luteolin, and apigenin can effectively inhibit the proteolytic activity of the enzyme through binding to the enzyme's active site.^4^, ^5^ Also, medicinal plant derived alkaloids, such as berberine and tetrahydroprotoberberine derivatives, have shown great promise as 3CLpro inhibitors, exhibiting antiviral activity that disrupts viral replication by targeting the enzymatic activity of 3CLpro.^[6]^ Additionally, terpenoids, including essential oils derived from plants, have displayed inhibitory effects against 3CLpro. Compounds such as thymol, carvacrol, and eugenol have been shown to interfere with the enzyme's activity, potentially hindering viral replication.^[7]^

The in‐depth study on the mechanism of interactions of naturally derived drugs with the 3CLpro enzyme remains a challenging task in the development of antiviral therapies. Researchers are now aiming to understand the structural characteristics of 3CLpro and the specific mechanisms by which these compounds engage with the enzyme. This pursuit seeks to develop effective inhibitors capable of disrupting viral replication, thereby mitigating the impact of coronavirus infections. In essence, the investigation on naturally derived drugs as 3CLpro inhibitors provides a hopeful avenue for antiviral therapy development against coronaviruses. The rich array of bioactive compounds found in nature serves as a valuable resource for discovering and optimizing potent inhibitors. Continuous research and optimization of these compounds present substantial potential in the ongoing battle against viral infections.

Natural products are consistently used as lead material in drug development programme due to their biological compatibility.^[8]^ Several natural products show promise as candidates for developing 3CLpro inhibitors of SARS‐CoV‐2.^[9]^ Previously, we have also shown that dihydrobenzofuro[3,2‐b]chromenes exhibit favorable binding interactions with 3CLpro.^[10]^ Among the naturally occurring oxygen heterocycles, coumarin and its derivatives are crucial core units in various bioactive natural products and pharmaceutical compounds, as well as essential building blocks in organic and medicinal chemistry.^[11]^ They demonstrate diverse bioactivities, including antioxidant, anti‐cancer, anti‐inflammatory, cardioprotective, and MAO inhibitory activity.^12^, ^13^ While their activities against 3CLpro have been studied, there is limited information on the structure‐activity relationship concerning their inhibitory activity.

Recent advances in synthetic methodologies that allow rapid access to a wide variety of functionalized heterocyclic compounds are of critical importance to the medicinal chemist as it provides the ability to carry out in depth study in drug discovery programs. Furthermore, the development of efficient synthetic protocols to generate bulk quantities of a desired heterocyclic compounds will certainly help to accelerate the drug development process.

A large number of coumarin derivatives including 4‐hydroxycoumarins are known to possess various important biological activities. Hydroxycoumarins, particularly 4‐ and 7‐hydroxycoumarins, have been extensively studied for their diverse bioactivities, such as anticoagulant, antioxidant, cytostatic, antibacterial, antiviral, xanthine oxidase inhibition, antihypoglycemic, and casein kinase 2 inhibitory activity with low side‐effects and minor toxicity.^[14]^ Many of them also act as metal chelators and free radical scavengers.^[15]^ Although the 3‐hydroxycoumarin scaffold is present in medicinally active natural products,^[16]^ there are limited reports on their synthesis.^[17]^ This has become a major hurdle on the investigation of their biological activities in drug discovery program.^[16]^

Accordingly, a facile protocol has been developed for the synthesis of 3‐hydroxycoumarins using cheap and readily available starting materials. To this end, (±)‐camphor‐10‐sulfonic acid catalyzed Friedel‐Crafts alkylation‐lactonization of 4‐substituted phenols with Morita‐Baylis‐Hillman (MBH) adducts (derived from aromatic aldehydes and methyl acrylate) proceeded in one‐pot under solvent‐free conditions to afford 3‐methylene/3‐arylidenechroman‐2‐ones (S_N_2’ product) in good yields. The present method offers several advantages such as low catalyst loading and a solvent‐free green approach, a distinct advantage over existing synthetic methods. The synthesized 3‐methylene/3‐arylidenechroman‐2‐ones in turn converted into corresponding 3‐hydroxycoumarins in good yield via ozonolysis.

In‐silico structure construction of small molecules was performed using ChemDraw Ultra in 2D, followed by energy minimization using the built‐in tool of the Chem3D tool to obtain three‐dimensional structures. The SARS‐CoV‐2 3CLpro enzyme was obtained from RCSB using PDB ID 6LU7. Molecular docking with Autodock Vina software was conducted to study the interactions of fifteen synthesized compounds with 3CLpro. Molecular dynamics simulations were performed for selected compounds for a total of 100 ns each (total computational time700 ns duration). Analysis techniques, including root mean square deviation (RMSD), root mean square fluctuation (RMSF), hydrogen bonds, radius of gyration, and solvent accessible surface area (sasa), were employed to study conformational and structural stability. A brief analysis of the compounds’ compatibility with the protein was also conducted. Lastly, in‐silico ADMET screening of the fused flavonoids examined various physicochemical characteristics such as cell permeability, lipophilicity, water solubility, and various pharmacokinetics.

## Results and Discussion

### Synthesis

Morita‐Baylis–Hillman (MBH) reaction is now considered as a standard synthetic method for the preparation of a new class of densly‐functionalized allylic alcohol *via* the reaction of electron‐deficient alkene with an aldehyde in the presence of a tertiary amine as catalyst.^[18a,b]^ Thus, MBH reaction using aromatic aldehyde and methyl acylate afforded a highly reactive alcohol (both an allylic as well as benzylic alcohol). MBH adducts are being recognized as one of the most important precursor in organic synthesis as it possess three important functional groups *viz*. a hydroxyl group, a double bond and an electron withdrawing group.^[18c–e]^ The potential of MBH adduct has been demonstrated in the synthesis of wide range of heterocycles including chromenes, chromones, furanones, furo‐pyrans, spiro‐γ‐butyrolactones, spiro‐isoxazolines, piperdines, pyrazolines, indoloazocines, quinolones, indolones, γ‐lactams.^18^, ^19^ Previously, Liu *et al*. reported the synthesis of 3‐arylidenechroman‐2‐one scaffold by temperature controlled gold catalyzed [1,3]‐sigmatropic rearrangement of 2‐(aryloxymethyl)‐alk‐2‐enotes.^[20a]^ Similarly, Gopal *et al*. used Claisen rearrangement of methyl 3‐aryloxy‐2‐(aryloxymethyl)prop‐2‐enoates for the synthesis of 3‐arylidenechroman‐2‐one.^[20b]^

**Scheme 1:**
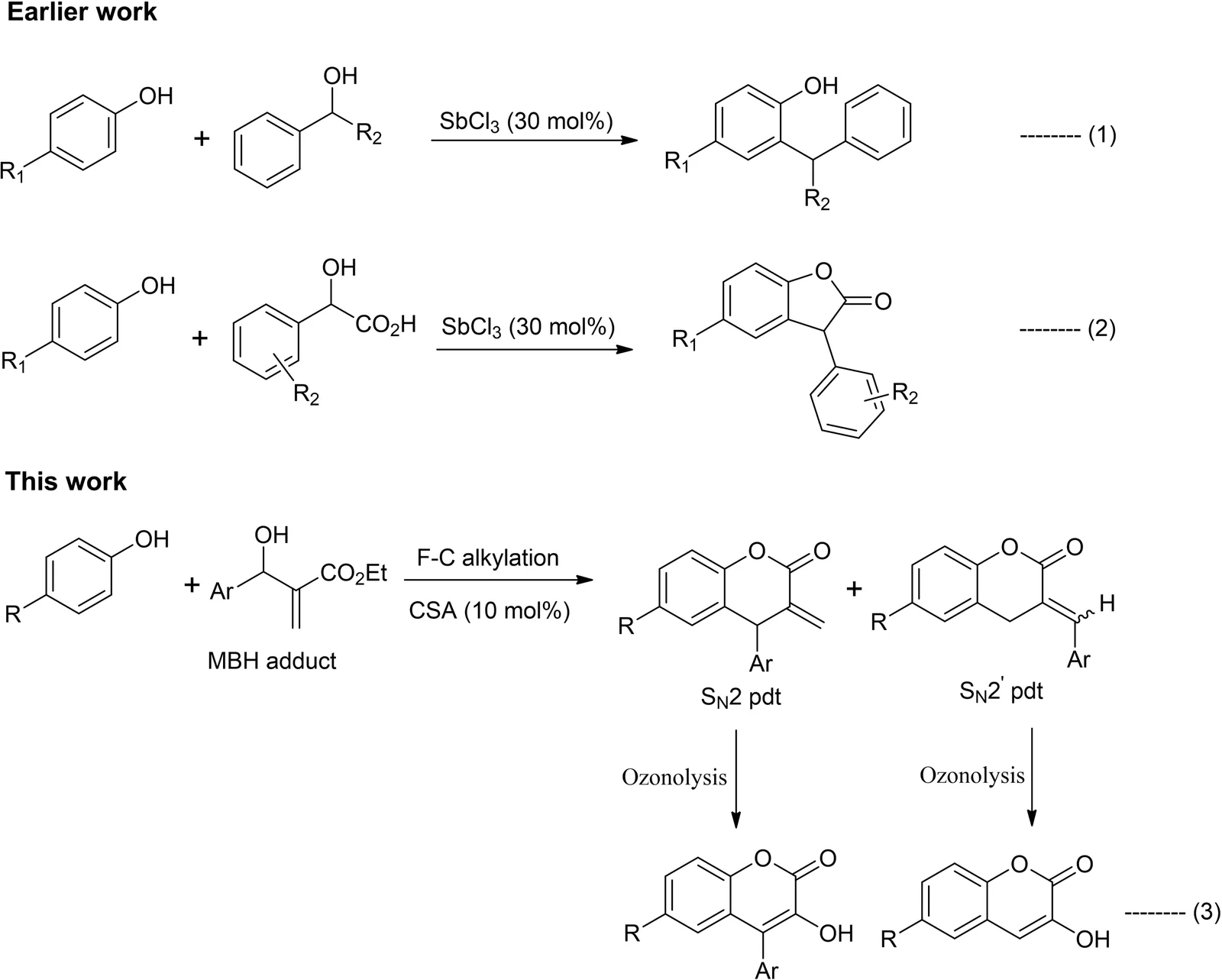
Friedel‐Crafts alkylation reaction of phenols for synthesis of 3‐substituted coumarins.

Previously, our group demonstrated an antimony(III) chloride (SbCl_3_) catalysed facile Friedel‐Crafts (F−C) alkylation of phenols and aryl alkyl ethers with benzylic/allylic alcohols (Scheme 1; eqn 1).^[21a]^ In another work we demonstrated that 3‐aryl benzofuran‐2(3*H*)‐ones skeletone (a five membered lactone) skeleton was accomplished through F−C alkylation followed by lactonization of phenols with mandelic acids (Scheme 1; eqn 2).^[21b]^

By retrosynthetic analysis, we felt that Friedel‐Crafts alkylation of 4‐substituted phenols with MBH adducts can in principle proceed following both S_N_2 and S_N_2’ pathway to afford corresponding hydroxy esters which in turn can undergo intramolecular lactonization to afford 4‐aryl‐3‐methylenechroman‐2‐one (S_N_2 product) and/or 3‐arylidenechroman‐2‐one (S_N_2’ product) scaffolds. Finally, ozonolysis of 3‐arylidene chroman‐2‐ones will afford the corresponding 3‐hydroxy coumarin derivatives (Scheme 1, eqn. 3).

To this end, a preliminary F−C reaction of 4‐methylphenol (**1 a**) with ethyl 2‐[hydroxy(phenyl)methyl]acrylate (**2 a**) in presence of SbCl_3_ (5 mol %) catalyst in acetonitrile was carried under refluxing conditions (90 °C) when no reaction was observed (Table 1; entry 1). Increase in catalyst loading to 10 mol % also could not initiate the reaction (Table 1; entry 2). Earlier, we found that SbCl_3_ catalyzed F−C alkylation reaction of phenols proceeds well under solvent free conditions.^[21b]^ Keeping this in view, the above reaction was carried out without solvent at 100 °C but without any success (Table 1; entry 3). Increasing the reaction temperature to 120 °C failed to initiate the reaction (Table 1; entry 4). However, to our delight when the reaction was carried out at 140 °C, regio‐selective reaction took place *via* a S_N_2’ pathway to afford (*E*)‐3‐benzylidene‐6‐methyl‐chroman‐2‐one (**3 a**) as the sole product in 44 % yield (Table 1; entry 5). The other possible regio‐isomeric product (*via* S_N_2 pathway), 6‐methyl‐3‐methylene‐4‐phenylchroman‐2‐one (**4 a**) was not detected.

**Table 1:** Optimization of reaction conditions for CSA‐catalyzed Friedel‐Crafts alkylation/lactonization of **1 a** with **2 a** to *trans*‐6‐methyl‐3‐benzylidenechroman‐2‐one (**3 a**).

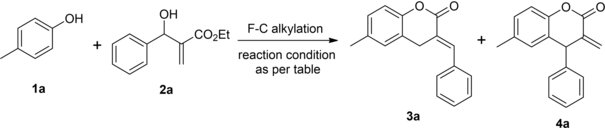						
Entry	Catalyst	Catalyst loading (mol %)	Solvent	Temp (°C)/time (h)	% yield of **3 a**	% yield of **4 a**
1	SbCl_3_	5	CH_3_CN	90/6	no rxn	‐
2	SbCl_3_	10	CH_3_CN	90/6	no rxn	‐
3	SbCl_3_	5	‐	100/4	no rxn	‐
4	SbCl_3_	5	‐	120/4	no rxn	‐
5	SbCl_3_	5	‐	140/6	44	‐
6	SbCl_3_	10	‐	140/4	50	‐
7	SbCl_3_	15	‐	140/4	50	‐
8	InCl_3_	5	‐	100/4	41	‐
9	InCl_3_	10	‐	100/4	42	‐
10	Yb(OTf)_3_	2	‐	100/4	50	‐
11	CSA	5	‐	100/4	68	‐
12	CSA	10	‐	100/2	80	‐

With increased catalyst loading (10 mol %), the yield of **3 a** improved marginally (50 %) (Table 1; entry 6) and further increase in catalyst loading (15 mol %) did not improve the yield of **3 a** further (Table 1; entry 7). To optimize the yield of **3 a**, F−C reaction of **1 a** with **2 a** was screened with other Lewis/Brønsted acid catalysts such as InCl_3_, Yb(OTf)_3_ and camphor‐10‐sulfonic acid (CSA) (Table 1; entries 8–12). Among these CSA was found to be superior in terms of yield (80 %) and reaction time (Table 1; entries 12). Importantly, Foucaud *et al*. reported the F−C alkylation of phenols with MBH adduct, but the amount of Lewis acid (silica gel supported/ZnBr_2_) used was high (0.5 g/mmol of phenol) and reaction time was longer (6 h) under refluxing conditions using 1,2‐dichloroethane as solvent.^[22]^ Thus, the present methodology is superior in terms of low catalyst loading, lower reaction time and most importantly solvent free approach.

**Scheme 2:**
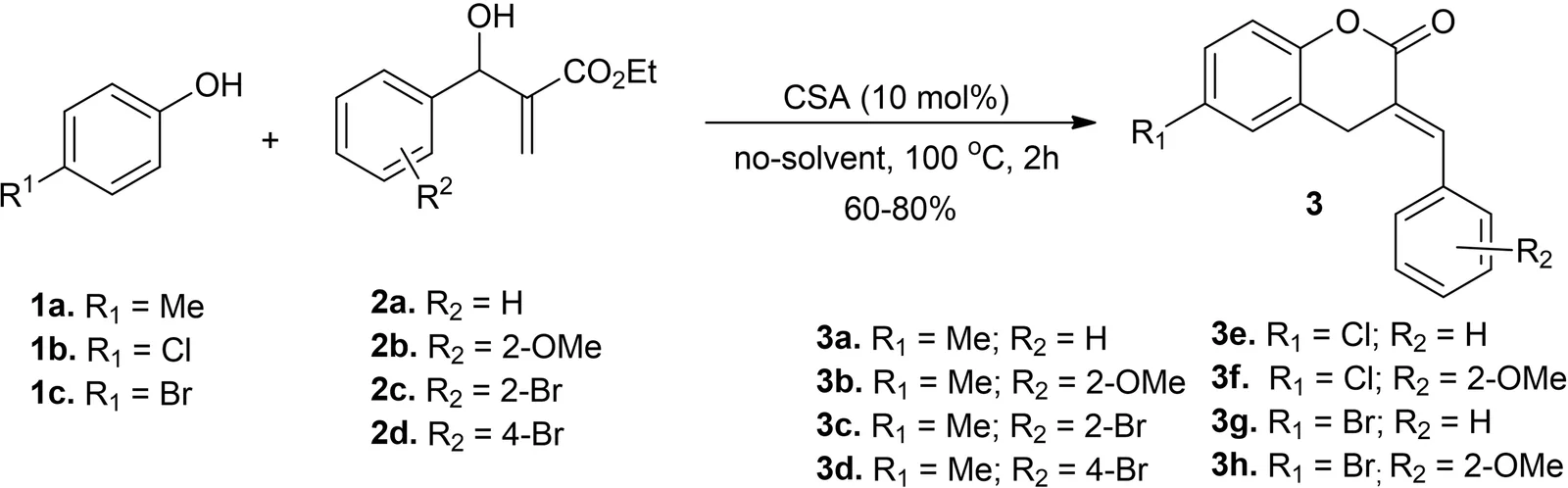
Synthesis of 3‐arylidenechroman‐2‐one.

**Scheme 3:**
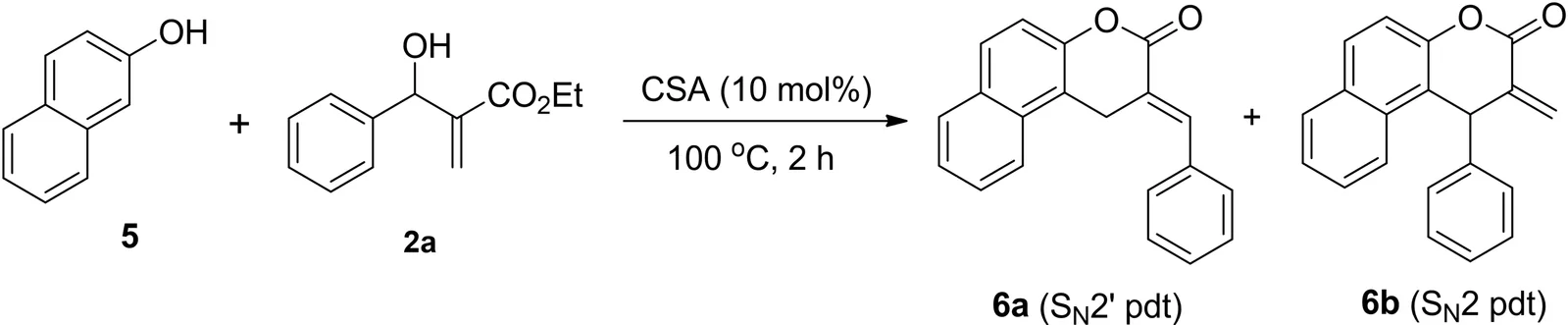
CSA catalyzed Friedel‐Crafts alkylation/lactonization of 2‐naphthol (**5**) with MBH adduct **2 a** to dihydrobenzochromen‐2‐one **6 a** and **6 b**.

Next, the scope of aforementioned CSA catalysed F−C alkylation reaction was investigated using the optimized reaction conditions. Thus, various substituted phenols (**1 a–c**) were reacted with MBH adducts (**2 a–d**) under the optimized reaction conditions (Scheme 2). In all the cases smooth reaction occurred to afford the expected 3‐arylidenechroman‐2‐one derivatives in good yields (60–80 %) (Scheme 1 and 2). However, in the case of F−C alkylation of 2‐naphthol (**5**) with **2 a**, both S_N_2’ and S_N_2 routes were operative yielding a mixture of 3‐arylidenchroman‐2‐one (**6 a**) and 4‐aryl‐3‐methylenechroman‐2‐one (**6 b**) respectively (Scheme 3). All the compounds were characterized by ^1^H and ^13^C NMR spectroscopic and mass spectrometric analyses.

**Scheme 4:**
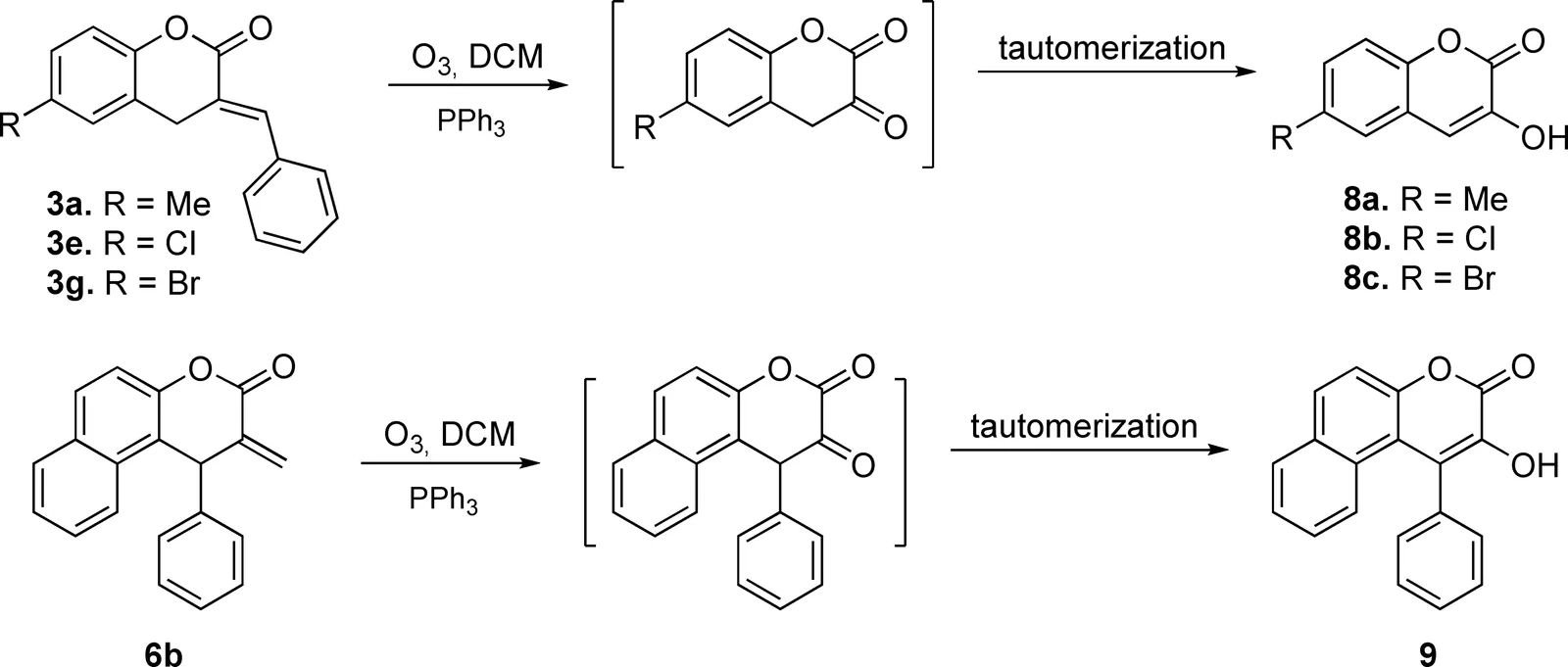
Conversion of 3‐arylidenechroman‐2‐ones to 3‐hydroxycoumarins *via* ozonolysis.

The synthesized 3‐arylidenechroman‐2‐ones are now considered as perfect precursors for the synthesis of 3‐hydroxycoumarins. Ozonolysis of **3 a** in DCM at −78 °C afforded 3‐hydroxy‐6‐methylcoumarin (**8 a**) in good yield (Scheme 4). Similarly, other 3‐arylidenechroman‐2‐ones such as **3 e** and **3 g** also yielded the expected 3‐hydroxycoumarin derivatives **8 b** and **8 c** respectively in high yields. Ozonolysis of **6 b** also yielded corresponding hydroxyl benzocoumarin **9** in good yield (Scheme 4).

Thus, an easy protocol for the synthesis of 3‐arylidene chroman‐2‐ones in good yields through the regioselective F−C alkylation of phenols with MBH adducts *via* S_N_2’ pathway under solvent‐free conditions has been developed employing catalytic amount of 10‐camphorsulfonic acid. In contrast to phenols, both S_N_2 and S_N_2’ processes were operative in the reaction of 2‐naphthol and MBH adduct to afford a mixture of 4‐aryl‐3‐methylenechroman‐2‐one and 3‐arylidenchroman‐2‐one. Synthetic utility of these compounds was demonstrated by their conversion to corresponding 3‐hydroxycoumarins *via* ozonolysis.

All the synthesized coumarin derivatives (**3 a–h, 6 a–b, 8 a–c** and **9**) were used for molecular docking studies to investigate their mode of interactions with 3CLpro enzyme.

### Molecular Electrostatic Potential (MEP)

**Figure 1:**
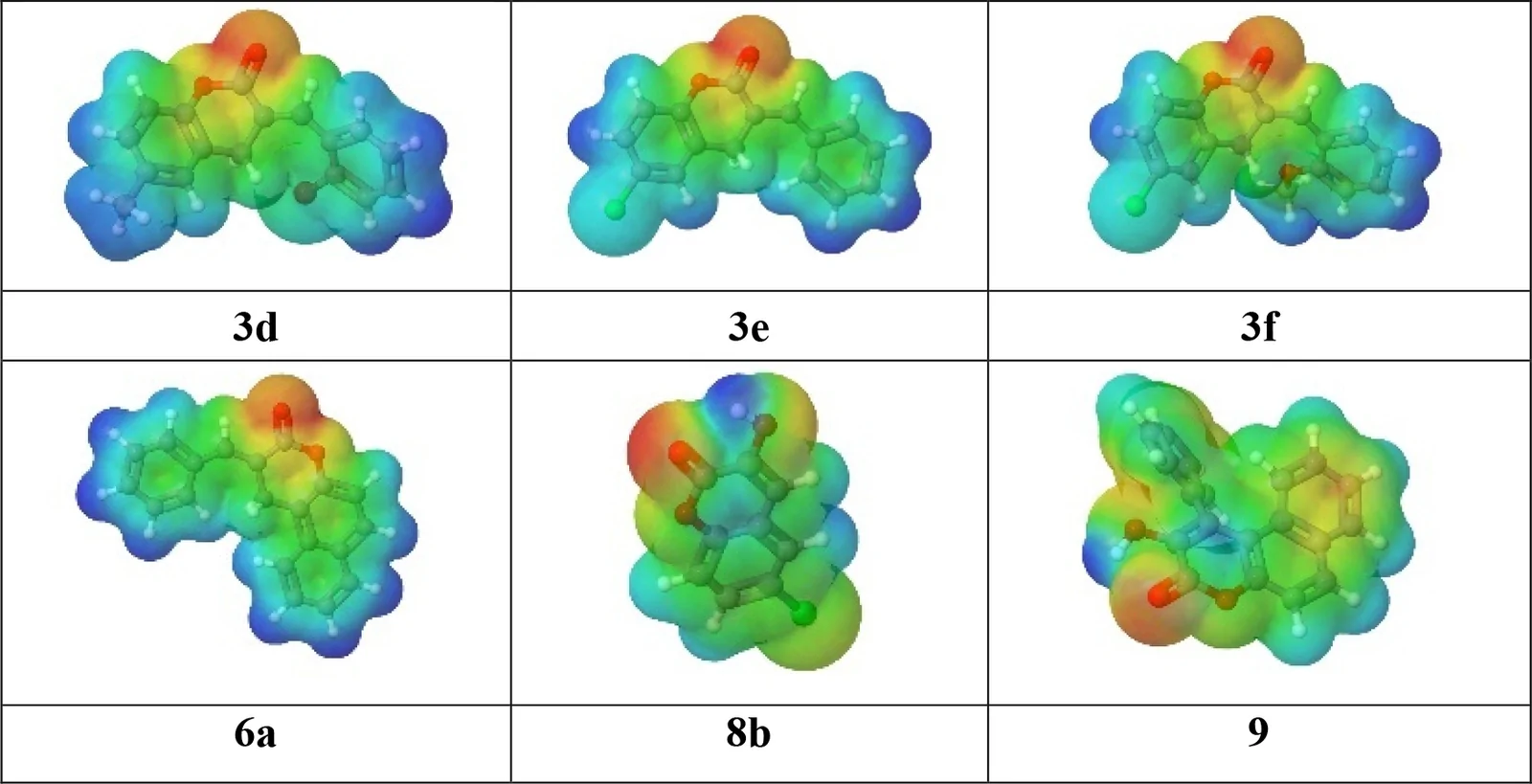
Molecular Electrostatic Potential (MEP) of compounds **3 d–f, 6 a, 8 b** and **9**.

Prior to investigate the nature of interactions between the enzyme and synthesized small molecules, their surface orientations were examined. Enzyme active sites or recognition sites are typically found on the surface of an enzyme or in a void pocket. Therefore, study of a tiny molecule's surface is crucial for binding. The Molecular Electrostatic Potential (MEP) of all the synthesized molecules (**3 a–h, 6 a–b, 8 a–c** and **9**) were assessed in this direction which have been shown in the Figures 1 and S1. The electropositive (blue), electronegative (red), and null value region (green/white) make up most of the MEP surface. It shows the donor and acceptor sides of compounds’ hydrogen bonds.^[23]^ As shown in the figure and discussed *vide infra*, the MEP surface mappings are highly useful in determining the probable binding affinities of the compounds toward the 3CLpro enzyme.

### Molecular Docking Study

#### Binding Affinity with 3CLpro

All the synthesised coumarin derivatives (**3 a–h, 6 a–b, 8 a–c, 9**) were used for docking study with the 3CLpro enzyme of the SARS‐CoV‐2 virus (PDB id 6lu7). AutoDock scores of all the compounds are listed in the Table 2. Overall span of AutoDock scores was observed to be from −5.5 kcal/mol to −8.1 kcal/mol. It is noticed that all the 3‐arylidenechroman‐2‐one (**3 a–h** and **6 a**) as well as 3‐methylenechroman‐2‐one (**6 b**) has high AutoDock scores (−6.7 to −7.8 kcal/mol). 3‐Hydroxycoumarins (**8 a–c**) showed lower AutoDock scores (~ −5.5 kcal/mol) but docking of compound **9** with 3CLpro enzyme was resulted into having maximum AutoDock score of −8.1 kcal/mol (Table 2). The active sites HIS 41, MET 165, ASP 187 and GLN 189 residues have interacted with this ligand, **9**, keeping maximum interaction distance of 4 Å (Figure 3 and Table 3). The HIS41 was observed to making π‐stacking with most of the compounds. GLN189 was also observed to interacting with all the compounds.

**Table 2:** The AutoDock score calculated after docking of compounds with 3CLpro.

Ligand	AutoDock Score [kcal/mol]	Ligand	AutoDock Score [kcal/mol]	Ligand	AutoDock Score [kcal/mol]
**3a**	−7.1	**3f**	−7.3	**8a**	−5.6
**3b**	−7.5	**3g**	−7.4	**8b**	−5.5
**3c**	−7.6	**3h**	−7.2	**8c**	−5.5
**3d**	−7.7	**6a**	−7.8	**9**	−8.1
**3e**	−7.6	**6b**	−7.5		

**Table 3:** Distance of interacting residues atoms of 3CLpro with atoms of compound **9**.

Residue	Residue atom	Ligand	Ligand atom	Distance
MET165	CB	LIG307	C13	3.3
HIS41	CB	LIG307	C1	3.9
ASP187	CB	LIG307	C6	3.8
GLN189	CG	LIG307	C9	3.6
GLN189	CG	LIG307	C13	3.5
HIS41	NE2	LIG307	O21	3.0
HIS41	π‐Stacking	LIG307	π‐Stacking PS1	4.4

#### Orientation of Compounds in the Active Site of 3CLpro

**Figure 2:**
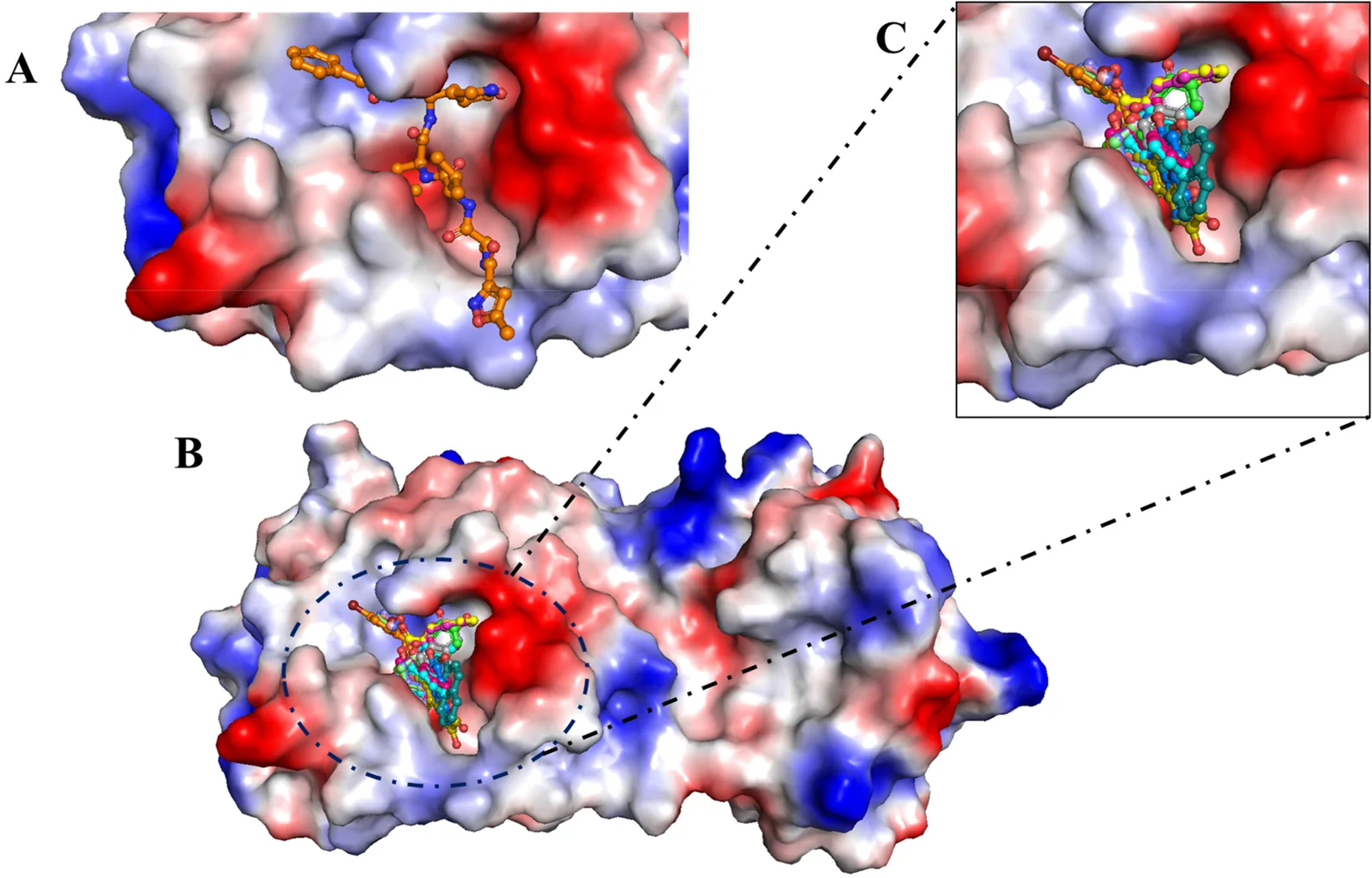
A) The inhibitor N3 (in ball‐stick form) bound structure of PDB id 6LU7. Brownish yellow colour shows a peptide part of N3. B) The docking of compounds with 3CLpro enzyme. Surface representation of the enzyme is shown along with the binding pocket inside the dotted circle. C) Catalytic domain of the enzyme showing the interactions with all the compounds.

As depicted in Figure 2B, all compounds were found to be localized in the catalytic domain of 3CLpro. The active site of the docking template, PDB id 6LU7, contains an inhibitor bound as N3 (Figure 2A). The binding energy for this peptide‐like inhibitor was reported to be −4.47 kcal/mol. The binding energy of every coumarin derivatives used in this research was higher binding affinity than −5.5 kcal/mol. Additionally, a deep cavity‐like structure was identified to be the 6LU7 catalytic site (Figure 2B). The fact that all chemicals bound together strongly proved that the catalytic pocket was well suited for them (Figure 2B, C). This domain is surrounded by electropositive and electronegative residues (Figure 2C). Similarly, the MEP of the compounds (Figure 1 and S1) had displayed a variety of electropositive and electronegative regions. It is obvious that the compounds might have a strong affinity for the 3CLpro enzyme. Interestingly, it was observed that the peptide portion of inhibitor N3 (Figure 2A) was entirely contained within the catalytic cavity. As a result, this inhibitor's interaction details have been addressed (Figure S2).^[10]^ It was identified that the peptide component interacted with the residues GLU 166, GLN 189, and THR 190. All the compounds (**3 a–h**, **6 a–b**, **8 a–c**, **9**) interacted with at least one of these residues, according to the interaction analysis. Furthermore, it was discovered that each of the compounds used in the current study was within 4 Å of the residue CYS 145, which is at 2.9 in the case of the inhibitor N3 (Figure S2).^[10]^ Further, at least one histidine (HIS41) residue was observed adjacent to the active site, and the compounds interacted with it. As a result, the interaction profile and binding affinity support the idea that a potential inhibitor might bind to the active site of 3CLpro.

**Figure 3:**
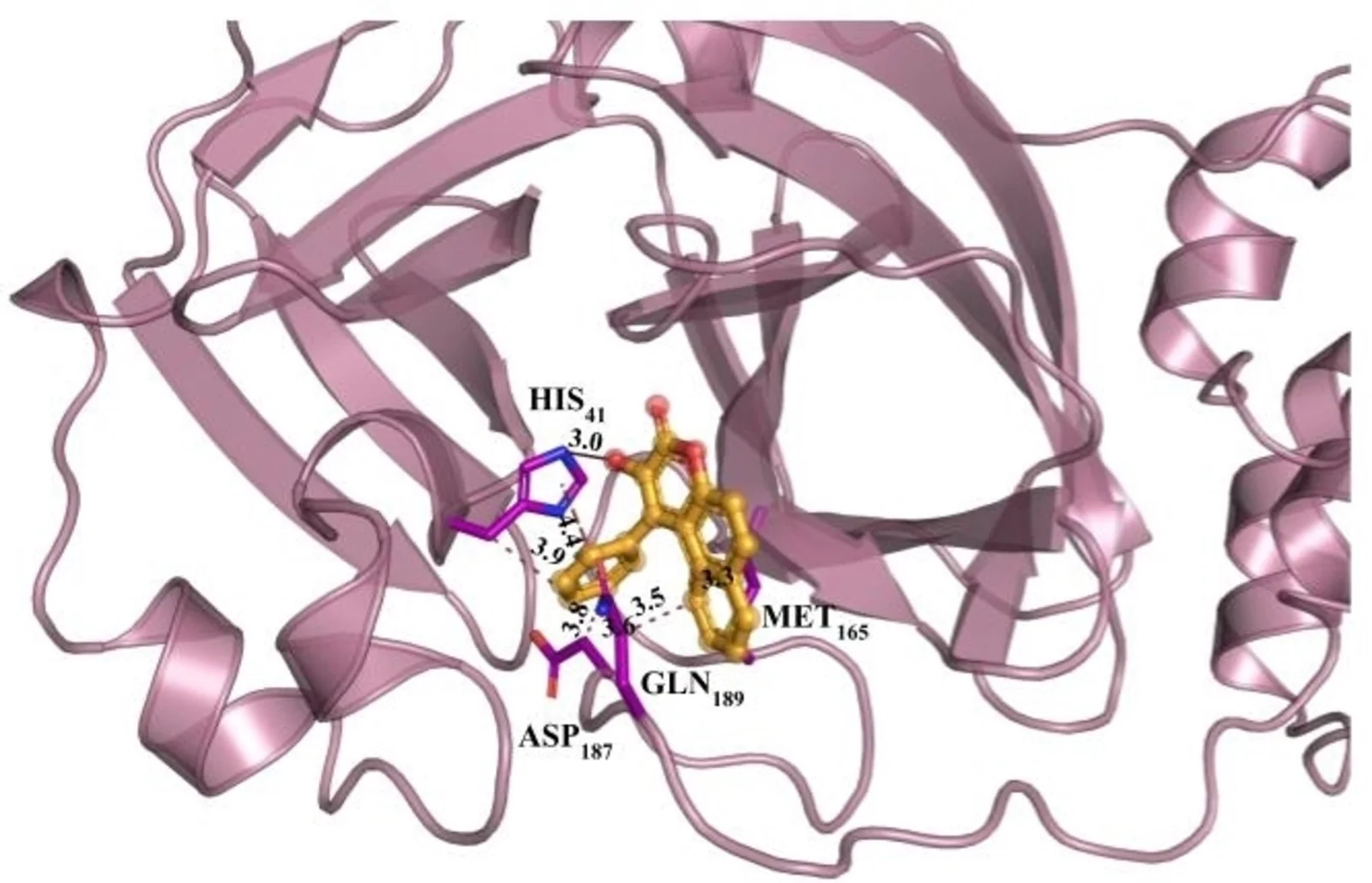
Molecular docking structure showing interaction of the compound **9** (ball‐stick representation**)** with 3CLpro enzyme (PDB 6LU7, cartoon representation). Distances of the interacting residues (in stick form) are shown in dashes.

To determine the binding profiles, the interactions between the 3CLpro enzyme and all coumarin derivatives (**3 a–h, 6 a–b, 8 a–c, 9**) were studied. Most of the residues in the inhibitor N3′s catalytic domain also displayed interactions with the compounds. The following residues were identified to be interacting with compounds: THR25, LEU27, HIS 41, MET 49, PHE 140, ASN 142, GLY143, SER 144, CYS 145, MET 165, GLU 166, ASP 187, and GLN 189. A short helix and the beta‐sheet structure surround the binding pocket (Figure S3). Additionally, by identifying residues within 4 Å, the compound interactions were studied and are listed in Table S1–S14. These compounds have reported less polar interactions. The molecule with the highest binding energy, **9** has been demonstrated with its interacting residues (Figure 3). Interactions of other compounds are shown in Figures S4–S17.

### Molecular Dynamics (MD) Simulation Analysis

#### The Backbone Variation of Protein and Complex Structures

**Figure 4:**
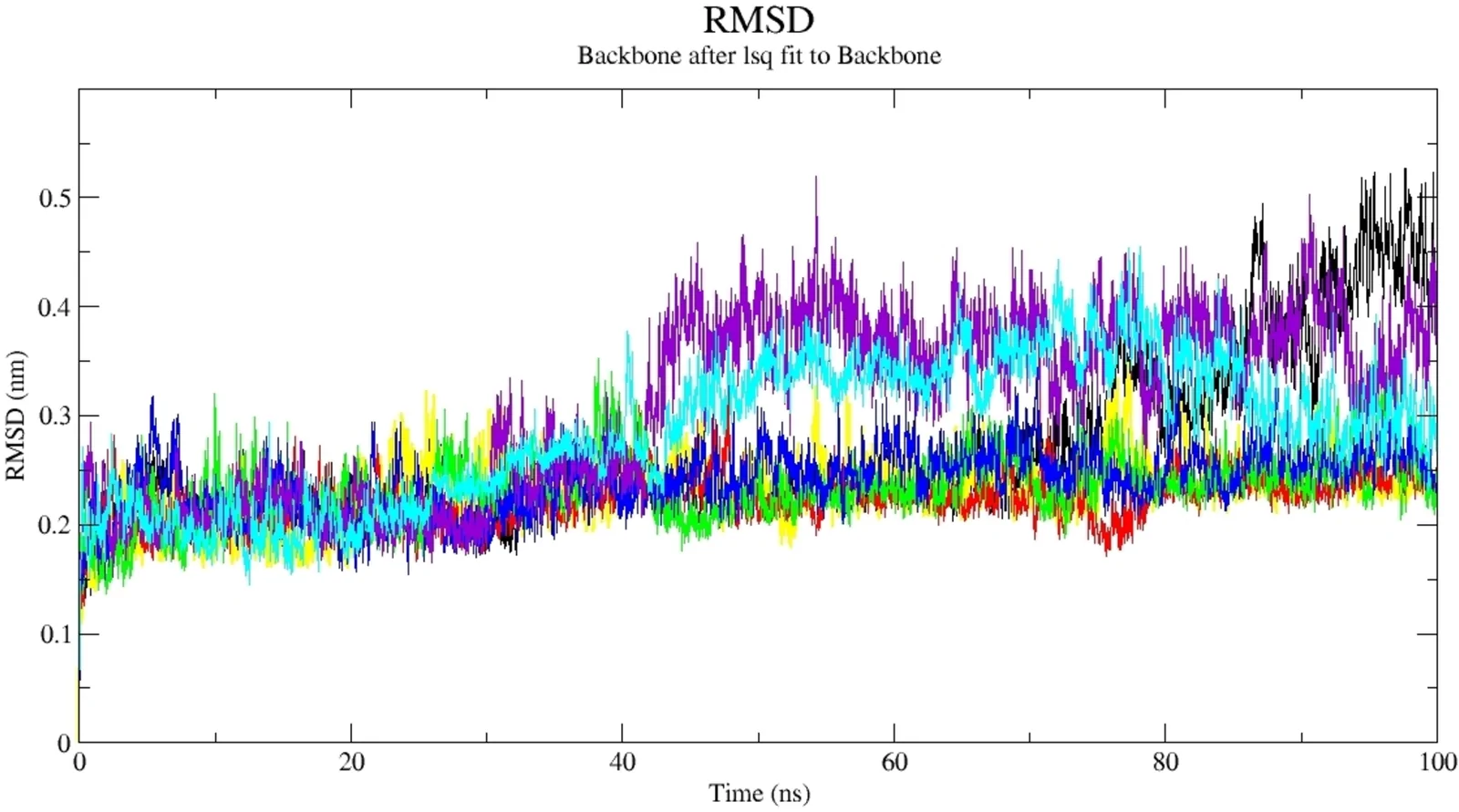
Root Mean Square Deviation (RMSD) of apo and complex structures are drawn over 100 ns simulation trajectory. The apo, complex **3 d**, complex **3 e**, complex **3 f**, complex **6 a**, complex **8 b**, and complex 9 are shown in black, yellow, red, green, blue, violet and cyan colour respectively.

Four 3‐arylidenechroman‐2‐ones (**3 d–f** and **6 a**), one 3‐hydroxycoumarin (**8 b**) and compound **9** (highest AutoDock score) were chosen for the MD simulation analysis. For the study of the dynamicity of proteins, protein backbone Root Mean Square Deviation (RMSD) is considered one of the most suited methods. An RMSD plot is shown in Figure 4, calculated for apo protein and complex structures **3 d–f, 6 a, 8 b** and **9** in the range of 0 to 100 ns. It was observed that every complex along with the apo structure has attained a stable state after 20 ns. Apo structure, shown in black, is stable at RMSD of 0.2 nm till 70 ns and fluctuates after that. Similar observation was reported previously.^[10]^ The plotted protein backbone RMSD shows greater similarity despite two complexes **8 b** and **9**, shown in violet and cyan respectively, varying 0.1 nm compared to its apo structure. Initially, the **8 b** complexes, shown in violet, stable till 40 ns and after that it jumps by 0.1 nm and then becomes stable at 0.4 nm. One another complex structure **9**, shown in cyan, is stable till 40 ns at RMSD of 0.2 nm and then fluctuates between 40 ns to 80 ns at RMSD 0.2–0.4 nm, and after that, it becomes stable at RMSD of 0.2 nm. Apart from complexes **8 b** (violet) and **9** (cyan), all other complex structures **3 d–f** and **6 a** deviates between 1.5 nm to 2.5 nm with an average RMSD of 0.2 nm and show great backbone stability. All the compound complexes, except **8 b** and **9**, the RMSD variations were within 0.1 nm have higher stability.

#### The Residue‐Wise Fluctuations over MD Trajectory

**Figure 5:**
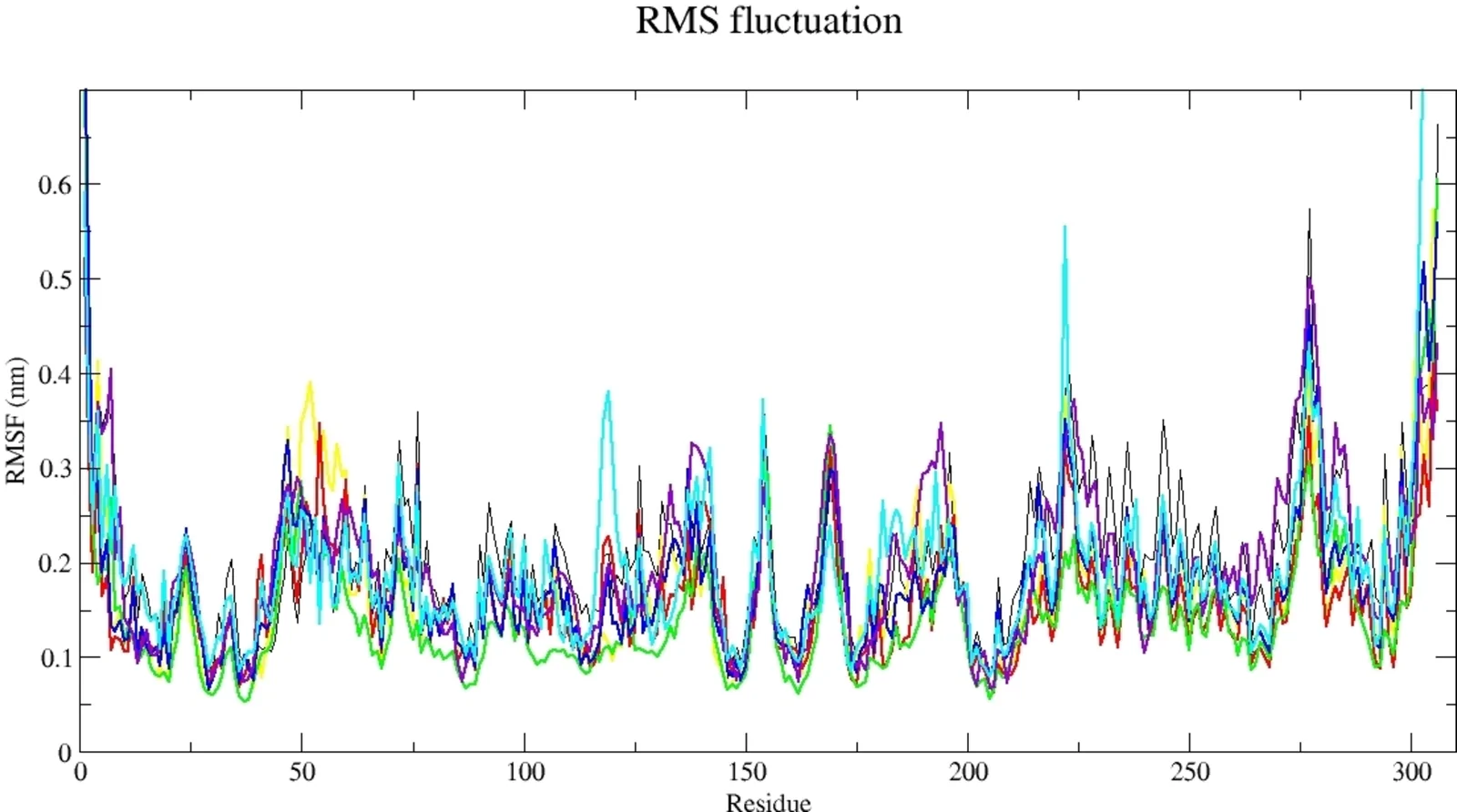
Root Mean Square Fluctuation (RMSF) of apo and complex structures are drawn over 100 ns simulation trajectory. The apo, complex **3 d**, complex **3 e**, complex **3 f**, complex **6 a**, complex **8 b**, and complex **9** are shown in black, yellow, red, green, blue, violet and cyan colour respectively.

To validate the fluctuations of protein structures of apo and its complexes, Root Mean Square Fluctuations (RMSF) play a significant role plotted for the alpha carbon, apart from the backbone RMSD. In Figure 5, RMS Fluctuation plots are shown with residues numbers. RMSF of apo simulated and averaged for each residue over time trajectories and highly fluctuated residues were identified as involved in conformational changes. In the simulated RMSF plot of apo, major peak variations have been observed in residues SER1‐ALA7, LEU75‐ARG76, ASP153‐ASP155, ASN221‐THR224, ASN274‐THR280, SER301‐GLN306 with fluctuations more than 0.35 nm. Peak fluctuations observed for complex **3 d** residues, were SER1‐LYS5, LEU50‐ASN53, TYR154, ASN221‐PHE223, MET276‐GLY278, and CYS300‐GLN306. For complex **3 e** residues were SER1‐GLY2, TYR54, TYR154, ASN277, and THR304‐GLN306. For complex **3 f**, residues were SER1‐ARG4, TYR154, GLY170 and CYS300‐GLN306. For complex **6 a**, residues were SER1‐PHE3, TYR154, ARG222, MET276‐ARG279, and SER301‐GLN306. For complex **8 b**, residues were SER1–8PHE, PHE223–225THR, LEU272–280THR, and CYS300–306GLN. For complex **9**, residues were SER1‐ARG4, TYR118‐GLY120, ASP153‐ASP155, ASN221‐PHE223, MET276‐THR280, and CYS300‐GLN306 with fluctuations more than 0.35 nm. The RMSF plotted for complex structures showed that the complex became stable after binding with the ligands.

### Radius of Gyration Analysis

**Figure 6:**
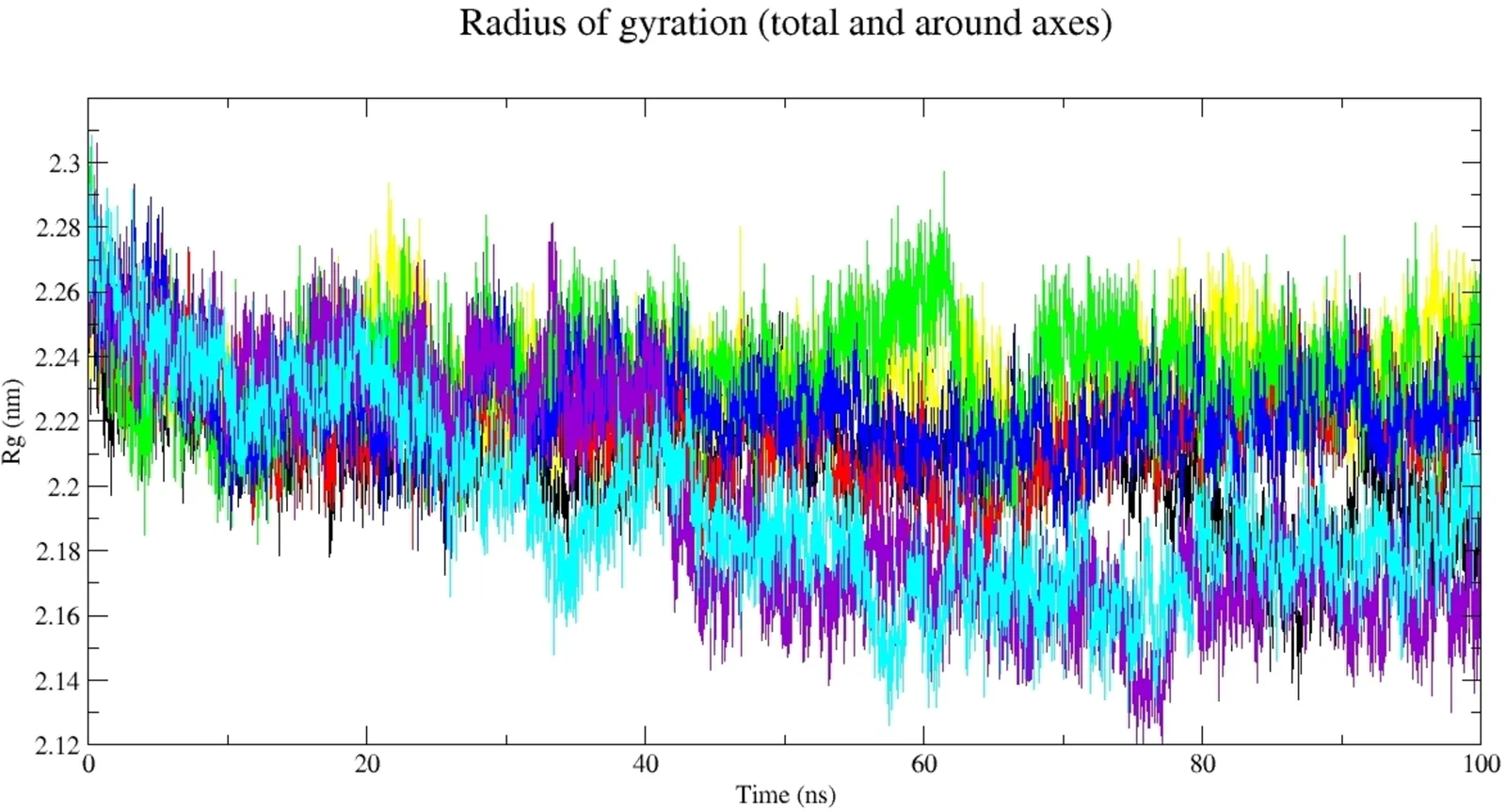
The radius of gyration plot. 100 ns trajectory was shown for comparative analysis of the Apo and the Complexes. The apo, complex **3 d**, complex **3 e**, complex **3 f**, complex **6 a**, complex **8 b**, and complex **9** are shown in black, yellow, red, green, blue, violet and cyan colour respectively.

The radius of gyration (Rg) is used to measure the compactness of the simulated structures of apo protein and its complexes with time. Rg suggested that as much as the graph gets linear consequently protein compactness increases too which means the stability of folding of the protein increases as well. In Figure 6, the radius of gyration of the apo and its complexes were shown with respect to time. The Rg graphs will not be steady with time for unfolded proteins. From Figure 6, it was observed that **8 b** (violet) and **9** (cyan) are not steady with the time compared to other complex structures (**3 d–f** and **6 a**) along with apo structure, in other words, these two complex structures (**8 b** and **9**) were considered as unfolded structures. Apart from the above, it was observed that the Rg value varies between 2.18 nm to 2.26 with an average value of 2.22 nm for both apo and complexes (**3 d–f** and **6 a**) which concludes that apo and these 4 complex structures were stable and folded.

### Hydrogen Bond Analysis

**Figure 7:**
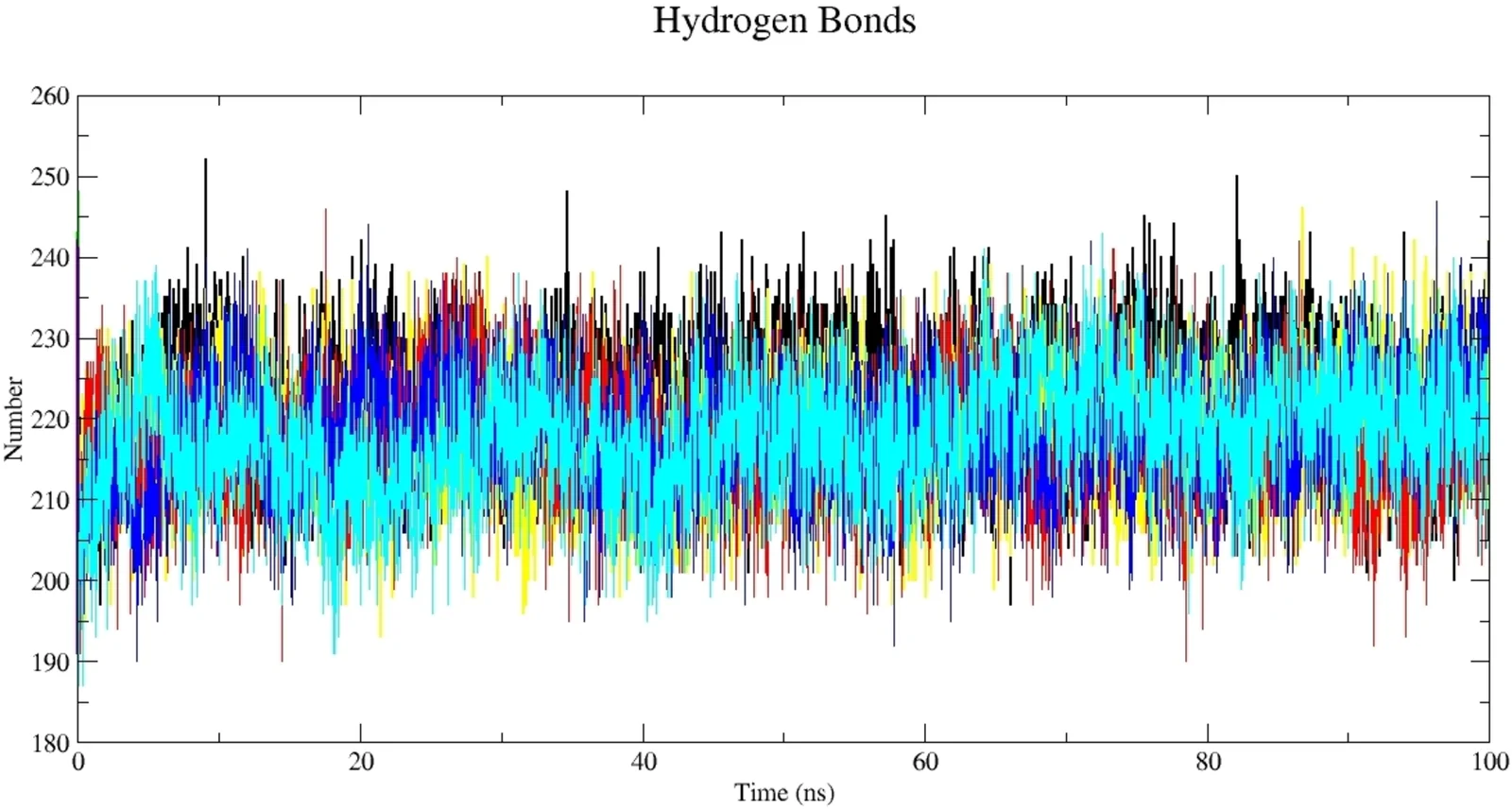
Number of hydrogen bond of apo and complex structures are drawn over 100 ns trajectory. The apo, complex **3 d**, complex **3 e**, complex **3 f**, complex **6 a**, complex **8 b**, and complex **9** are represented by black, yellow, red, green, blue, violet and cyan colour respectively.

Hydrogen bond formation is another parameter to study the interaction between the protein and ligand binding and it gives information about protein conformation and stability. It was observed that the average hydrogen bond of apo and complexes were not much change during the trajectory (Figure 7). The average number of hydrogen bonds calculated for apo, and complexes **3 d–f, 6 a, 8 b** and **9** were 224, 222, 217, 219, 218, 215 and 218 respectively. Apart from the overall hydrogen bond formation, it had been shown that the intermolecular hydrogen bonds formed in all complexes by ligands with the protein throughout the whole simulation trajectory (Figure 7). For three complexes **3 e, 3 f, 8 b** the maximum number of bonds formed by these ligands with enzyme was 3 while in all other remaining complexes, it remains 2. Therefore, it was concluded that the apo and complex structures are stable during simulation.

### Solvent Accessible Surface Area Calculation

**Figure 8:**
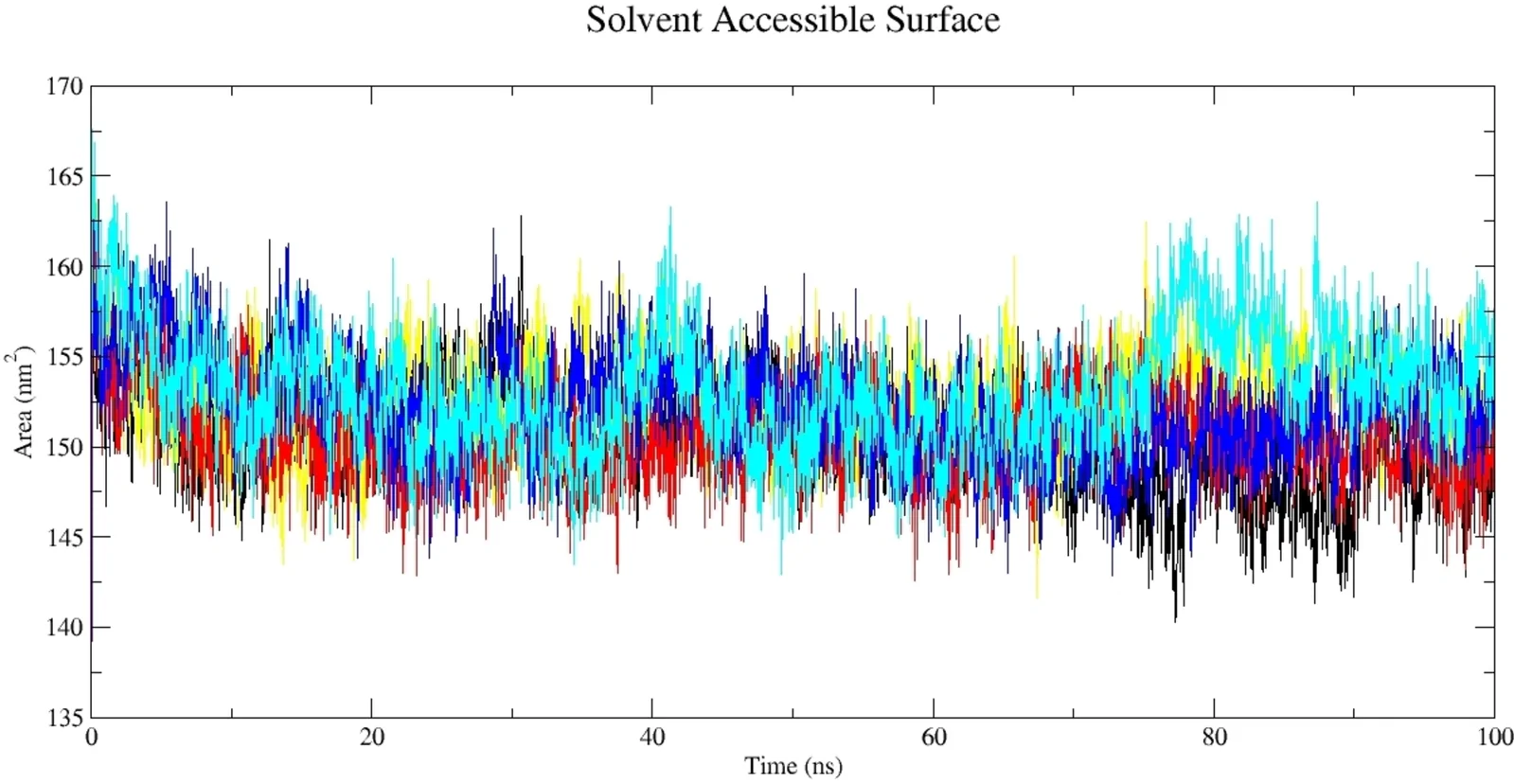
Solvent Accessible Solvent Area of apo and complex structures are drawn over 100 ns trajectory. The apo, complex **3 d**, complex **3 e**, complex **3 f**, complex **6 a**, complex **8 b** and complex **9** are represented by black, yellow, red, green, blue, violet and cyan colour respectively.

Solvent Accessible Surface Area (SASA) calculation is an analysis tool to study the interaction area between protein/complexes and solvent. SASA were plotted for all the complexes and apo structures taking the whole simulated trajectory (Figure 8). SASA values are essential to know about hydrophilicity which is increases with higher SASA values. The average SASA values for apo, complex **3 d**, complex **3 e**, complex **3 f**, complex **6 a**, complex **8 b** and **9** are 150.90 nm^2^, 152.74 nm^2^, 152.19 nm^2^, 152.60 nm^2^, 152.77 nm^2^, 149.46 nm^2^ and 151.43 nm^2^ respectively. In this study, it is observed that all complexes along with apo structure did not show much variation and varied in between 145 nm^2^ to 157 nm^2^ which is justifiable and suggests that ligand binding does not affect the folding of the protein very much. Complex **9** has low SASA value while **3 d–f, 6 a** and **8 b** are relatively higher SASA values which suggests that complexes with **3 d–f, 6 a** and **8 b** are more hydrophobic and less accessible to the solvent. It is noted that the SASA plot shows a small change and no drastic change in complexes with apo structures, which was expected for complex structures. Therefore, it may be concluded that the complexes are stable after binding with the ligands.

### ADMET Screening

**Figure 9:**
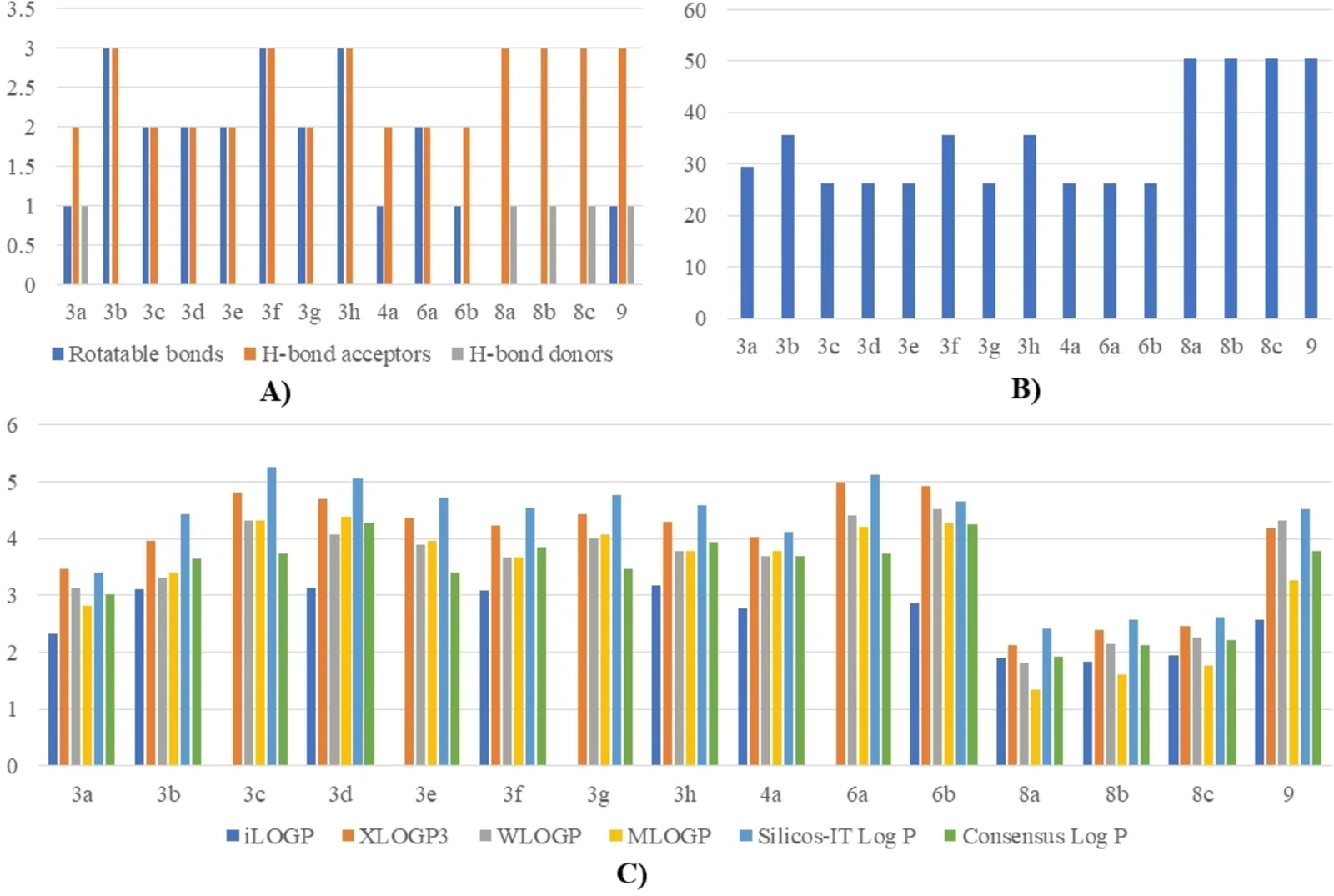
Plots for compounds **3 a–h**, **4 a**, **6 a–b**, **8 a–c** and **9**: A) Rotatable bonds, H‐bond acceptors and H‐bond donors; B) Topological Polar Surface Area (TPSA) and C) Lipophilicity.

The drug should have the ability to have therapeutic dosage value if it is designed to have potential drug efficacy. Because of this, the ADMET values are essential for determining how to filter the bioavailability criteria. The physicochemical properties of all the compounds (**3 a–h, 6 a–b, 8 a–c, 9**), have shown that their molecular weights are all less than 500 g/mol. It was seen from Figure 9A that the rotatable bonds ranged from 0 to 3. There were either two or three hydrogen bond acceptors, and they were all present in every compound. However, most compounds either have no hydrogen bond donors or have just one (Figure 9A). Figure 9B depicts the Topological Polar Surface Area (TPSA) plot, which reveals that all compounds have values between 26 and 50 Å^2^. According to a report, the maximum TPSA value should be 140 Å^2^, which is the acceptable level of cell permeability for all chemicals that are absorbed through the cell membrane.^[24]^ A measure of a chemical compound's affinity for lipids is called lipophilicity. Understanding lipophilicity is crucial because it contains information that helps a substance permeate a cell and bind to a receptor.^[25]^ As a result, it has significant pharmacokinetic importance. It is impacting drug degradation, solubility, reactivity, and other factors. By using the iLOGP,^[26]^ XLOGP3,^[27]^ WLOGP,^[28]^ MLOGP,^[29]^ and SILICOS‐IT^[30]^ techniques, the partition coefficient between n‐octanol and water (log Po/w) was determined (Figure 9c). The arithmetic mean of all these values is regarded as the consensus value. These numbers were primarily in the range of 2.45 to 6.61 while the consensus fell between 1.92 and 4.25. This demonstrates that oral absorption is effective for all compounds.

The solubility of organic compounds in water is defined by the aqueous or water solubility, or Log S, which affects the transport, absorption, distribution, and bioavailability of drugs.^[31]^ Through the ESOL,^[32]^ Ali^[9]^ and Silicos‐IT^[30]^ methodologies, it has been predicted (Table 4) that compounds **8 a–c** would exhibit solubility (S), while the others are classified as moderately soluble (MS) or poorly soluble (PS). This suggests that the MS and S compounds could potentially be administered orally or through parenteral routes. All of the drugs in the pharmacokinetics study had substantial gastrointestinal absorption, making them excellent candidates for oral administration (Table 5).

**Table 4:** Water solubility from three different methods, ESOL, Ali and Silicos‐IT program. Pharmacokinetics includes gastro intestinal absorption, p‐glycoprotein substrate and skin permeability.

Molecule	ESOL Log S	ESOL Class	Ali Log S	Ali Class	Silicos‐IT LogSw	Silicos‐IT class
**3a**	−3.88	Soluble	−3.76	Soluble	−4.69	Moderately soluble
**3b**	−4.32	Moderately soluble	−4.42	Moderately soluble	−6.06	Poorly soluble
**3c**	−5.22	Moderately soluble	−5.08	Moderately soluble	−6.98	Poorly soluble
**3d**	−5.16	Moderately soluble	−4.97	Moderately soluble	−6.76	Poorly soluble
**3e**	−4.61	Moderately soluble	−4.64	Moderately soluble	−6.38	Poorly soluble
**3f**	−4.61	Moderately soluble	−4.69	Moderately soluble	−6.28	Poorly soluble
**3g**	−4.93	Moderately soluble	−4.7	Moderately soluble	−6.6	Poorly soluble
**3h**	−4.93	Moderately soluble	−4.76	Moderately soluble	−6.49	Poorly soluble
**6a**	−5.17	Moderately soluble	−5.28	Moderately soluble	−7.43	Poorly soluble
**6b**	−5.2	Moderately soluble	−5.21	Moderately soluble	−6.97	Poorly soluble
**8a**	−2.84	Soluble	−2.82	Soluble	−3.42	Soluble
**8b**	−3.13	Soluble	−3.09	Soluble	−3.66	Soluble
**8c**	−3.45	Soluble	−3.15	Soluble	−3.9	Soluble
**9**	−4.87	Moderately soluble	−4.96	Moderately soluble	−7.22	Poorly soluble

**Table 5:** Pharmacokinetics study includes gastrointestinal absorption, p‐glycoprotein substrate, binding with metabolic enzymes and skin permeability.

Molecule	GI absorption	BBB permeant	Pgp substrate	CYP1A2 inhibitor	CYP2C19 inhibitor	CYP2C9 inhibitor	CYP2D6 inhibitor	CYP3A4 inhibitor	log Kp (cm/s)
**3a**	High	Yes	Yes	Yes	No	No	Yes	No	−5.22
**3b**	High	Yes	No	No	Yes	Yes	Yes	No	−5.2
**3c**	High	Yes	Yes	Yes	No	No	No	No	−4.91
**3d**	High	Yes	No	Yes	Yes	Yes	No	No	−4.99
**3e**	High	Yes	Yes	Yes	No	No	No	No	−4.85
**3f**	High	Yes	No	Yes	Yes	Yes	Yes	No	−5.14
**3g**	High	Yes	Yes	Yes	No	No	No	No	−5.08
**3h**	High	Yes	No	Yes	Yes	Yes	Yes	No	−5.36
**6a**	High	Yes	Yes	Yes	No	No	No	No	−4.51
**6b**	High	Yes	No	Yes	Yes	Yes	No	No	−4.57
**8a**	High	Yes	No	Yes	No	No	No	No	−5.86
**8b**	High	Yes	No	Yes	No	No	No	No	−5.8
**8c**	High	Yes	No	Yes	No	No	No	No	−6.03
**9**	High	Yes	No	Yes	Yes	No	No	No	−5.08

All the compounds (**3 a–h, 6 a–b, 8 a–c, 9**) have been discovered to have appropriate blood‐brain barrier (BBB) permeability,^[8]^ indicating that they have good drug distribution indices. They may be actively pumped through the cell because they were all discovered to be Pgp substrates.^[33]^ In Table 5, the potential for binding with the metabolic enzymes CYP1 A2, CYP2 C19, CYP2 C9, CYP2 D6, and CYP3 A4 inhibitors has been listed. They all demonstrated degradation by at least one enzyme. Additionally, it had been projected that log Kp,^[34]^ a measure of skin permeability, would be less permeable at lower levels.

**Table 6:** The Drug Likeness criteria evaluation.^[a]^

Molecule	Lipinski	Ghose	Veber	Egan	Muegge	Bioavai‐lability Score	Synthetic Accessib‐ility	Toxicity	Toxicity Alert
**3a**	0	0	0	0	0	0.55	2.78	Accepted	
**3b**	0	0	0	0	0	0.55	3.06	Accepted	
**3c**	1	0	0	0	0	0.55	3.16	Intermediate	Low_Risk_halogenure, Low_Risk_coumarines
**3d**	1	0	0	0	0	0.55	3.13	Intermediate	Low_Risk_halogenure
**3e**	0	0	0	0	0	0.55	3.07	Intermediate	Low_Risk_halogenure, Low_Risk_coumarines
**3f**	0	0	0	0	0	0.55	2.99	Intermediate	Low_Risk_halogenure
**3g**	0	0	0	0	0	0.55	3.08	Intermediate	Low_Risk_halogenure, Low_Risk_coumarines
**3h**	0	0	0	0	0	0.55	3.11	Intermediate	Low_Risk_halogenure
**6a**	1	0	0	0	0	0.55	3.26	Intermediate	Low_Risk_coumarines
**6b**	1	0	0	0	0	0.55	3.35	Accepted	
**8a**	0	0	0	0	1	0.55	2.51	Intermediate	Low_Risk_coumarines
**8b**	0	1	0	0	1	0.55	2.52	Intermediate	Low_Risk_halogenure, Low_Risk_coumarines
**8c**	0	1	0	0	0	0.55	2.67	Intermediate	Low_Risk_halogenure, Low_Risk_coumarines
**9**	0	0	0	0	0	0.55	3.17	Intermediate	Low_Risk_coumarines

[a] Lipinski, Ghose, Veber, Egan and Muegge are rules where 0 stands for yes (follows rule) and the integer number suggests the violation of that many number of criteria's from the rule. Bioavailability score is Abbott BS. Synthetic Accessibility defines ease of synthesis from range 1 to 10 (easy‐difficult). The toxicity indicated toxicity level and alert has been noted.

The evaluation of a drug's drug‐likeness is also based on the chemical structures and physicochemical characteristics. Lipinski,^[35]^ Ghose,^[36]^ Veber,^[37]^ Egan,^[38]^ and Muegge^[39]^ methodologies had been used to conduct this evaluation (Table 6). The findings showed that compounds **3 a–b** and **3 e–h** adhere to all the rules and **3 c–d, 6 a–b, 8 a, 8 c** and **9** violets only one except compound **8 b** which breaks two of them. This suggests that each compound has drug‐like properties. Additionally, each of the chemicals has a strong potential for being a therapeutic candidate due to its sufficient bioavailability score. The scores for synthetic accessibility had also been shown, and it had been determined that it was moderately easy to synthesis these molecules. Furthermore, none of the chemicals tested positive for pan‐assay interference compounds (PAINS), despite all of them being scanned. Finally, the toxicology findings showed that all fifteen compounds have successfully incorporated the toxicities listed in Table 6. Overall, ADMET concludes that the compounds are primarily drug‐like and not harmful.

## Conclusions

In conclusion, an easy and practical method for the synthesis of 3‐hydroxycoumarins has been developed *via* Friedel‐Crafts alkylation of phenols with MBH adduct using camphor‐10‐sulfonic acid as catalyst. In‐silico methods were employed to scrutinize the pharmacokinetic properties (ADMET), charge distribution (MEP), and interaction efficacy with the 3CLpro enzyme of SARS‐CoV‐2 for the synthesized molecules. The ADMET analysis of flavonoids revealed that most compounds exhibited drug‐like properties. Utilizing Autodock Vina software for molecular docking, the interactions of fifteen synthesized compounds with the 3CLpro enzyme (PDB ID 6LU7) were investigated. Compound **9** exhibited the highest AutoDock score of −8.1 kcal/mol in its docking with 3CLpro. Active site residues, namely HIS 41, MET 165, ASP 187, and GLN 189, interacted with ligand **9**, maintaining a maximum interaction distance of 4 Å (Figure 3 and Table 3).

Additionally, molecular dynamics simulations were conducted for selected compounds over 100 ns each (total computational timeof 700 ns). Analysis techniques, including root mean square deviation (RMSD), root mean square fluctuation (RMSF), hydrogen bonds, radius of gyration, and solvent‐accessible surface area (SASA), were employed to assess the conformational and structural stability of the compounds with the protein. The RMSD comparison, focusing on backbone variation, indicated that complexes **8 b** (violet) and **9** (cyan) deviated between 1.5 nm to 2.5 nm, with an average RMSD of 0.2 nm, demonstrating significant backbone stability. All compound complexes, except **8 b** and **9**, exhibited RMSD variations within 0.1 nm, indicating greater stability.

Residual fluctuation analysis highlighted that compounds **8 b** and **9** displayed more fluctuation compared to **3 d**–**f** and **6 a**, with reference to the apo structure. Assessing system compactness using Rg revealed that **8 b** (violet) and **9** (cyan) were less steady over time compared to other complex structures (**3 d‐**‐**f** and **6 a**) and the apo structure. Solvent interactions, evaluated by SASA, demonstrated minimal changes with no drastic alterations in complexes compared to apo structures, aligning with expectations for complex structures. Therefore, it is concluded that the complexes remain stable after binding with ligands. Compound **3 d‐**‐**f** and **6 a** exhibited the most stable behaviour, with **3 f** standing out among all. Conversely, compounds **8 b** and **9** displayed more dynamic behaviour, indicating fluctuation. Hence, compounds **3 d**–**f** and **6 a**, especially **3 f**, demonstrated superior binding against the target enzyme of SARS‐CoV‐2, suggesting their potential use as inhibitors against 3CLpro.

## Materials and Methods

### Synthesis

Detailed synthesis procedures and the characterization data of all the newly synthesized molecules are given in the supporting information.

### Structural Preparation and ADMET Screening for *In‐Silico* Study

ChemDraw Ultra^[40]^ was used to construct the small molecule in‐silico structure in 2D. Then, energy minimization was done using the built‐in tool of the Chem3D tool,^[41]^ which was used to obtain the three‐dimensional structures of the 2D structures. The SARS‐Cov‐2 3CLpro enzyme, on the other hand, was obtained from RCSB using PDB ID 6LU7.^[42]^ Its apo form of protease was isolated from it and minimised using GROMACS,^[43]^ an open‐source programme running on Linux. Calculations for minimization had been performed using GROMOS96 54a7 all‐atom force fields.^[44]^ The molecular Gasteiger charges were computed in order to calculate the Molecular Electrostatic Potential Surfaces (MEPS), and JMOL was used to view the MEP surfaces.^[45]^ Small molecule SMILEs were extracted from SDF structures using the OpenBabel software.^[46]^ Then, a Swiss ADME online web server^[47]^ was used to assess absorption, distribution, metabolism, and excretion (ADME). Utilizing the toxicophores from FAF‐Drug 4,^[48]^ the toxicity screening was carried out.

### Molecular Docking of Small Molecules with Target 3CLpro

The AutoDock Vina was used to perform docking of small molecules against SARS CoV‐2 target. AutoDock Tools was used for generation of pdbqt files for enzyme target and small molecules. The grid parameters were decided to be 64.039, 46.852, and 58.802 for the box's centre along with the box length on the x, y, and z dimensions were set to be 19, 22, and 20 respectively. One Angstrom was used as the spacing. For the docking analysis, the number of modes was chosen to be 10, The docking was performed using Autodock Vina software (https://vina.scripps.edu/). The interactions were investigated using PLIP webserver (https://plip‐tool.biotec.tu‐dresden.de/plip‐web/plip/index) and PyMOL^[49]^ was used for visualization.

### Molecular Dynamics (MD) Simulations

An all‐atom Molecular Dynamics (MD) simulation was performed to study the investigation of the structural dynamics of the enzyme 3‐chymotrypsin‐like cysteine protease (3CLpro) and their complexes. In this study, an online server WebGro (https://simlab.uams.edu/index.php) based on the Gromacs‐2019.2 program was used to simulate and study the thermodynamic stability of the protein and its complexes. The topology creation was performed using two separate methods: one is the protein topology using GROMOS96 54a7 all‐atom force fields,^[44]^ and the second is the topology of the ligands using the PRODRG 2.5 server^[50]^ before the start of the simulation. A simple point charge (SPC) water model was used to solvate the system. A periodic boundary condition (PBC) was applied to the system with a unit cubic box having each side equal to 20 Å. An appropriate number of ions (Na & Cl) are added to neutralize the system with 0.15 M salt concentration. Energy minimization of the system is processed to stabilize the protein and ligand. During energy minimizations, the steepest descent algorithm was used having iterations with a time step size of 500,000 ps. The algorithm used has a cut‐off of 1000 kJmol^−1^. After proper energy minimization of the solvated system, MD simulation was performed. Before the start of the production MD run, two ensemble processes are carried out to equilibrate the system, one is the NVT ensemble and the second is the NPT ensemble. The NVT ensemble was applied to equilibrate the system thermodynamically. In the NVT equilibration process, a constant number of particles (N), constant volume (V), and constant pressure are applied as boundary conditions which have a time step size of 2 fs with a total of 150,000 steps. In NPT equilibration processes boundary conditions are applied at constant pressure (P) of 1 atm and constant temperature (T) of 300 K, often known as an isothermal‐isobaric ensemble. During NPT equilibration, 150,000 steps are taken with a time step size of 2 fs. After proper minimization and equilibration of apo and complexes, final production MD run was performed for 100 ns.

## Conflict of Interests

The authors declare no conflict of interest.

## Supporting information

As a service to our authors and readers, this journal provides supporting information supplied by the authors. Such materials are peer reviewed and may be re‐organized for online delivery, but are not copy‐edited or typeset. Technical support issues arising from supporting information (other than missing files) should be addressed to the authors.

Supporting Information

## Data Availability

The data that support the findings of this study are available in the supplementary material of this article.
