# Supplementary material for: A Facile Synthesis of 3‐Substituted Coumarins and Investigation of Their 3CLpro Inhibition Activity Against SARS‐CoV‐2
Source: ChemistryOpen. 2024 Nov 26;14(5):e202400319. doi: 10.1002/open.202400319 (PMC13580852; doi:10.1002/open.202400319)
Supplement: Supplementary file 1 [file OPEN-14-e202400319-s001.pdf]

ChemistryOpen

Supporting Information

A Facile Synthesis of 3-Substituted Coumarins and Investigation of Their 3CLpro Inhibition Activity Against SARS-CoV-2

Manoj K. Choudhary, Khalid Ansari, Vivek Junghare, Sandip K. Nayak, Saugata Hazra,* and Soumyaditya Mula*

Table of content

Sl. No.	Title	Page No.
1	Materials and General Methods	S2
2	Synthetic procedure	S2-S11
3	Molecular Electrostatic Potential (MEP) study	S12
4	Docking and Molecular Dynamics simulation Study	S13-S27
5	NMR spectra	S28-S45
6	HRMS spectra	S46-S48

1. Materials and General Methods.

Chemical reactions were carried under argon atmosphere using anhydrous solvents in round bottom flask fitted with rubber septum or screw-cap schlenk tube with magnetic stirring unless stated otherwise. Reagents were obtained from commercial supplier and were used without further purification.

Reactions were monitored by Thin-layer chromatography which was performed in commercially available 0.25 mm fluorescent silica plate (F-254) and visualized using a UV-lamp (254 nm wavelength) or developed in iodine chamber or in alkaline $KMnO_4$ solution after heating. Normal phase flash chromatography was performed for separation using silica gel (40-63 μm) and eluted with distilled solvents.

NMR spectra were recorded in 500 MHz Varian FT-NMR instruments as well as in 200 MHz Bruker FT-NMR instruments. Peak positions (δ values) were referenced using residual non-deuterated solvent (7.26 ppm for ^1H signal and 77.00 ppm for ^{13}C signal of residual $CHCl_3$ present in $CDCl_3$ solvent) as an internal standard. The following abbreviations are used to express multiplicity of ^1H NMR signals: s = singlet, br = broad signal, d = doublet, t = triplet, q = quartet, m = multiplet. Proton coupling constants (*J* values) are expressed in Hertz (Hz). Yields represent to chromatographically and spectroscopically (^1H NMR) homogeneous materials. High Resolution Mass Spectrometric (HRMS) analysis was done using either 6540 UHD Accurate-Mass Agilent Q-TOF LC/MS instrument or Bruker Maxis Impact Q-TOF LC/MS instrument. Spectroscopic data is in agreement with all known compounds.

2. Synthetic procedure.

General procedure for the preparation of Morita Baylis-Hillman (MBH) adducts

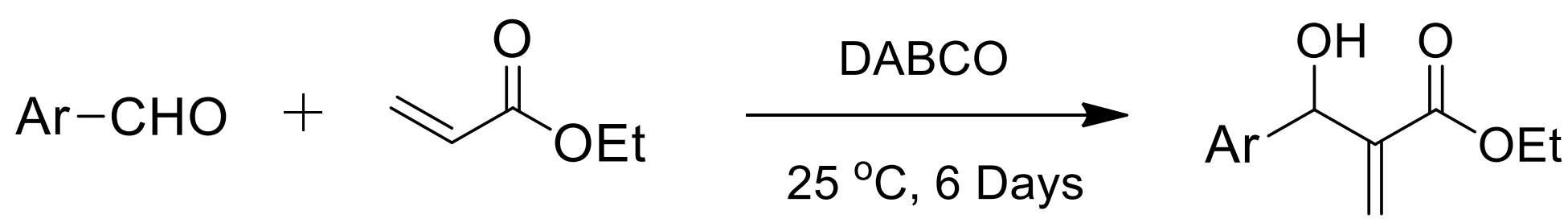

10a. R = Phenyl
10b. R = o-methoxyphenyl
10c. R = o-bromophenyl
10d. R = p-bromophenyl

2a. R = Phenyl
2b. R = o-methoxyphenyl
2c. R = o-bromophenyl
2d. R = p-bromophenyl

Scheme S1. Synthesis of Morita Baylis-Hillman (MBH) adducts (**2a-d**).

A mixture of aromatic aldehyde (**10a-d**, 10 mmol), ethyl acrylate (12 mmol), and DABCO (1 mmol, 10 mol %) was stirred at ambient temperature for 6 days. When aldehydes were almost consumed (checked by TLC), the reaction mixture was extracted with ethyl acetate. The solvent was removed under *vacuo*, and the residue was purified by column chromatography (silica-gel, EtOAc: petroleum ether) to afford the desired adducts **(2a-d)**.

Ethyl 3-hydroxy-3-phenyl-2-methylenepropanoate (2a)

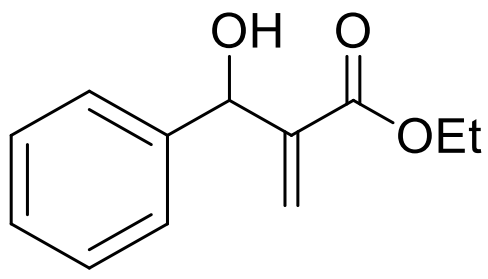

Yield: 1.79 g (87 %); ^{1}H NMR (200 MHz, $CDCl_3$): δ 7.41–7.30 (m, 5H), 6.38 (s, 1H), 5.85 (s, 1H), 5.60 (s, 1H), 4.21 (q, J = 7.2 Hz, 2H), 2.77 (br s, 1H), 1.28 (t, J = 7.2 Hz, 3H); ^{13}C NMR (50 MHz, $CDCl_3$): δ 166.1, 142.2, 141.3, 128.2, 127.6, 126.5, 125.4, 72.8, 60.7, 13.8; IR (neat): 3501, 1728 cm^{-1}; MS (*m*/*z*): 206.1 (M^+).

Ethyl 3-hydroxy-3-(2-methoxyphenyl)-2-methylenepropanoate (2b)

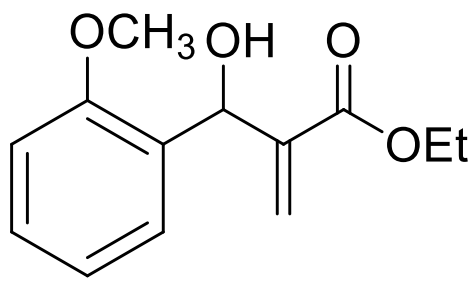

Yield: 1.96 g (83%); ^{1}H NMR (200 MHz, $CDCl_3$): δ 7.42–7.27 (m, 2H), 7.04–6.90 (m, 2H), 6.34 (s, 1H), 5.92 (s, 1H), 5.76 (s, 1H), 4.23 (q, J = 7.2 Hz, 2H), 3.87 (s, 3H), 2.88 (br s, 1H), 1.30 (t, J = 7.2 Hz, 3H); ^{13}C NMR (50 MHz, $CDCl_3$): δ 166.4, 156.4, 141.6, 129.1, 128.6, 127.4, 125.1, 120.4, 110.3, 67.7, 60.5, 55.1, 13.8; IR (neat): 3484, 1719 cm^{-1}; MS (*m*/*z*): 236.1 (M^+).

Ethyl 3-(2-bromophenyl)-3-hydroxy-2-methylenepropanoate (2c)

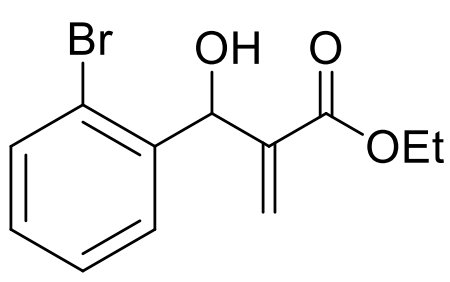

Yield: 2.42 g (85%); ^{1}H NMR (200 MHz, $CDCl_3$): δ 7.61–7.39 (m, 2H), 7.35–7.16 (m, 2H), 6.39 (s, 1H), 5.99 (s, 1H), 5.61 (s, 1H), 4.27 (q, J = 7.2 Hz, 2H), 2.72 (br s, 1H), 1.32 (t, J = 7.2 Hz, 3H); ^{13}C NMR (50 MHz, $CDCl_3$): δ 166.4, 140.9, 139.9, 132.6, 129.2, 128.3, 127.5, 126.7, 123.1, 71.4, 61.0, 14.0; IR (neat): 3419, 1732 cm^{-1}; MS (*m*/*z*): 284.1 (M^+).

Ethyl 3-(4-bromophenyl)-3-hydroxy-2-methylenepropanoate (2d)

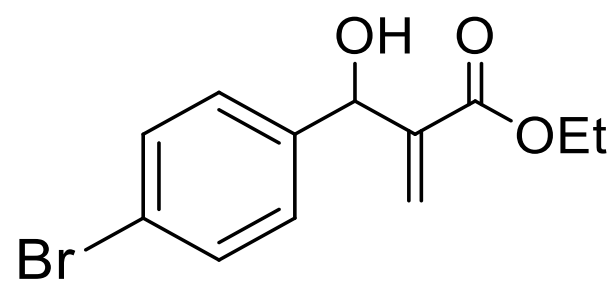

Yield: 2.45 g (86%); ^{1}H NMR (500 MHz, $CDCl_3$): δ 7.47 (d, J = 8.0 Hz, 2H), 7.25 (d, J = 8.0 Hz, 2H), 6.34 (s, 1H), 5.81 (s, 1H), 5.50 (s, 1H), 4.18 (q, J = 7.5 Hz, 2H), 2.65 (br s, 1H), 1.26 (t, J = 7.5 Hz, 3H); ^{13}C NMR (125 MHz, $CDCl_3$): δ 166.2, 141.8, 140.4, 131.5, 128.3, 126.1, 121.7, 72.8, 61.1, 14.0; IR (neat): 3422, 1730 cm^{-1}; MS (*m*/*z*): 284.0 (M^+).

Typical procedure for preparation of 3-benzylidene-6-methylchroman-2-one (3a)

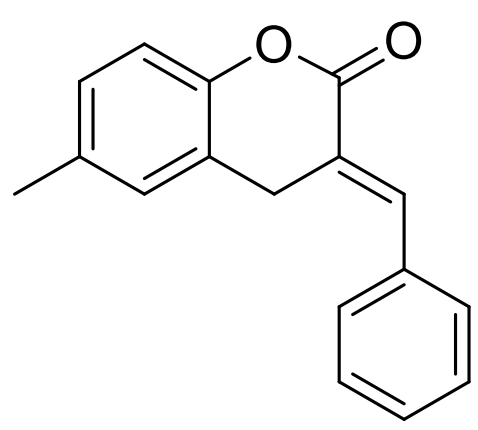

A mixture of 4-methylphenol (**1a**, 2.0 mmol), MBH alcohol (**2a**, 2.2 mmol), and CSA (10 mol %) without any solvent was heated at 100 °C under stirring for 2 h when **1a** was fully consumed (checked by TLC). The reaction mixture was quenched with saturated aqueous $NaHCO_3$ solution (1 mL), and the viscous mass thus obtained was extracted with EtOAc. The organic layer was washed with water, brine and dried (Na_2SO_4). The solvent was removed under *vacuo*, and the residue was purified by column chromatography (silica-gel, EtOAc-petroleum ether) to get the desired product. The product was recrystallized (chloroform-methanol) to furnish pure **3a** as colourless crystals.

Yield: 0.402 g (80%); mp: 120-121°C; ^{1}H NMR (500 MHz, $CDCl_3$): δ 7.96 (t, *J* = 2.5 Hz, 1H), 7.49–7.40 (m, 5H), 7.03 (d, *J* = 8.5 Hz, 1H), 6.96 (d, *J* = 8.5 Hz, 2H), 4.02 (d, *J* = 2.5 Hz, 2H), 2.89 (s, 3H); ^{13}C NMR (125 MHz, $CDCl_3$): δ 164.4, 148.2, 142.2, 134.5, 133.9, 130.0, 129.4, 128.5, 128.4, 123.1, 119.7, 116.5, 28.7, 20.5; IR (neat): 1710, 1608 cm^{-1}; MS (*m*/*z*): 250.1 (M^+); Anal. calc. for $C_{17}H_{14}O_2$: C, 81.58, H, 5.64 %; found: C, 81.50; H, 5.57%.

3-(2-Methoxybenzylidene)-6-methylchroman-2-one (3b)

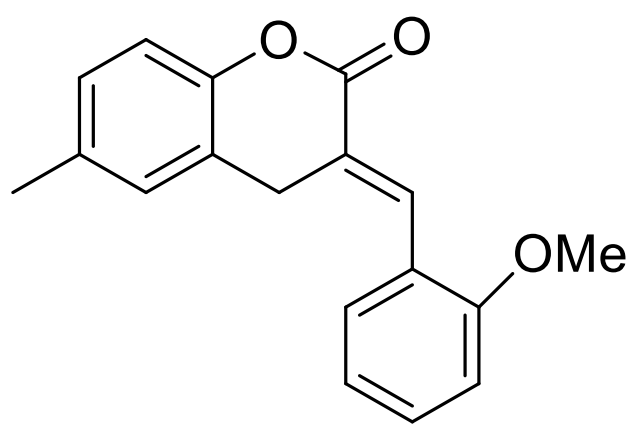

It was prepared following the similar procedure used for **3a** using **1a** and **2b** as MBH adduct. The product was recrystallized from chloroform-methanol mixture to furnish pure **3b** as colourless crystals.

Yield: 0.392 g (70%); mp: 143-145 °C; ^{1}H NMR (200 MHz, $CDCl_3$): δ 8.15 (t, *J* = 2.2 Hz, 1H), 7.49–7.34 (m, 2H), 7.12-6.96 (m, 5H), 3.95 (d, *J* = 2.0 Hz, 2H), 3.89 (s, 3H), 2.31 (s, 3H); ^{13}C NMR (50 MHz, $CDCl_3$): δ 164.5, 157.8, 148.2, 138.0, 133.6, 130.9, 129.9, 128.3, 128.2, 123.2, 122.8, 120.2, 119.9, 116.2, 110.6, 55.2, 28.6, 20.4; IR (neat): 1712, 1693 cm^{-1}; MS (*m*/*z*): 280.1 (M^+); Anal. calc. for $C_{18}H_{16}O_3$: C, 77.12; H, 5.75%; found: C, 77.36; H, 5.57%.

3-(2-Bromobenzylidene)-6-methylchroman-2-one (3c)

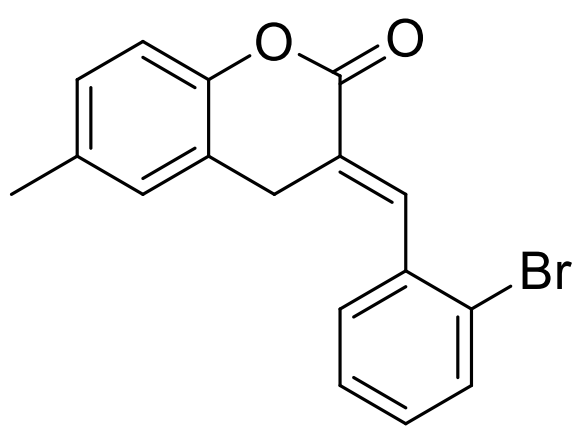

It was prepared using the similar procedure used for **3a** using **1a** and **2c** as MBH adduct. The product was recrystallized from chloroform-methanol mixture to furnish pure **3c** as colourless crystals.

Yield: 0.498 g (76%); mp: 99-101 °C; ^{1}H NMR (200 MHz, $CDCl_3$): δ 7.41–7.32 (m, 3H), 7.30–7.18 (m, 5H), 4.06 (d, , *J* = 1.2 Hz, 2H), 2.40 (s, 3H); ^{13}C NMR (50 MHz, $CDCl_3$): δ 164.8, 157.9, 148.4, 138.2, 133.7, 131.0, 130.0, 128.4, 128.3, 123.4, 122.9, 120.3, 120.0, 116.4, 110.7, 28.8, 20.5; IR (neat): 1708, 1596 cm^{-1}; MS (*m*/*z*): 328.1 (M^+); Anal. calc. for $C_{17}H_{13}BrO_2$: C, 62.03; H, 3.98 %; found: C, 62.15; H, 4.10%.

3-(4-Bromobenzylidene)-6-methylchroman-2-one (3d)

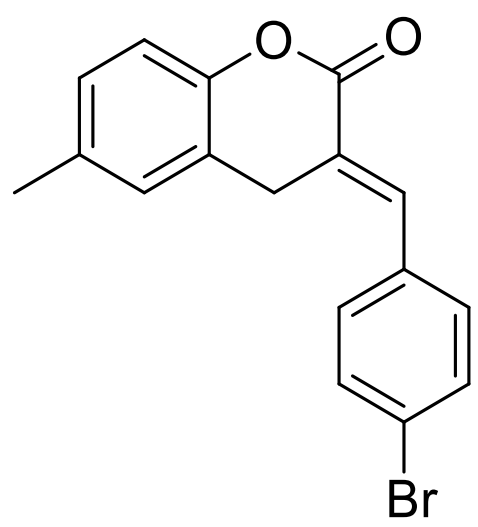

It was prepared using the similar procedure used for **3a** using **1a** and **2d** as MBH adduct. The product was recrystallized from chloroform-methanol mixture to furnish pure **3d** as colourless crystals.

Yield: 0.450 g (68%); mp: 183-185 °C; ^{1}H NMR (500 MHz, $CDCl_3$): δ 7.86 (s, 1H), 7.60 (d, *J* = 7 Hz, 2H), 7.30 (d, *J* = 7.5 Hz, 2H), 7.04 (d, *J* = 9 Hz, 1H), 6.96 (d, *J* = 9 Hz, 2H), 3.96 (s, 2H), 2.29 (s, 3H); ^{13}C NMR (125 MHz, $CDCl_3$): δ 164.2, 148.3, 140.9, 134.1, 133.4, 132.0, 131.9, 131.5, 128.8, 128.5, 123.9, 119.5, 116.7, 28.8, 20.6; IR (neat): 1707, 1599 cm^{-1}; MS (*m*/*z*): 328.0 (M^+); Anal. Calc. for $C_{17}H_{13}BrO_2$: C, 62.03; H, 3.98%; found: C, 62.08; H, 3.95%.

3-Benzylidene-6-chlorochroman-2-one (3e)

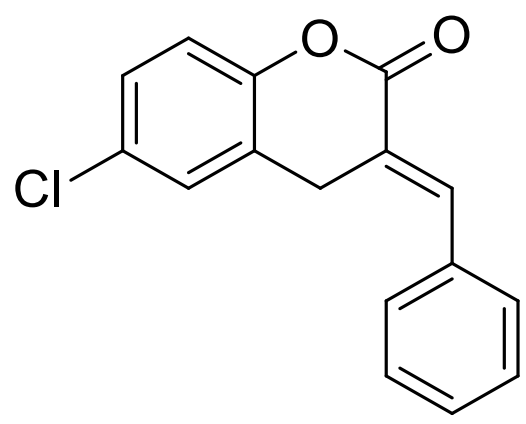

It was prepared using the similar procedure used for **3a** using **1b** and **2a** as MBH adduct. The product was recrystallized from chloroform-methanol mixture to furnish pure **3e** as colourless crystals.

Yield: 0.363 g (67%); mp: 132-134 ºC; ^{1}H NMR (500 MHz, $CDCl_3$): δ 7.99 (s, 1H), 7.50-7.43 (m, 5H), 7.21 (dd, *J* = 2 Hz, 8.5 Hz, 1H), 7.16(s, 1H), 7.02 (d, *J* = 8.5 Hz, 1H), 4.04 (s, 2H); ^{13}C NMR (125 MHz, $CDCl_3$): δ 163.8, 149.1, 143.4, 134.3, 130.1, 129.8, 129.5, 129.4, 128.8, 128.3, 127.9, 121.9, 118.4, 28.7; IR (neat): 1703, 1600 cm^{-1}; MS (*m*/*z*): 270.1 (M^+); Anal. calc. for $C_{16}H_{11}ClO_2$: C, 70.99; H, 4.10%; found: C, 71.13; H, 4.11%.

6-Chloro-3-(2-methoxybenzylidene)-chroman-2-one (3f)

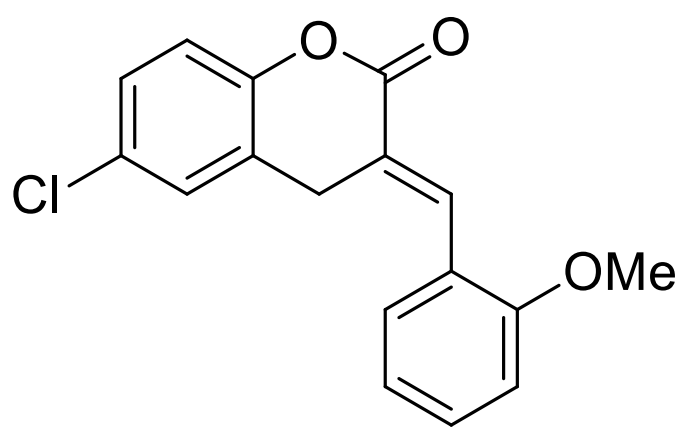

It was prepared using the similar procedure used for **3a** using **1b** and **2b** as MBH adduct. The product was recrystallized from chloroform-methanol mixture to furnish pure **3f** as colourless crystals.

Yield: 0.373g (62%); mp: 151-152 ºC; ^{1}H NMR (500 MHz, $CDCl_3$): δ 8.12 (s, 1H), 7.41 (t, J = 8 Hz, 1H), 7.28 (d, J = 7.5 Hz, 1H), 7.19 (dd, J = 3 Hz, 8.5 Hz, 1H), 7.10 (s, 1H), 7.04 (t, J = 7.5 Hz, 1H), 7.01 (d, J = 8.5 Hz, 1H), 6.96 (d, J = 8 Hz, 1H), 3.92 (s, 2H), 3.86 (s, 3H); ^{13}C NMR (125 MHz, $CDCl_3$): δ 164.1, 158.1, 149.3, 139.4, 131.3, 130.1, 129.3, 128.1, 127.8, 123.3, 122.6, 121.9, 120.2, 118.2, 110.9, 55.5, 28.8; IR (neat): 1720, 1595 cm^{-1}; MS (*m*/*z*): 300.1 (M^+); Anal. calc. for $C_{17}H_{13}ClO_3$: C, 67.89; H, 4.36 %; found: C, 67.90; H, 4.31%.

3-Benzylidene-6-bromochroman-2-one (3g)

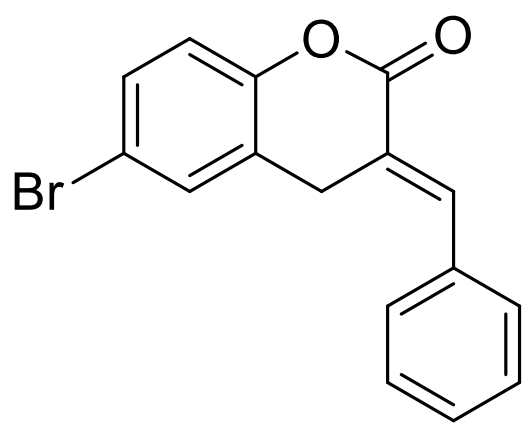

It was prepared using the similar procedure used for **3a** using **1c** and **2a** as MBH adduct. The product was recrystallized from chloroform-methanol mixture to furnish pure **3g** as colourless crystals.

Yield: 0.423 g (67%); mp: 162-163 °C; ^{1}H NMR (500 MHz, $CDCl_3$): δ 7.98 (s, 1H), 7.49-7.42 (m, 5H), 7.20 (dd, J = 2.5 Hz, 8.5 Hz, 1H), 7.15 (s, 1H), 7.01 (d, *J* = 8.5 Hz, 1H), 4.03 (s, 2H); ^{13}C NMR (125 MHz, $CDCl_3$): δ 163.7, 149.6, 143.3, 134.3, 131.2, 130.9, 130.1, 129.8, 128.8, 122.4, 121.9, 118.7, 116.9, 28.6; IR (neat): 1716, 1597 cm^{-1}; HRMS (ESI-TOF) m/z: $[M+H]^+$ Calcd. for $C_{16}H_{11}BrO_2$: 315.0020; Found: 314.9837.

6-Bromo-3-(2-methoxybenzylidene)-chroman-2-one (3h)

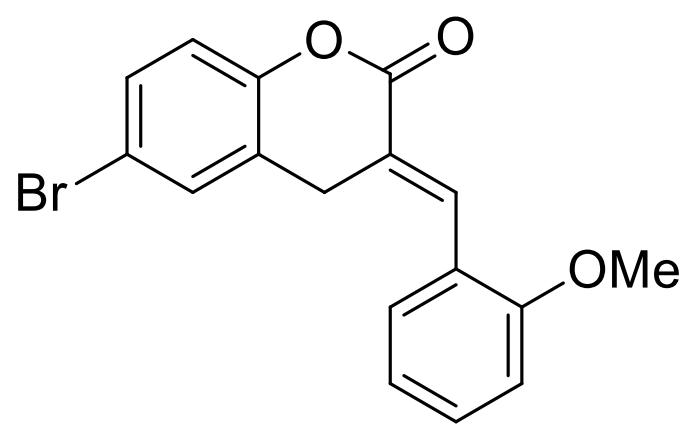

It was prepared using the similar procedure used for **3a** using **1c** and **2b** as MBH adduct. The product was recrystallized from chloroform-methanol mixture to furnish pure **3h** as colourless crystals.
Yield: 0.430 g (62 %); mp: 141-142 °C; ^{1}H NMR (500 MHz, $CDCl_3$): δ 8.12 (s, 1H), 7.42 (t, *J* = 8 Hz, 1H), 7.33 (dd, J = 2.5 Hz, 8.5 Hz, 1H), 7.28 (d, J = 7.5 Hz, 1H), 7.26 (s, 1H), 7.04 (t, J = 7.5 Hz, 1H), 6.96 (d, J = 8.5 Hz, 1H), 6.95 (d, J = 8.5 Hz, 1H), 3.93 (s, 2H), 3.86 (s, 3H); ^{13}C NMR (125 MHz, $CDCl_3$): δ 163.8, 158.1, 149.8, 139.3, 131.3, 131.0, 130.8, 130.1, 123.3, 123.1, 121.9, 120.2, 118.6, 116.7, 111.0, 55.5, 28.7; IR (neat): 1720, 1595 cm^{-1}; HRMS (ESI-TOF) m/z: $[M+H]^+$ Calcd. for $C_{17}H_{13}BrO_3$: 345.0126; Found: 345.0121.

Procedure for preparation of 2-benzylidene-1,2-dihydro-3*H*-benzo[f]chromen-3-one (6a) and 2-methylene-1-phenyl-1,2-dihydro-3*H*-benzo[f]chromen-3-one (6b)

A mixture of 2-naphthol (**5**, 2.0 mmol), MBH alcohol (**2a,** 2.2 mmol), and CSA (10 mol %) under solvent free conditions was heated at 100 °C for 2 h, when **5** was fully consumed (checked by TLC). The reaction mixture was quenched with saturated aqueous $NaHCO_3$ solution (1 mL), and the viscous mass thus obtained was dissolved in EtOAc. The organic layer was washed with H_2O, brine, and dried (Na_2SO_4). The solvent was removed under *vacuo*, and the residue was purified by column chromatography (silica gel, EtOAc-petroleum ether) to afford pure **6a** as colourless solid and pure **6b** as pale-yellow thick liquid.

2-Benzylidene-1,2-dihydro-3*H*-benzo[f]chromen-3-one (6a)

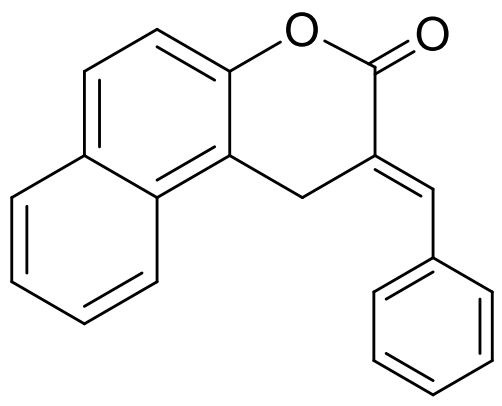

Yield: 0.250 g (44%); mp: 137-139 °C; ^{1}H NMR (500 MHz, $CDCl_3$): δ 8.13 (s, 1H), 7.85 (t, *J* = 8Hz, 2H), 7.78 (d, *J* = 9 Hz, 1H), 7.60-7.53 (m, 5H), 7.47 (t, *J* = 7.5 Hz, 2H), 7.28 (s, 1H), 4.41 (d, *J* = 2.5 Hz, 2H); ^{13}C NMR (125 MHz, $CDCl_3$): δ 163.8, 147.9, 143.4, 134.8, 131.0, 130.8, 130.3, 129.7, 129.1, 128.9, 128.8, 127.2, 125.1, 122.3, 122.2, 117.5, 112.5, 26.1; IR (neat): 1720, 1633, 1602 cm^{-1}; HRMS (ESI-TOF) m/z: $[M+H]^+$ Calcd. for $C_{20}H_{14}O_2$: 287.1072; Found: 287.1059.

2-Methylene-1-phenyl-1,2-dihydro-3*H*-benzo[f]chromen-3-one (6b)

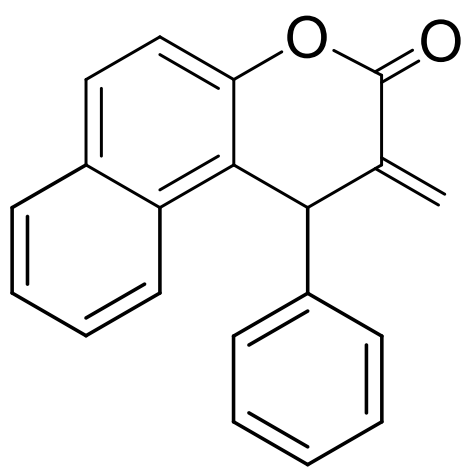

Yield: 0.195 g (34%); ^{1}H NMR (500 MHz, $CDCl_3$): δ 7.89 (d, *J* = 9.0 Hz, 2H), 7.84 (d, *J* = 8.0 Hz, 1H), 7.51-7.44 (m, 2H), 7.36 (d, *J* = 9.0 Hz, 1H), 7.28-7.26 (m, 2H), 7.22-7.19 (m, 1H), 7.16 (d, *J* = 7.5 Hz, 2H), 6.46 (s, 1H), 6.04 (s, 1H), 5.56 (s, 1H); ^{13}C NMR (125 MHz, $CDCl_3$): δ 162.6, 148.6, 140.3, 136.7, 131.1, 130.8, 129.9, 129.4, 129.1, 128.8, 128.7, 127.4, 126.7, 125.2, 122.9, 117.5, 117.3, 45.4; IR (neat): 1747, 1712, 1625 cm^{-1}; HRMS (ESI-TOF) m/z: $[M+H]^+$ Calcd. for $C_{20}H_{14}O_2$: 287.1072; Found: 287.1056.

Typical procedure for preparation of 3-hydroxy-6-methyl-2*H*-chroman-2-one (8a)

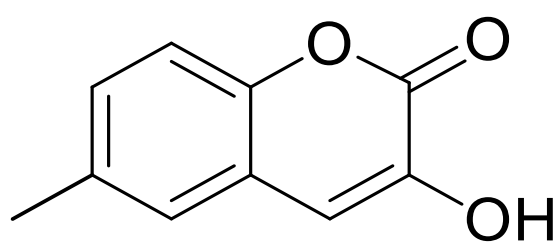

To a stirred solution of 3-benzylidene-6-methylchroman-2-one (**3a**, 1.0 mmol) in dry CH_2Cl_2 at -78 ^{0}C, O_3/O_2 mixture was bubbled for 30 min (0.03125 mole of O_3). To remove the excess amount of O_3, the reaction mixture was purged with O_2 for 5 minutes. Triphenyl phosphine (4 mmol) was then added to the reaction mixture and stirred overnight at ambient temperature. TLC of the reaction mixture showed complete consumption of **3a**. The viscous mass thus obtained was dissolved in EtOAc, washed with H_2O, brine, and dried (Na_2SO_4). The solvent was removed under *vacuo*, and the residue was purified by column chromatography (silica gel, EtOAc-petroleum ether) to afford the desired product **8a** as colourless solid.
Yield: 0.160 g (91%); mp: 194-195 ^oC; ^{1}H NMR (500 MHz, $CDCl_3$): δ 7.24–7.19 (m, 3H), 7.00 (s, 1H), 6.17 (s, 1H), 2.40 (s, 3H); ^{13}C NMR (125 MHz, $CDCl_3$): δ 160.7, 147.5, 140.2, 134.8, 129.4, 126.5, 120.1, 116.1, 114.4, 20.8; IR (neat): 3371, 1672, 1645, 1614 cm^{-1}; MS (*m*/*z*): 176.1 (M^+); Anal. calc. for $C_{10}H_8O_3$: C, 68.18; H, 4.58%; found: C, 68.01; H, 4.63%.

6-Chloro-3-hydroxy-2*H*-chromene-2-one (8b)

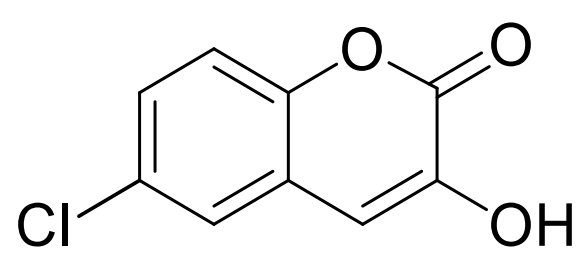

It was prepared using the similar procedure used for **8a** using **3e** as starting material. The product was recrystallized from chloroform-methanol mixture to furnish pure **8b** as colourless crystals.
Yield: 0.140 g (71%); mp: 249-251 ^oC; ^{1}H NMR (500 MHz, $(CD_3)_2CO$): δ 7.60 (d, *J* = 2.5 Hz, 1H), 7.41 (dd, *J* = 2.5 Hz, 8.5 Hz, 1H), 7.33 (d, *J* = 8.5 Hz, 1H), 7.14 (s, 1H), 3.38 (br s, 1H); ^{13}C NMR (125 MHz, $(CD_3)_2CO$): δ 159.3, 149.1, 143.3, 130.2, 128.4, 126.6, 123.5, 118.5, 114.2; IR (neat): 3352, 1687, 1651, 1622 cm^{-1}; HRMS (ESI-TOF) m/z: $[M-1]^+$ Calcd. for $C_9H_5ClO_3$: 194.9849; Found: 194.9852.

6-Bromo-3-hydroxy-2*H*-chromene-2-one (8c)

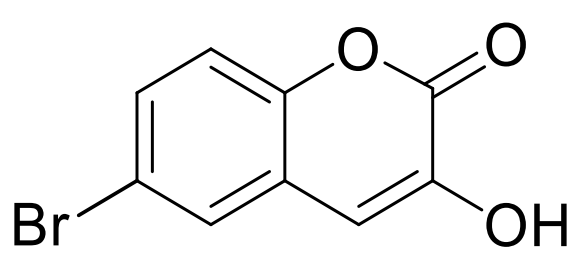

It was prepared using the similar procedure used for **8a** using **3g** as starting material. The product was recrystallized from chloroform-methanol mixture to furnish pure **8c** as colourless crystals.

Yield: 0.185 g (77%); mp: 260-262 ºC; ^{1}H NMR (500 MHz, $(CD_3)_2CO$): δ 7.75 (d, *J* = 2.5 Hz, 1H), 7.53 (dd, *J* = 2.5 Hz, 9 Hz, 1H), 7.28 (d, *J* = 9 Hz, 1H), 7.13 (s, 1H), 3.02 (br s, 1H); ^{13}C NMR (125 MHz, $(CD_3)_2CO$): δ 159.2, 149.6, 143.3, 131.2, 129.6, 124.0, 118.8, 117.6, 114.0; IR (neat): 3352, 1687, 1645, 1614 cm^{-1}; HRMS (ESI-TOF) m/z: $[M+H]^+$ Calcd. for $C_9H_5BrO_3$: 240.9500; Found: 240.9332.

2-Hydroxy-1-phenyl-3*H*-benzo[*f*]chromene-3-one (9)

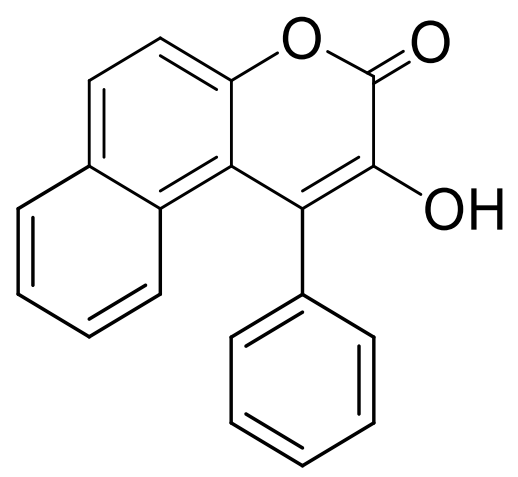

It was prepared using the similar procedure used for **8a** using **6b** as starting material. The product was recrystallized from chloroform-methanol mixture to furnish pure **9** as colourless solid.

Yield: 0.147 g (51%); mp: 234-235 ºC; ^{1}H NMR (500 MHz, $CDCl_3$): δ 7.89 (d, *J* = 9.0 Hz, 1H), 7.84 (d, *J* = 8 Hz, 1H), 7.57-7.53 (m, 4H), 7.42-7.38 (m, 3H), 7.24 (d, *J* = 8.5 Hz, 1H), 7.11 (t, *J* = 8 Hz, 1H), 6.30 (s, 1H); ^{13}C NMR (125 MHz, $CDCl_3$): δ 159.8, 148.4, 138.0, 134.8, 131.9, 130.5, 129.6, 129.2, 128.9, 128.8, 128.6, 128.4, 126.3, 125.7, 125.4, 117.0, 114.7; IR (neat): 3368, 1670, 1645, 1614 cm^{-1}; MS (*m*/*z*): 288.2 (M^+); Anal. calc. for $C_{19}H_{12}O_3$: C, 79.16; H, 4.20%; found: C, 78.79; H, 4.59%.

3. Molecular Electrostatic Potential (MEP) studies.

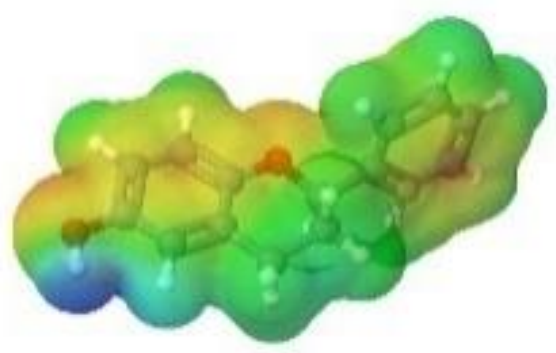	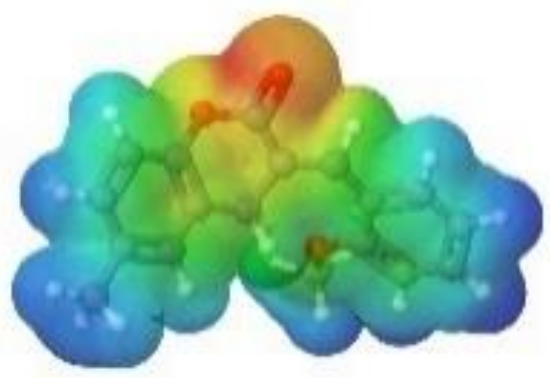	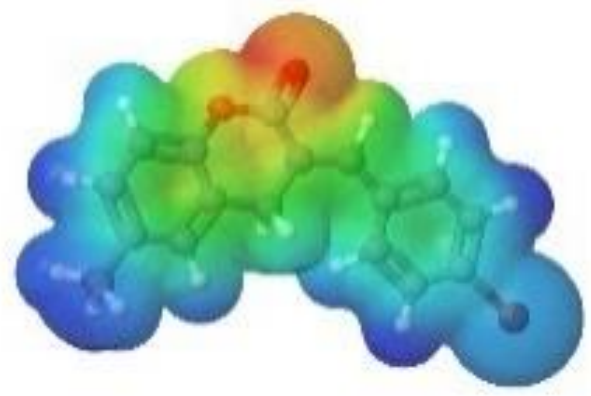
**3a**	**3b**	**3c**
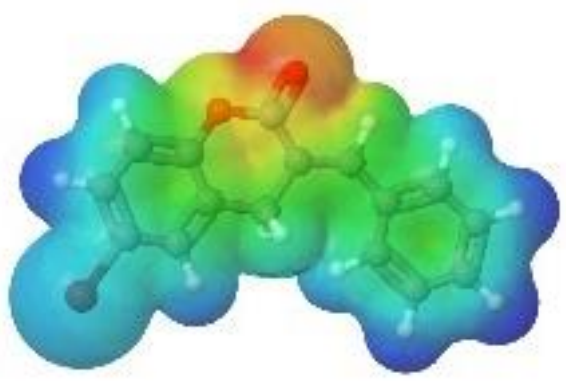	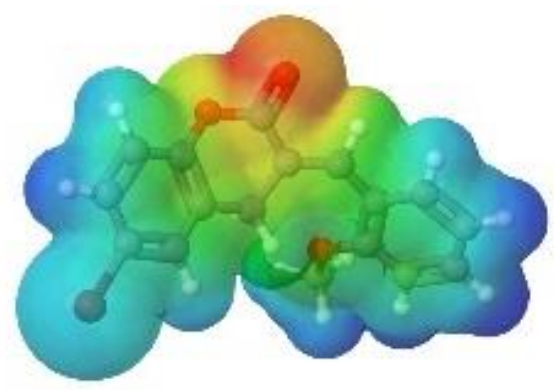	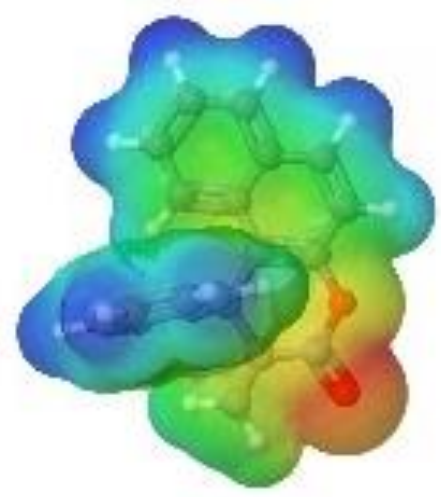
**3g**	**3h**	**6b**
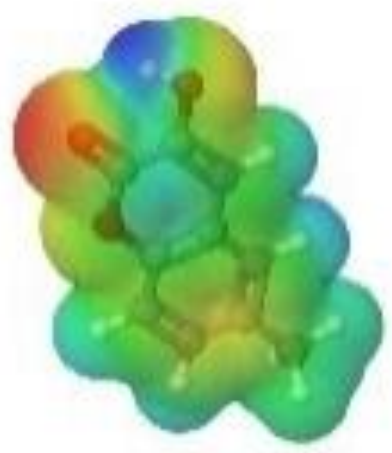	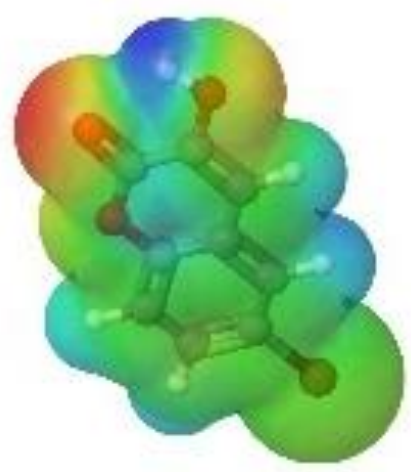	
**8a**	**8c**	

Figure S1. Molecular Electrostatic Potential (MEP) of compounds **3a-c**, **3g-h**, **4a**, **6b**, **8a** and **8c**.

4. Docking Study and Molecular Dynamics Simulation Study.

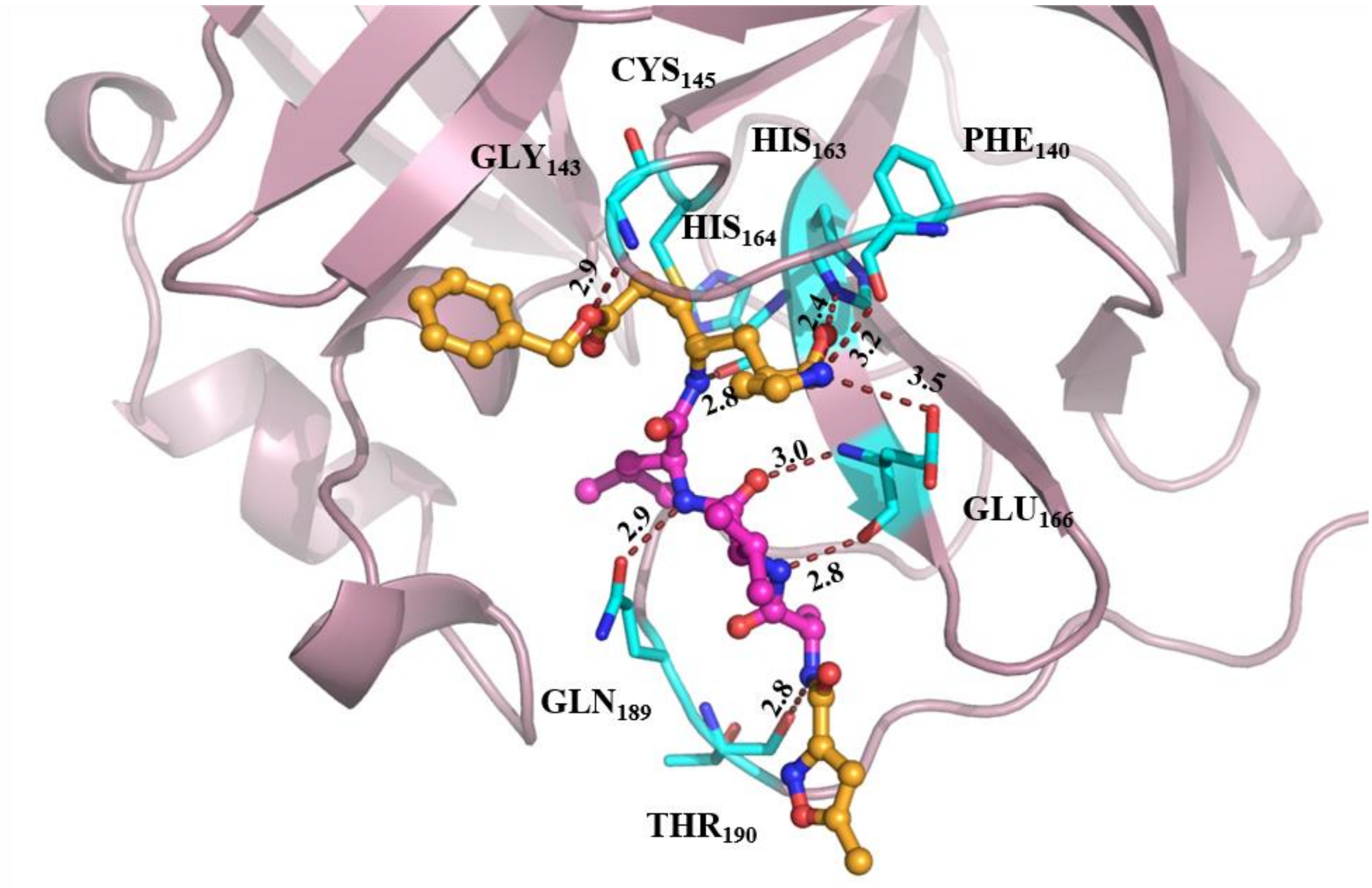

Figure S2: The interaction between 3CLpro and inhibitor N3. The enzyme is in cartoon representation and compound in ball-stick representation. The interacting residues were shown in the stick from with their distances in dashes. (Ref 10 in manuscript)

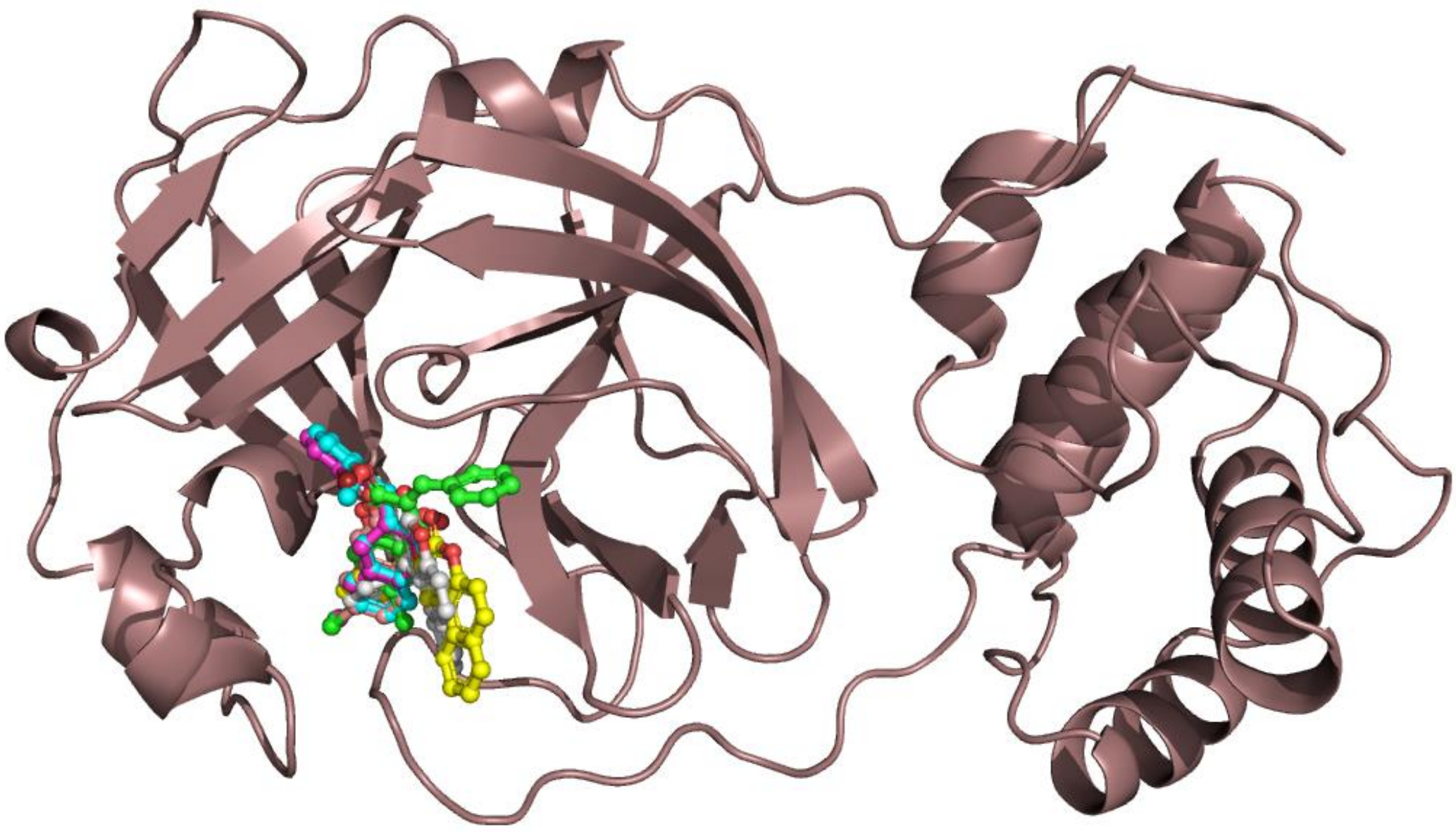

Figure S3: The docked structure of compounds **3d-f, 6a, 8b,** and **9** with 3CLpro enzyme. Representation of enzyme is shown in cartoon and compounds in ball-stick format.

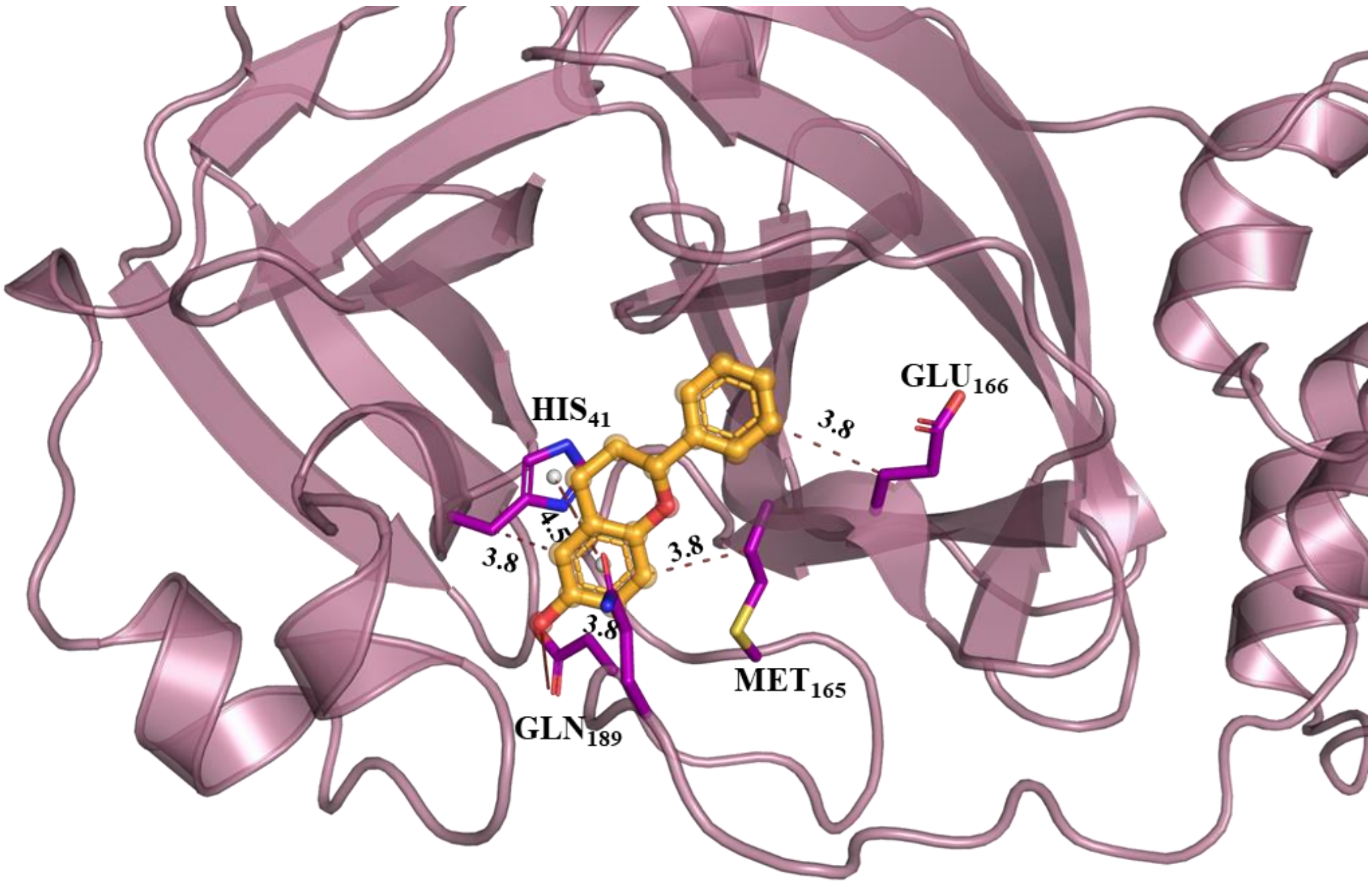

Figure S4. The interaction between 3CLpro enzyme (PDB 6LU7) and the compound **3a**. The enzyme is in cartoon representation and compound in ball-stick representation. The interacting residues were shown in the stick form with their distances (shown in dashes).

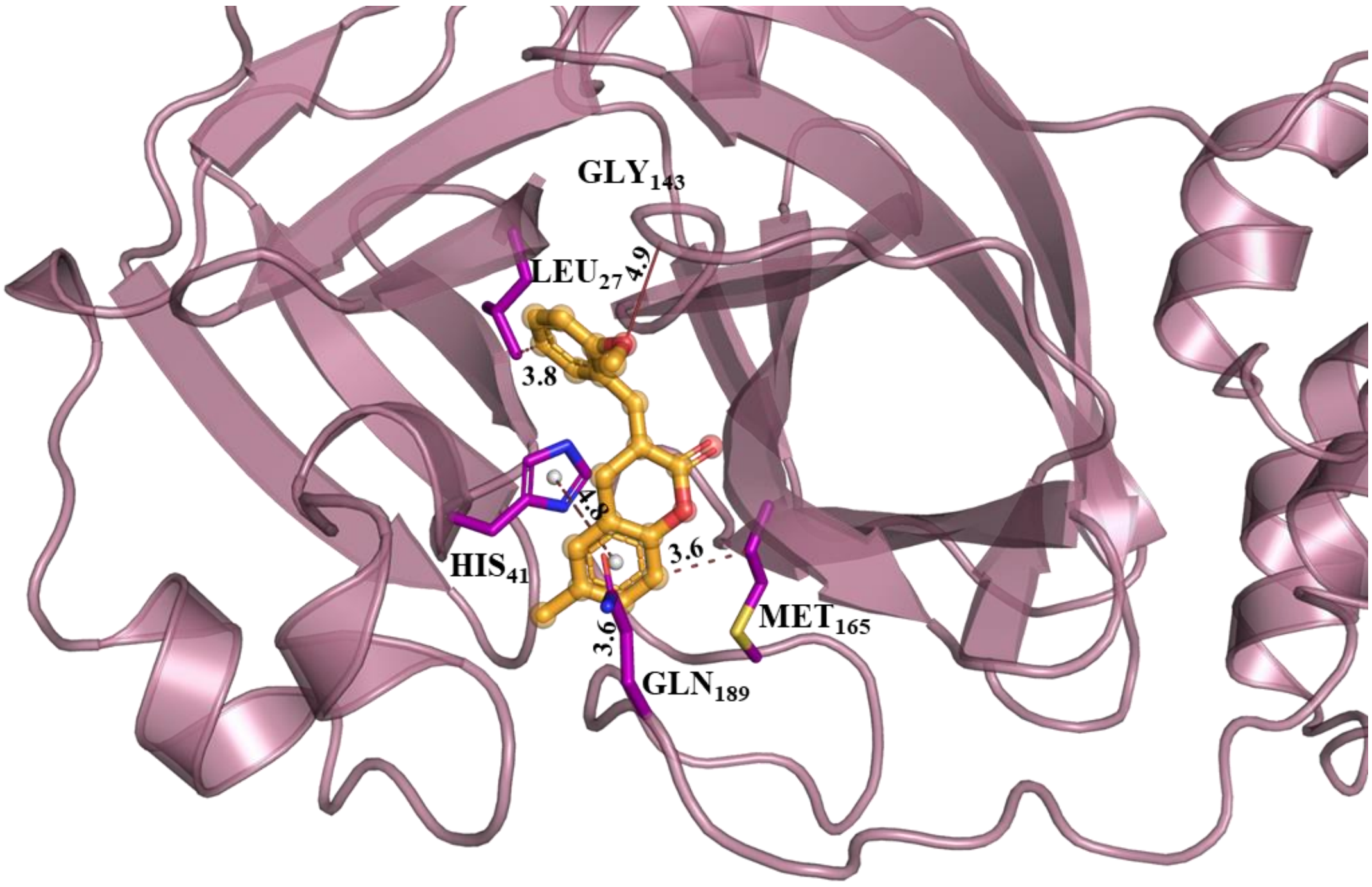

Figure S5. The interaction between 3CLpro enzyme (PDB 6LU7) and the compound **3b**. The enzyme is in cartoon representation and compound in ball-stick representation. The interacting residues were shown in the stick form with their distances (shown in dashes).

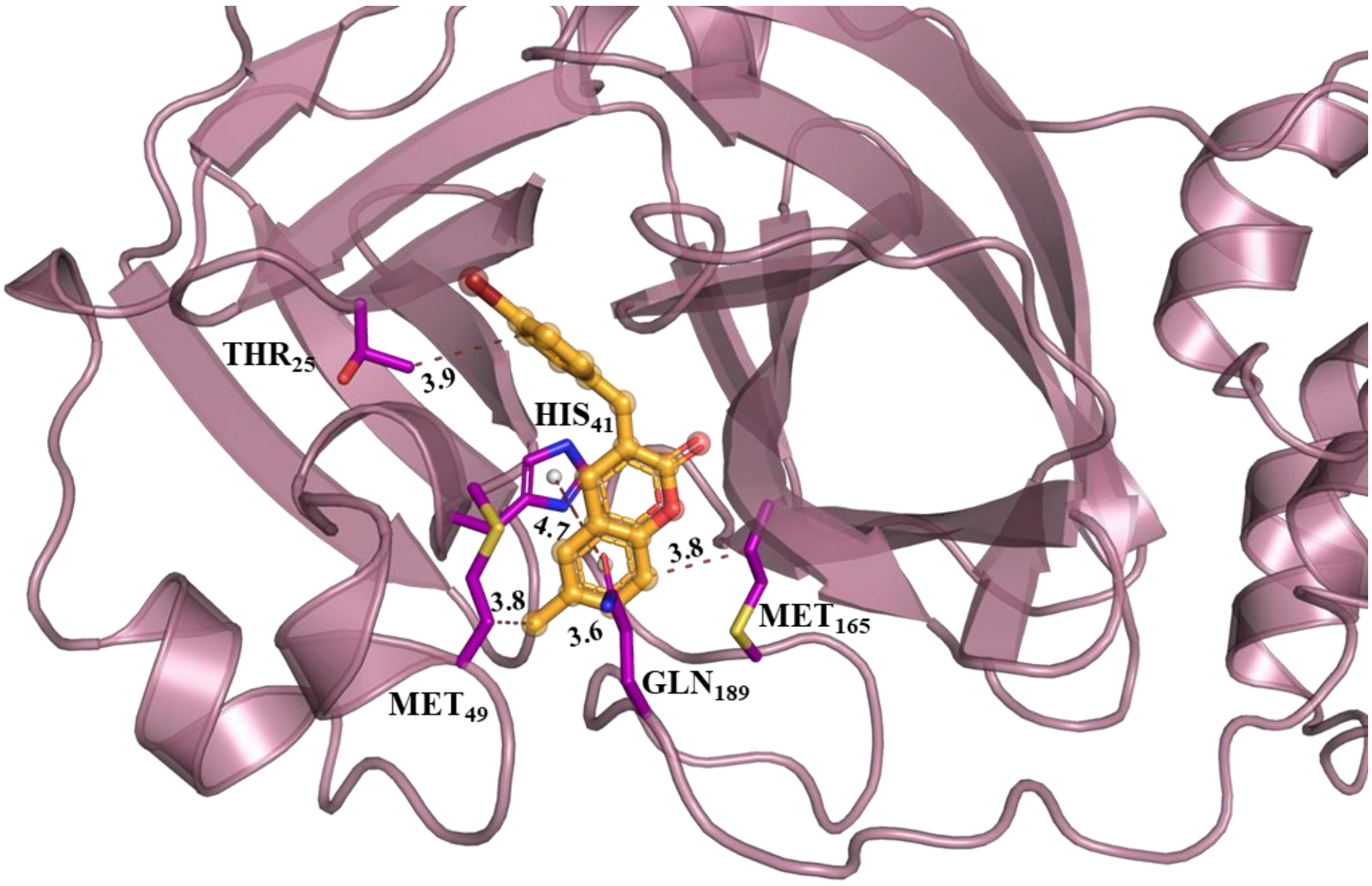

Figure S6. The interaction between 3CLpro enzyme (PDB 6LU7) and the compound **3c**. The enzyme is in cartoon representation and compound in ball-stick representation. The interacting residues were shown in the stick form with their distances (shown in dashes).

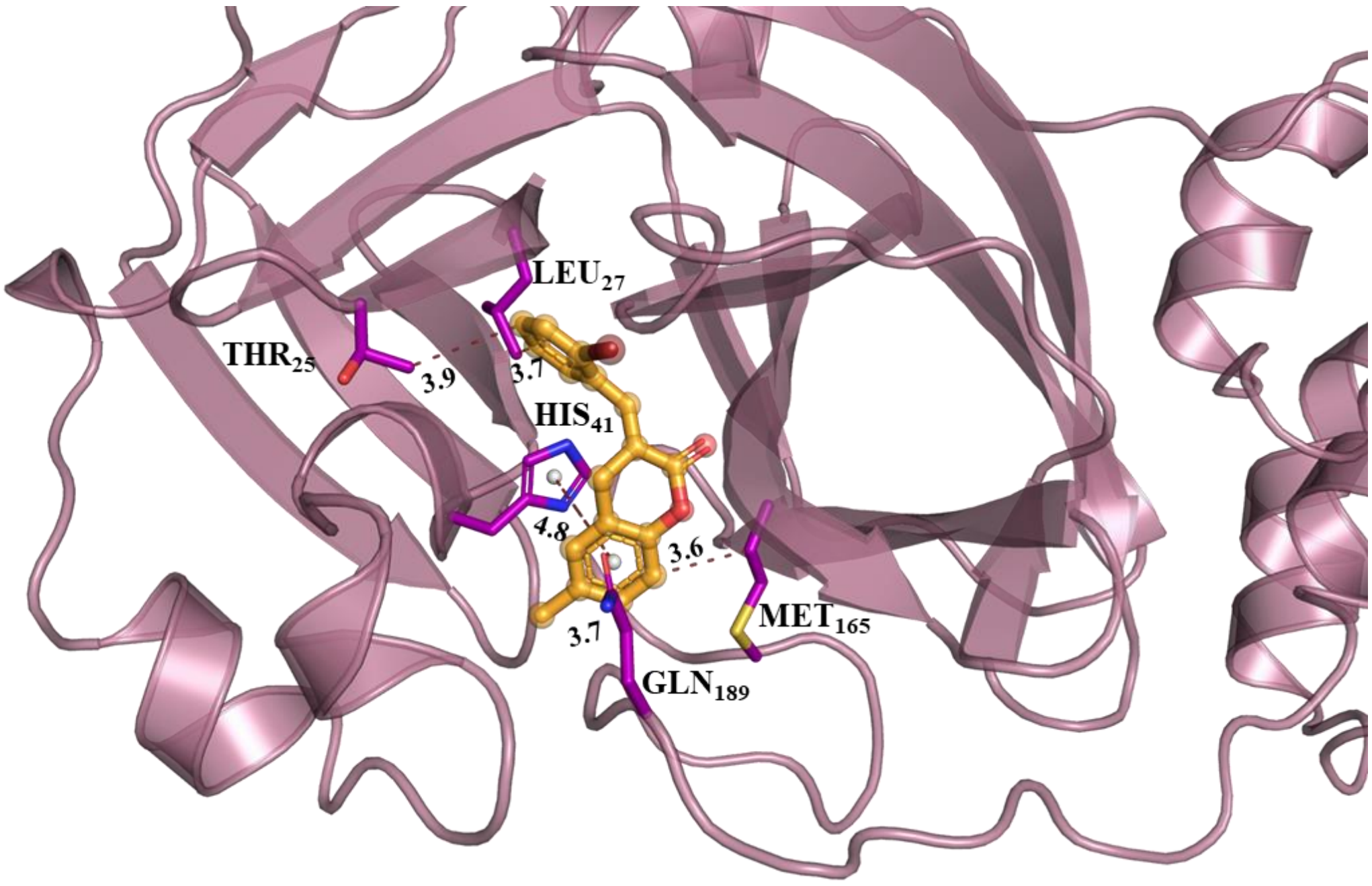

Figure S7. The interaction between 3CLpro enzyme (PDB 6LU7) and the compound **3d**. The enzyme is in cartoon representation and compound in ball-stick representation. The interacting residues were shown in the stick form with their distances (shown in dashes).

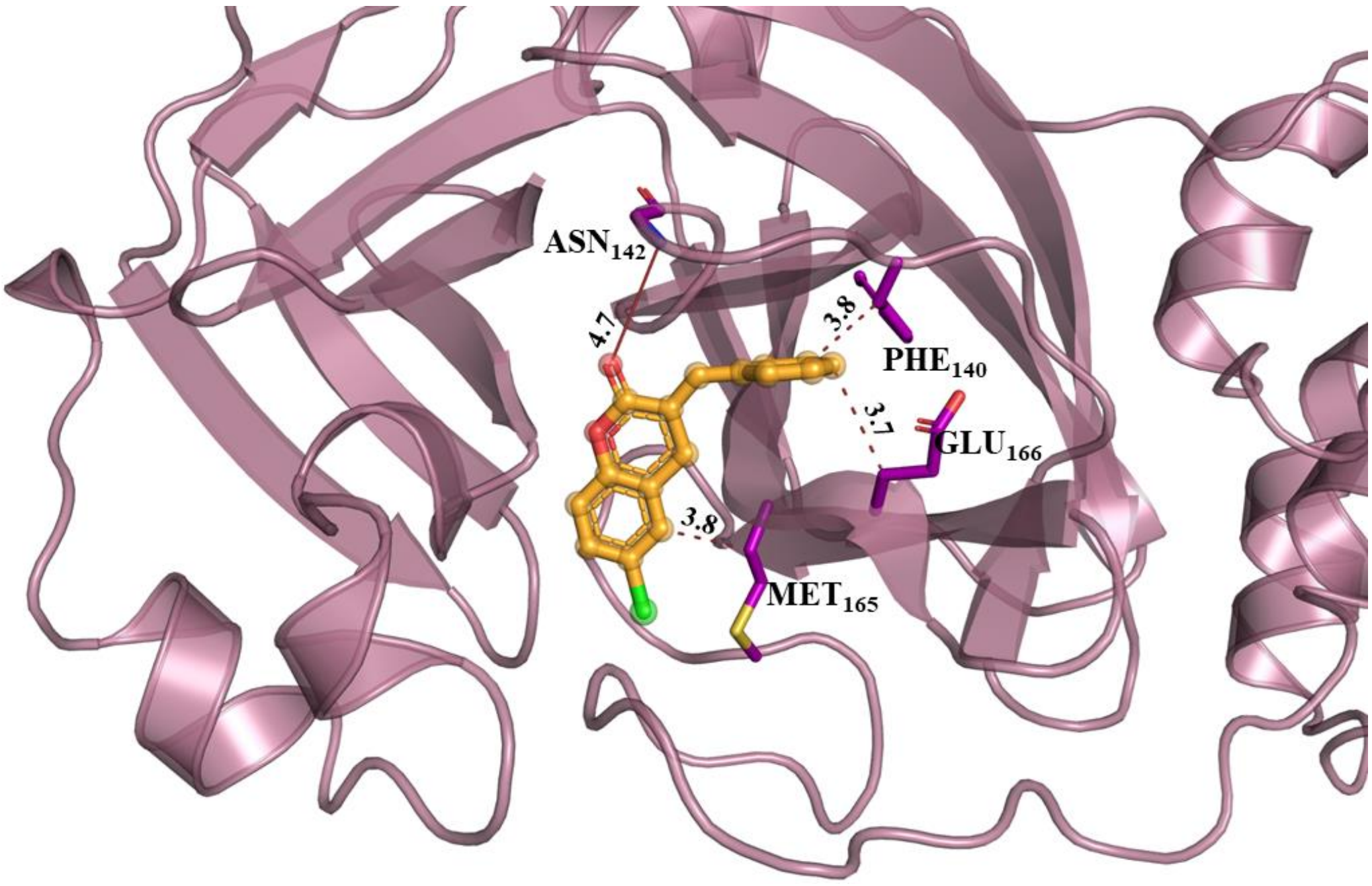

Figure S8. The interaction between 3CLpro enzyme (PDB 6LU7) and the compound **3e**. The enzyme is in cartoon representation and compound in ball-stick representation. The interacting residues were shown in the stick form with their distances (shown in dashes).

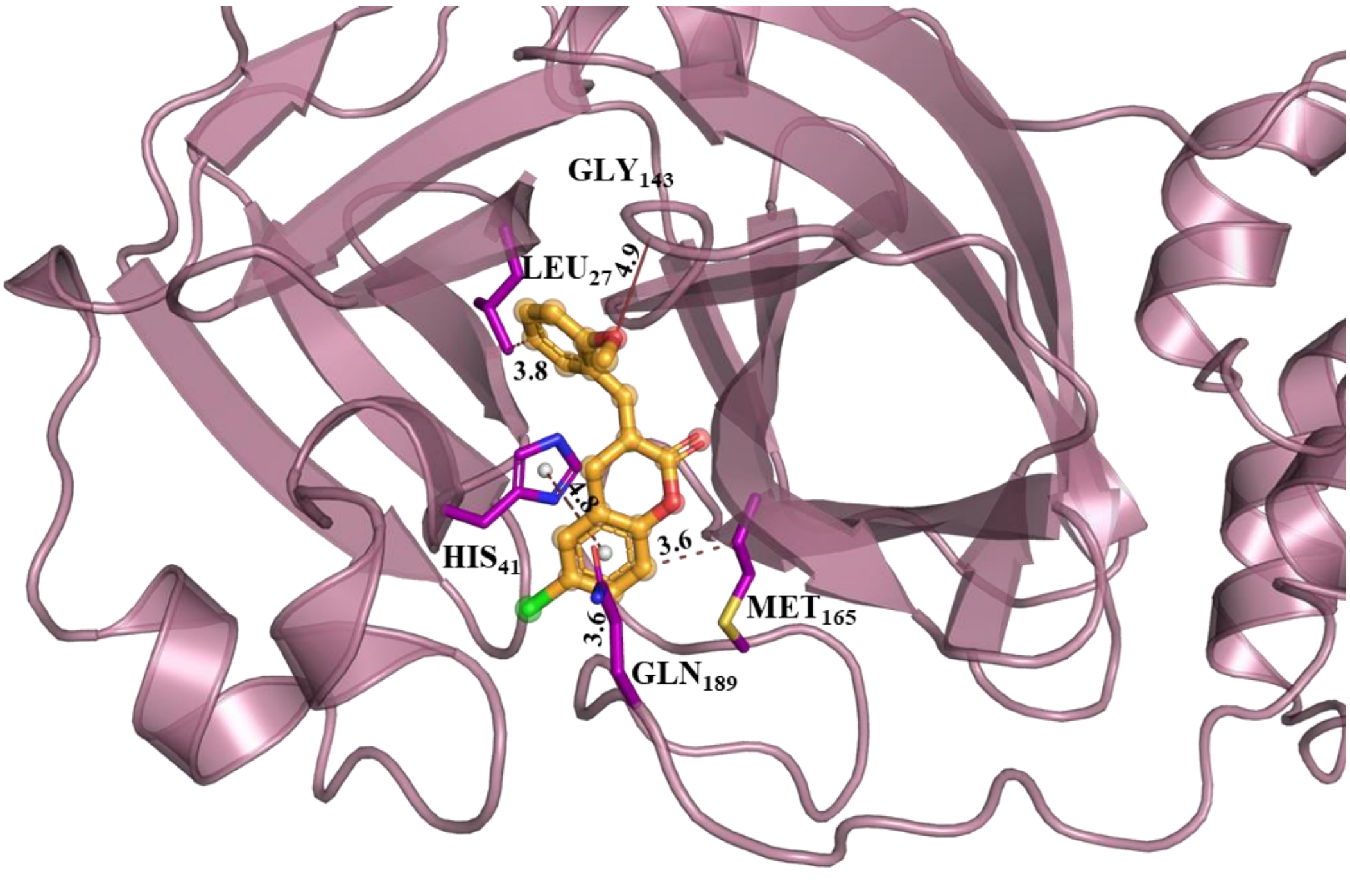

Figure S9. The interaction between 3CLpro enzyme (PDB 6LU7) and the compound **3f**. The enzyme is in cartoon representation and compound in ball-stick representation. The interacting residues were shown in the stick form with their distances (shown in dashes).

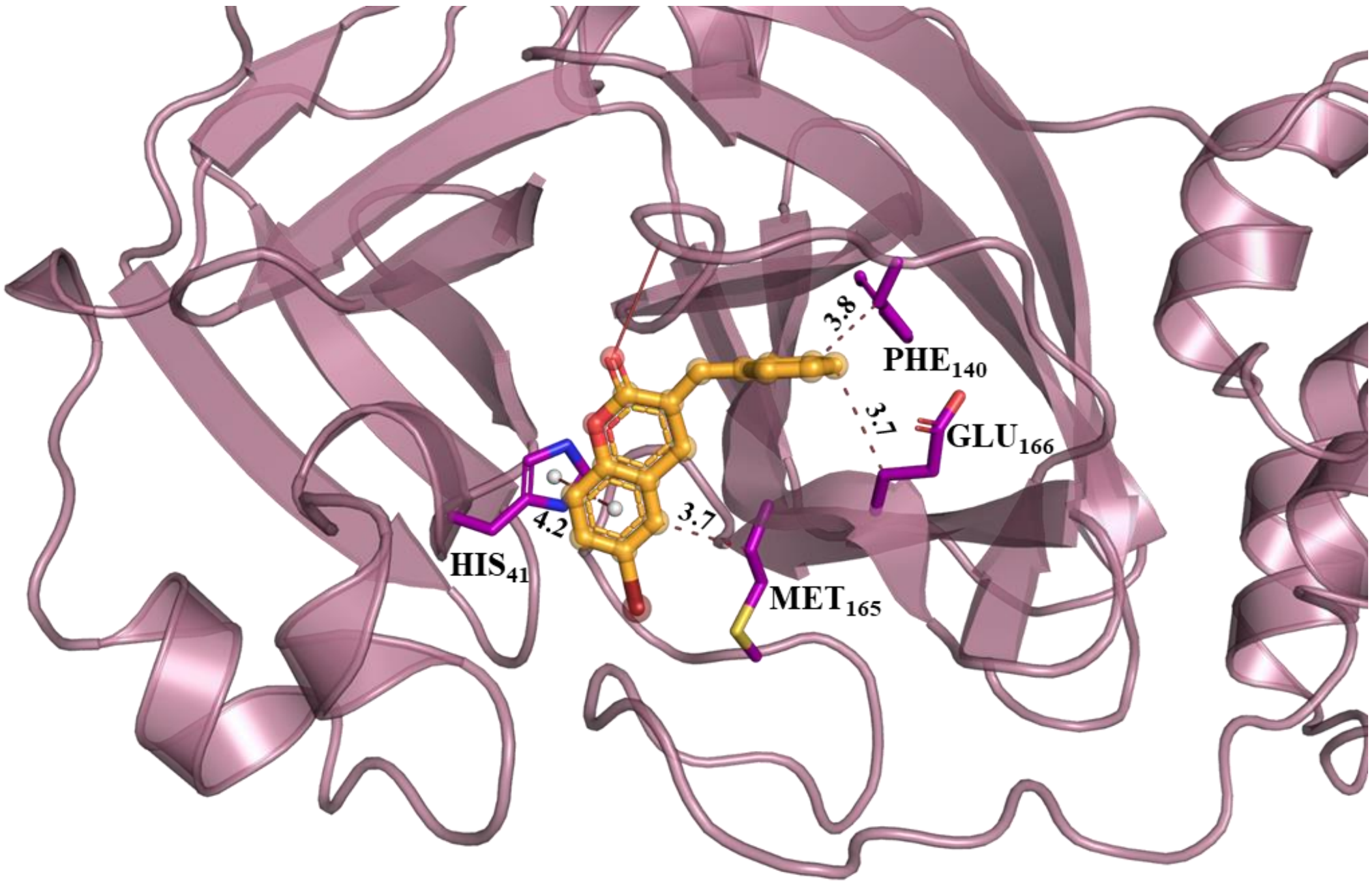

Figure S10. The interaction between 3CLpro enzyme (PDB 6LU7) and the compound **3g**. The enzyme is in cartoon representation and compound in ball-stick representation. The interacting residues were shown in the stick form with their distances (shown in dashes).

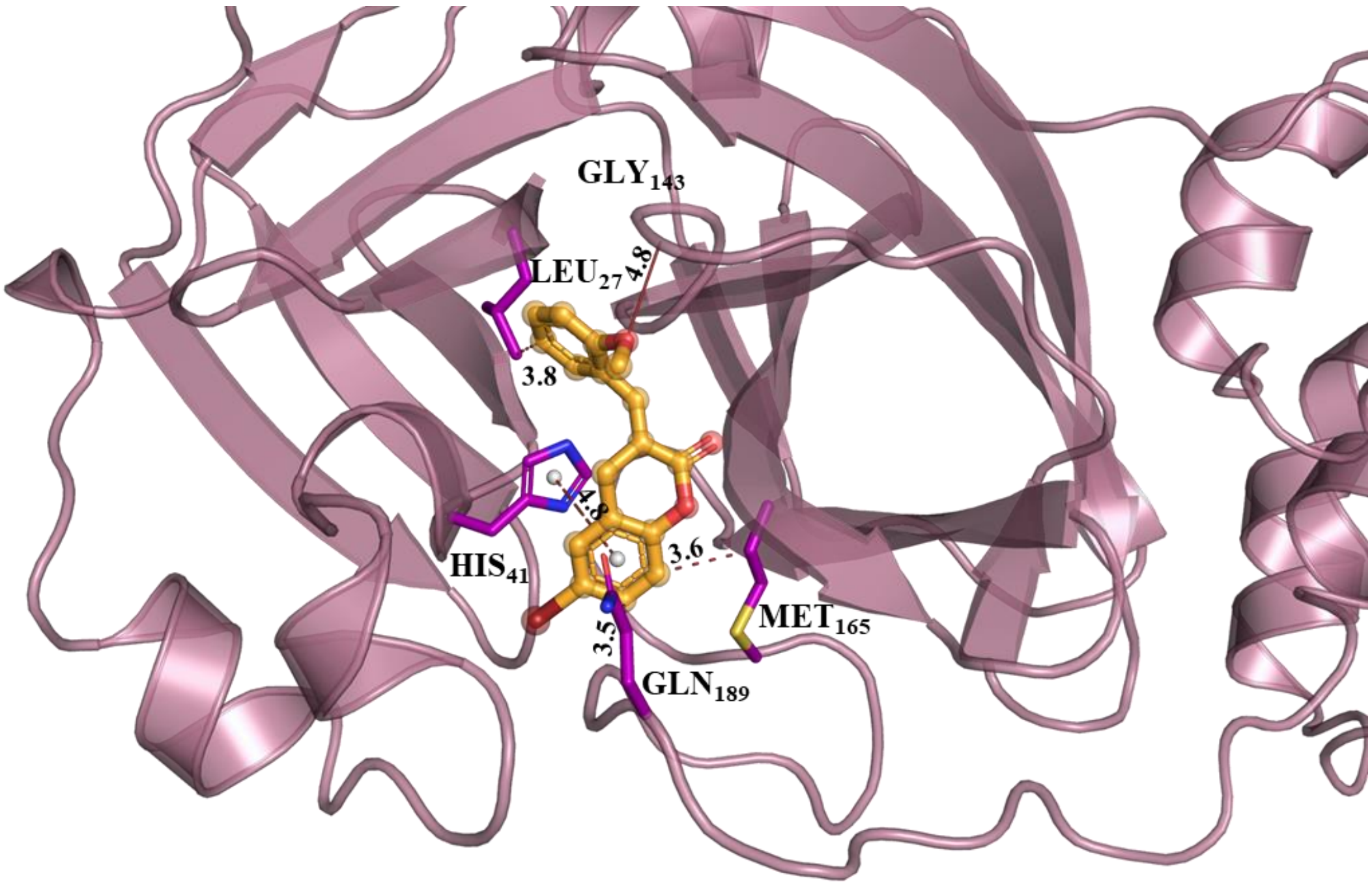

Figure S11. The interaction between 3CLpro enzyme (PDB 6LU7) and the compound **3h**. The enzyme is in cartoon representation and compound in ball-stick representation. The interacting residues were shown in the stick form with their distances (shown in dashes).

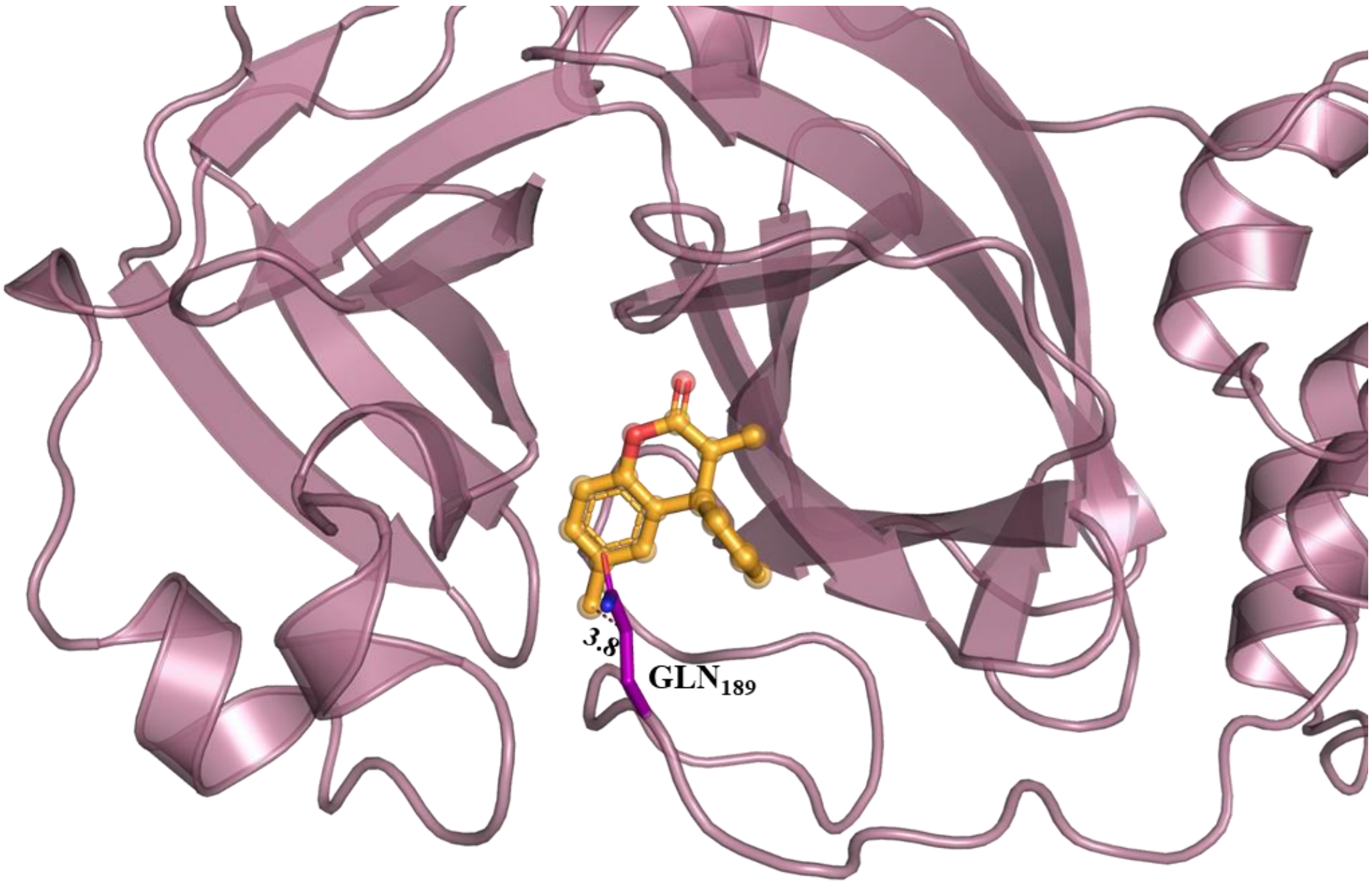

Figure S12. The interaction between 3CLpro enzyme (PDB 6LU7) and the compound **4a**. The enzyme is in cartoon representation and compound in ball-stick representation. The interacting residues were shown in the stick form with their distances (shown in dashes).

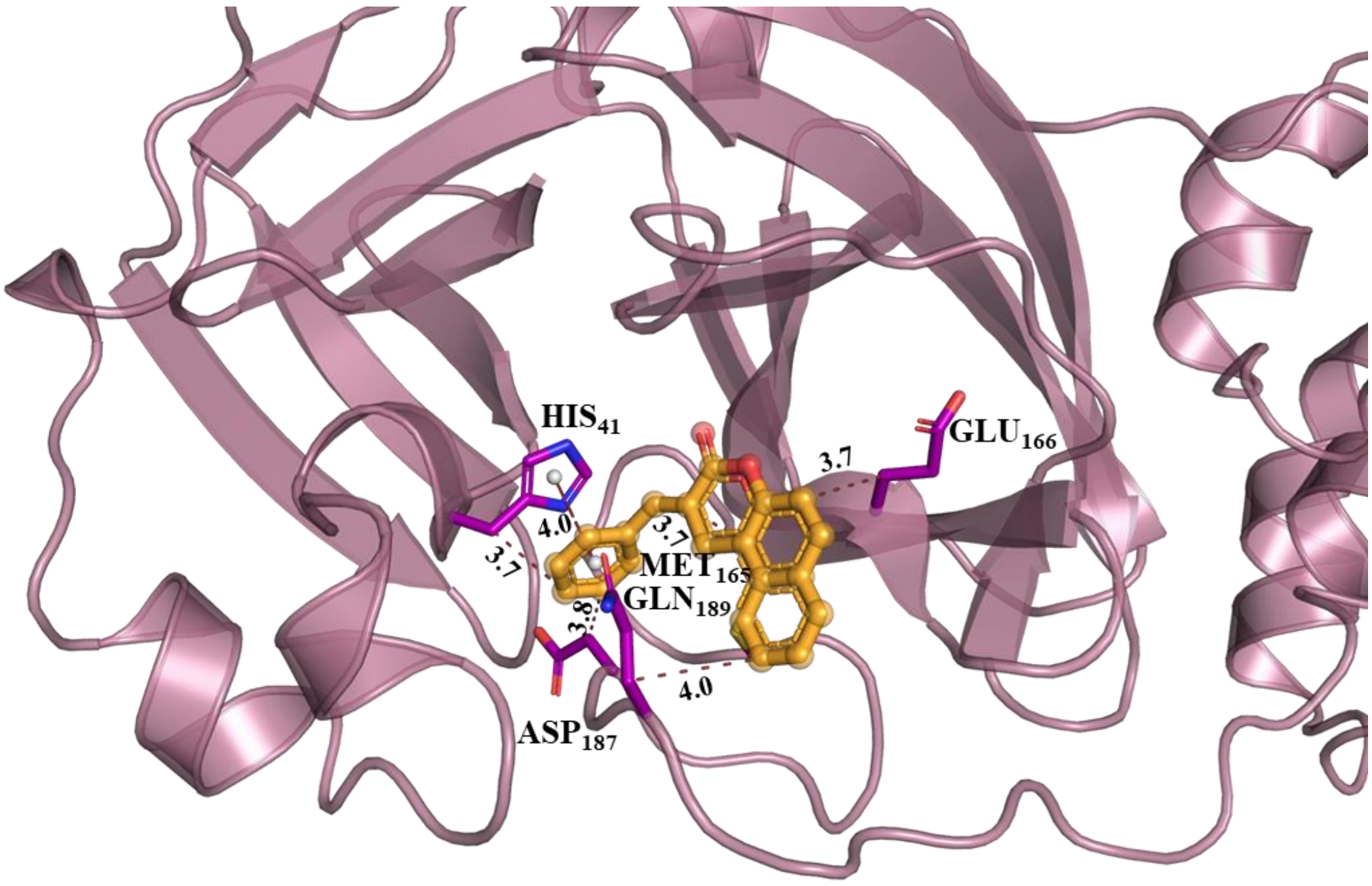

Figure S13. The interaction between 3CLpro enzyme (PDB 6LU7) and the compound **6a**. The enzyme is in cartoon representation and compound in ball-stick representation. The interacting residues were shown in the stick form with their distances (shown in dashes).

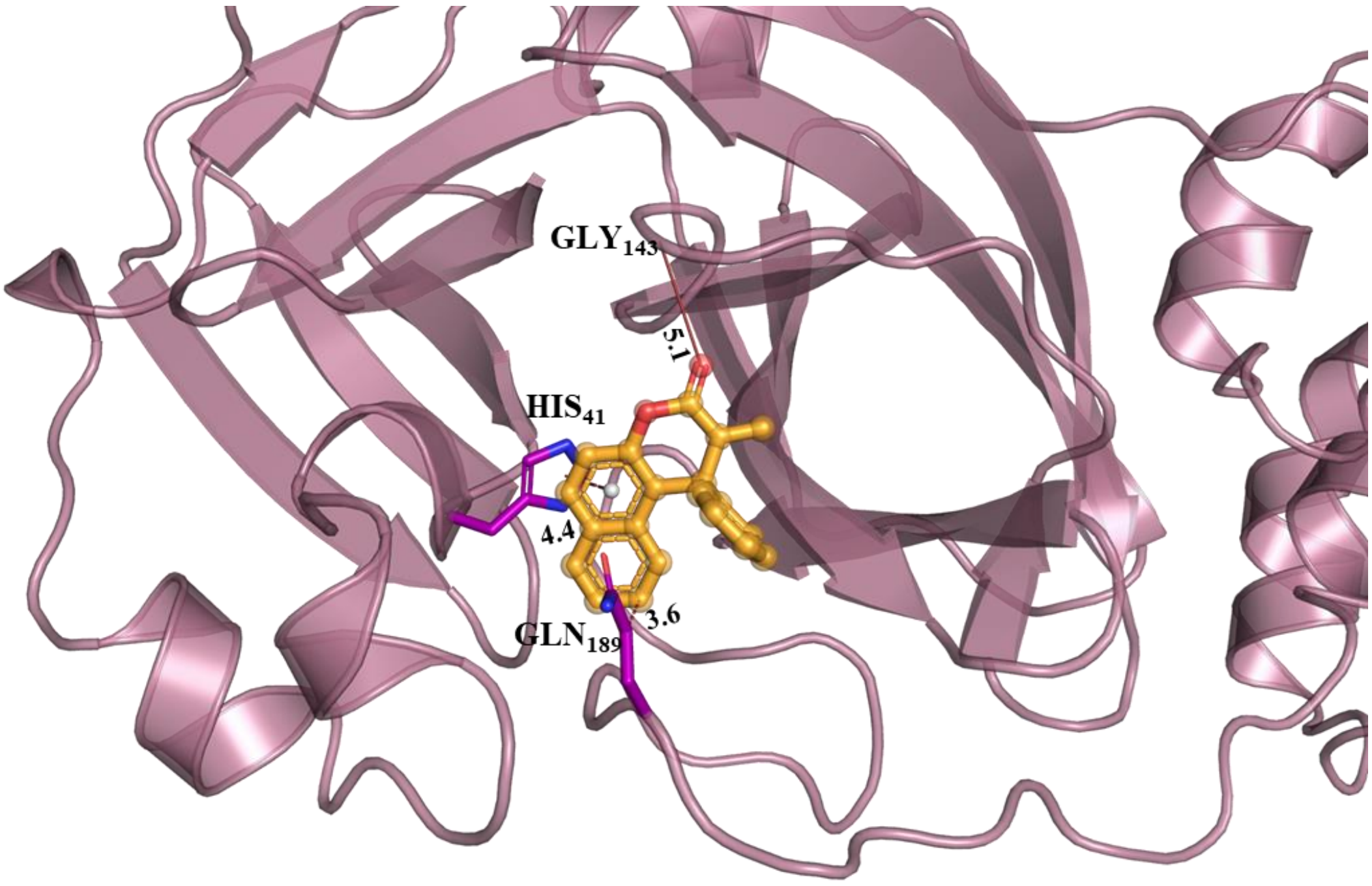

Figure S14. The interaction between 3CLpro enzyme (PDB 6LU7) and the compound **6b**. The enzyme is in cartoon representation and compound in ball-stick representation. The interacting residues were shown in the stick form with their distances (shown in dashes).

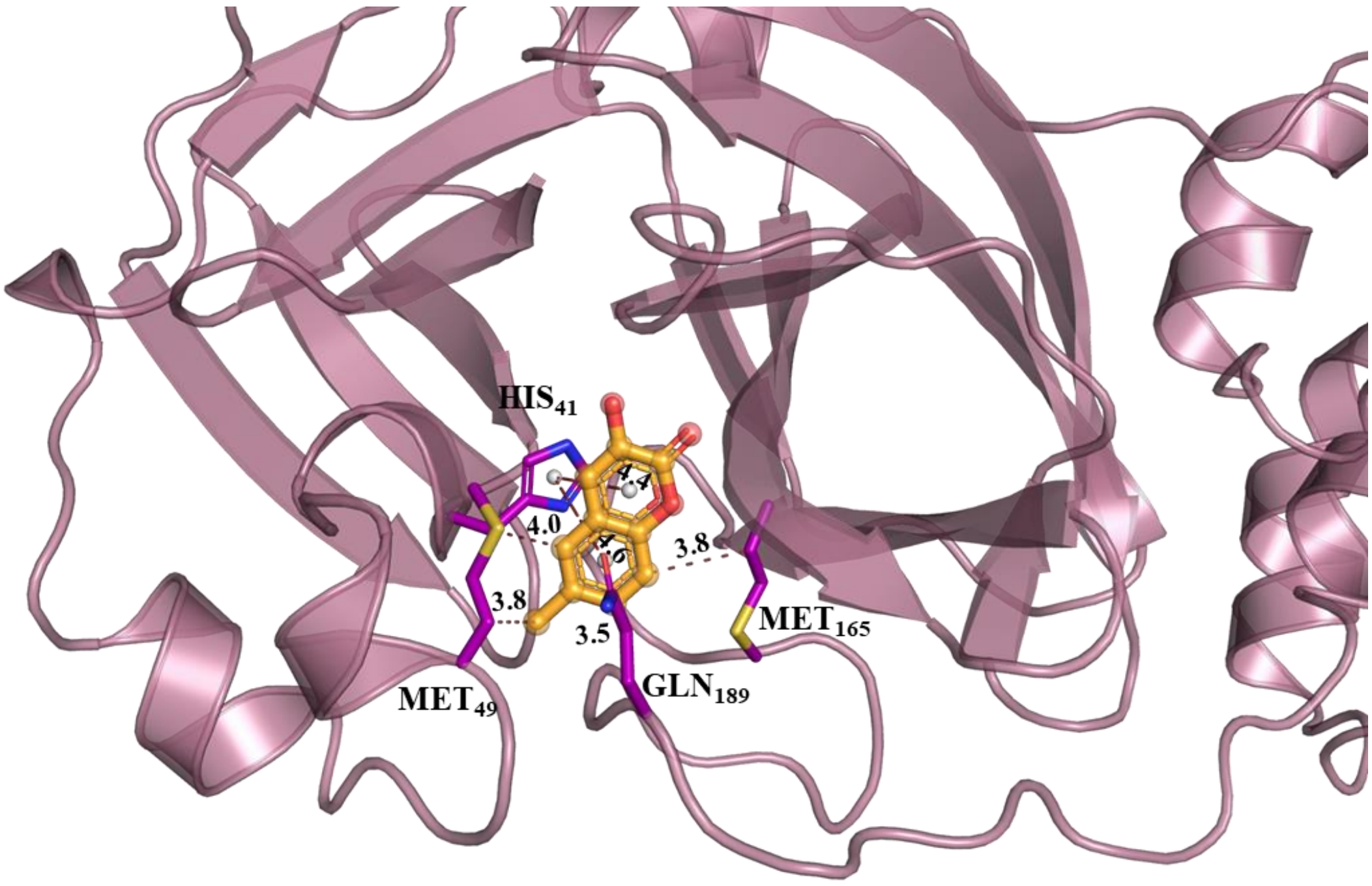

Figure S15. The interaction between 3CLpro enzyme (PDB 6LU7) and the compound **8a**. The enzyme is in cartoon representation and compound in ball-stick representation. The interacting residues were shown in the stick form with their distances (shown in dashes).

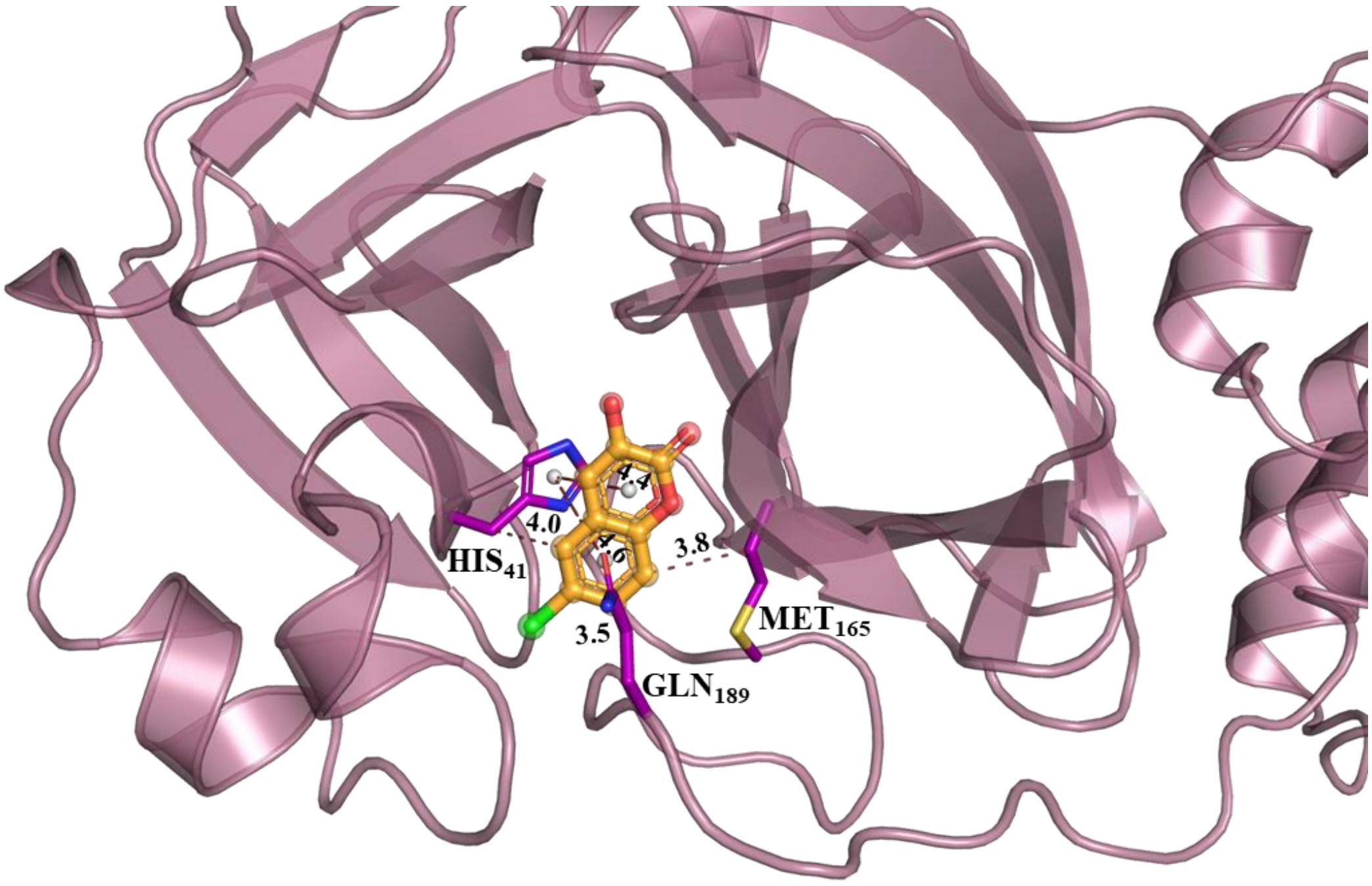

Figure S16. The interaction between 3CLpro enzyme (PDB 6LU7) and the compound **8b**. The enzyme is in cartoon representation and compound in ball-stick representation. The interacting residues were shown in the stick form with their distances (shown in dashes).

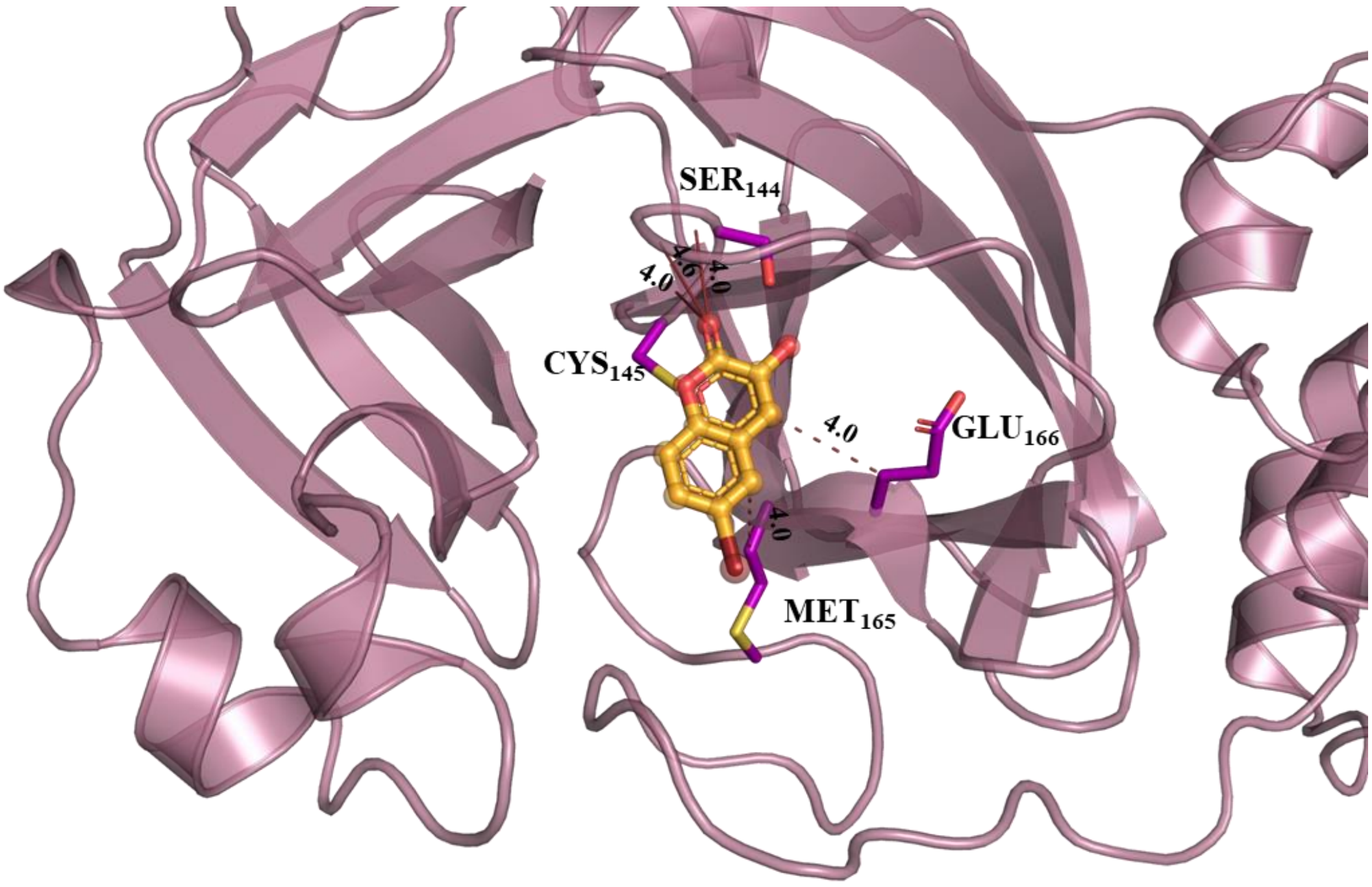

Figure S17. The interaction between 3CLpro enzyme (PDB 6LU7) and the compound **8c**. The enzyme is in cartoon representation and compound in ball-stick representation. The interacting residues were shown in the stick form with their distances (shown in dashes).

Table S1: Distance of Interacting Residues atoms of 3CLpro with atoms of compound **3a**.

**Residue**	**Residue atom**	**Ligand**	**Ligand atom**	**Distance**
GLU166	CB	LIG307	C3	3.8
MET165	CB	LIG307	C11	3.8
HIS41	CB	LIG307	C5	3.8
GLN189	CG	LIG307	C2	3.8
HIS41	Pie Stacking	LIG307	Pie Stacking	4.5

Table S2: Distance of Interacting Residues atoms of 3CLpro with atoms of compound **3b**.

**Residue**	**Residue atom**	**Ligand**	**Ligand atom**	**Distance**
GLN189	CG	LIG307	C18	3.6
LEU27	CD2	LIG307	C11	3.8
MET165	CB	LIG307	C17	3.6
GLY143	CA	LIG307	O20	4.9
HIS41	Pie Stacking	LIG307	Pie Stacking	4.8

Table S3: Distance of Interacting Residues atoms of 3CLpro with atoms of compound **3c**.

**Residue**	**Residue atom**	**Ligand**	**Ligand atom**	**Distance**
GLN189	CG	LIG307	C15	3.6
THR25	CG2	LIG307	C3	3.9
MET165	CB	LIG307	C17	3.8
MET49	CB	LIG307	C5	3.8
HIS41	Pie Stacking	LIG307	Pie Stacking	4.7

Table S4: Distance of Interacting Residues atoms of 3CLpro with atoms of compound **3d**.

**Residue**	**Residue atom**	**Ligand**	**Ligand atom**	**Distance**
GLN189	CG	LIG307	C2	3.7
THR25	CG2	LIG307	C11	3.9
MET165	CB	LIG307	C10	3.6
LEU27	CD2	LIG307	C6	3.7
HIS41	Pie Stacking	LIG307	Pie Stacking	4.8

Table S5: Distance of Interacting Residues atoms of 3CLpro with atoms of compound **3e**.

Residue	Residue atom	Ligand	Ligand atom	Distance
PHE140	CB	LIG307	C4	3.8
GLU166	CB	LIG307	C7	3.7
MET165	CB	LIG307	C2	3.6
ASN142	CA	LIG307	O17	4.7

Table S6: Distance of Interacting Residues atoms of 3CLpro with atoms of compound **3f**.

Residue	Residue atom	Ligand	Ligand atom	Distance
GLN189	CG	LIG307	C10	3.6
LEU27	CD2	LIG307	C5	3.8
MET165	CB	LIG307	C16	3.6
GLY143	CA	LIG307	O20	4.9
HIS41	Pie Stacking	LIG307	Pie Stacking	4.8

Table S7: Distance of Interacting Residues atoms of 3CLpro with atoms of compound **3g**.

Residue	Residue atom	Ligand	Ligand atom	Distance
PHE140	CB	LIG307	C1	3.8
GLU166	CB	LIG307	C5	3.7
MET165	CB	LIG307	C14	3.7
HIS41	Pie Stacking	LIG307	Pie Stacking	4.2

Table S8: Distance of Interacting Residues atoms of 3CLpro with atoms of compound **3h**.

Residue	Residue atom	Ligand	Ligand atom	Distance
GLN189	CG	LIG307	C6	3.5
LEU27	CD2	LIG307	C11	3.8
MET165	CB	LIG307	C15	3.6
GLY143	CA	LIG307	O18	4.8
HIS41	Pie Stacking	LIG307	Pie Stacking	4.8

Table S9: Distance of Interacting Residues atoms of 3CLpro with atoms of compound **4a**.

Residue	Residue atom	Ligand	Ligand atom	Distance
GLN189	CG	LIG307	C13	3.8

Table S10: Distance of Interacting Residues atoms of 3CLpro with atoms of compound **6a**.

Residue	Residue atom	Ligand	Ligand atom	Distance
GLU166	CB	LIG307	C16	3.7
ASP187	CB	LIG307	C1	3.8
MET165	CB	LIG307	C18	3.7
HIS41	CB	LIG307	C6	3.7
GLN189	CB	LIG307	C13	4.0
HIS41	Pie Stacking	LIG307	Pie Stacking	4.0

Table S11: Distance of Interacting Residues atoms of 3CLpro with atoms of compound **6b**.

Residue	Residue atom	Ligand	Ligand atom	Distance
GLY143	CA	LIG307	O22	5.1
GLN189	CG	LIG307	C10	3.6
HIS41	Pie Stacking	LIG307	Pie Stacking	4.4

Table S12: Distance of Interacting Residues atoms of 3CLpro with atoms of compound **8a**.

Residue	Residue atom	Ligand	Ligand atom	Distance
MET49	CB	LIG307	C2	3.8
MET165	CB	LIG307	C1	3.8
HIS41	CB	LIG307	C6	4.0
GLN189	CG	LIG307	C10	3.5
HIS41	Pie Stacking	LIG307	Pie Stacking PS2	4.4
HIS41	Pie Stacking	LIG307	Pie Stacking PS1	4.6

Table S13: Distance of Interacting Residues atoms of 3CLpro with atoms of compound **8b**.

Residue	Residue atom	Ligand	Ligand atom	Distance
MET165	CB	LIG307	C9	3.8
HIS41	CB	LIG307	C6	4.0
GLN189	CG	LIG307	C3	3.5
HIS41	Pie Stacking	LIG307	Pie Stacking PS2	4.4
HIS41	Pie Stacking	LIG307	Pie Stacking PS1	4.6

Table S14: Distance of Interacting Residues atoms of 3CLpro with atoms of compound **8c**.

**Residue**	**Residue atom**	**Ligand**	**Ligand atom**	**Distance**
MET165	CB	LIG307	C6	4.0
GLU166	CB	LIG307	C2	4.0
GLY143	CA	LIG307	O12	4.0
SER144	CA	LIG307	O12	4.1
CYS145	CA	LIG307	O12	4.6

5. NMR Spectra

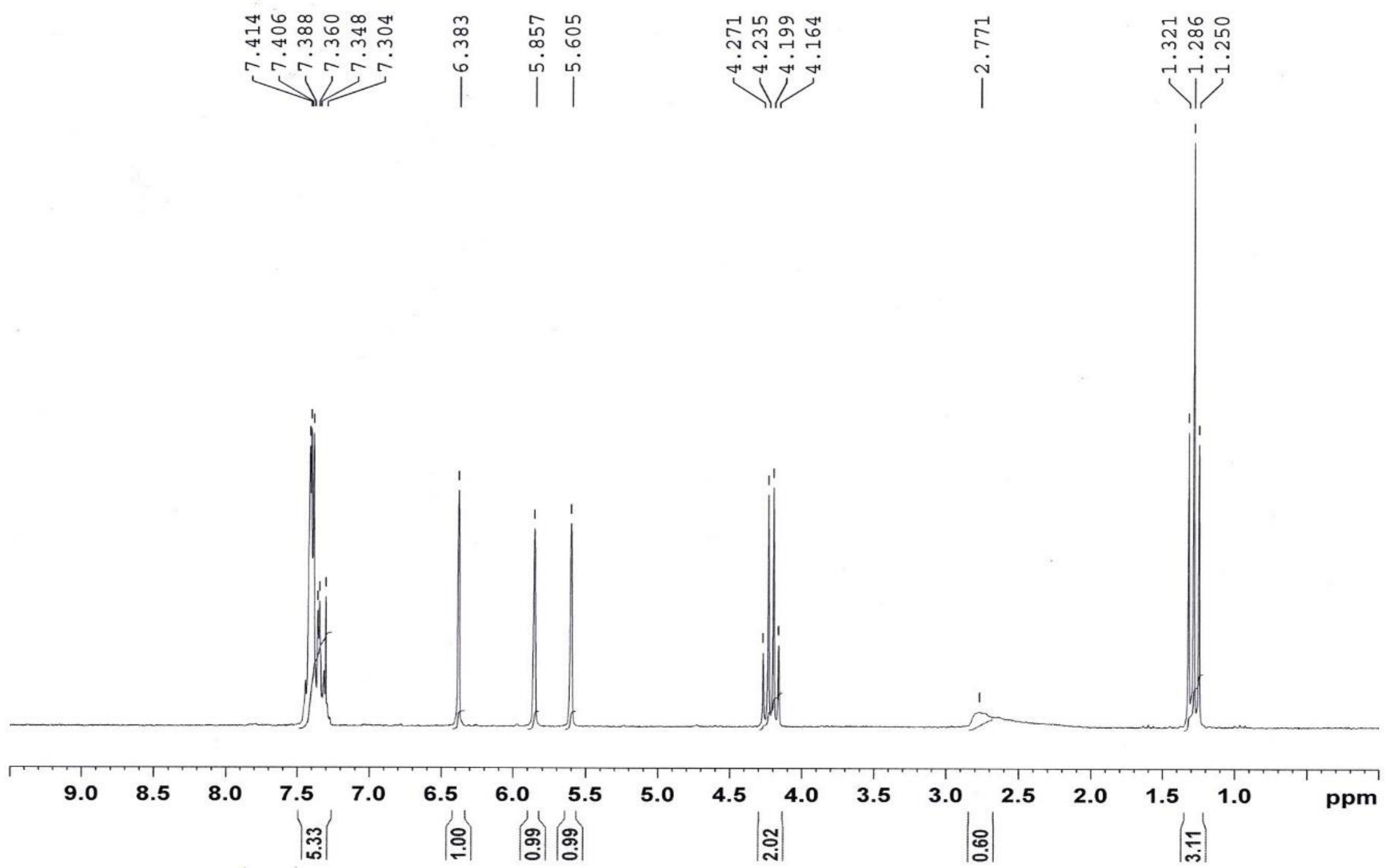

Figure S18. ^{1}H NMR spectrum of **2a** (200 MHz, $CDCl_3$).

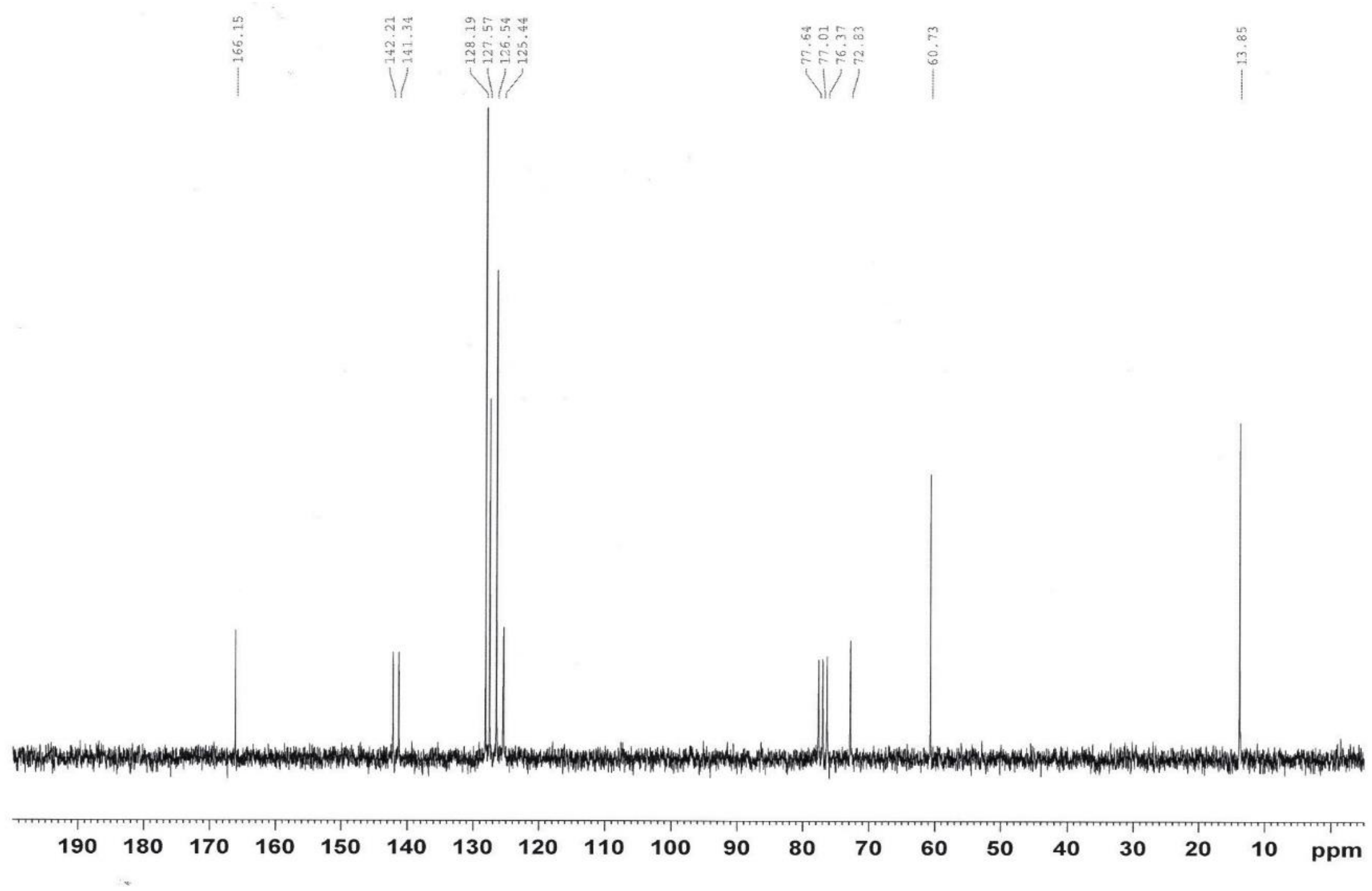

Figure S19. ^{13}C NMR spectrum of **2a** (50 MHz, $CDCl_3$).

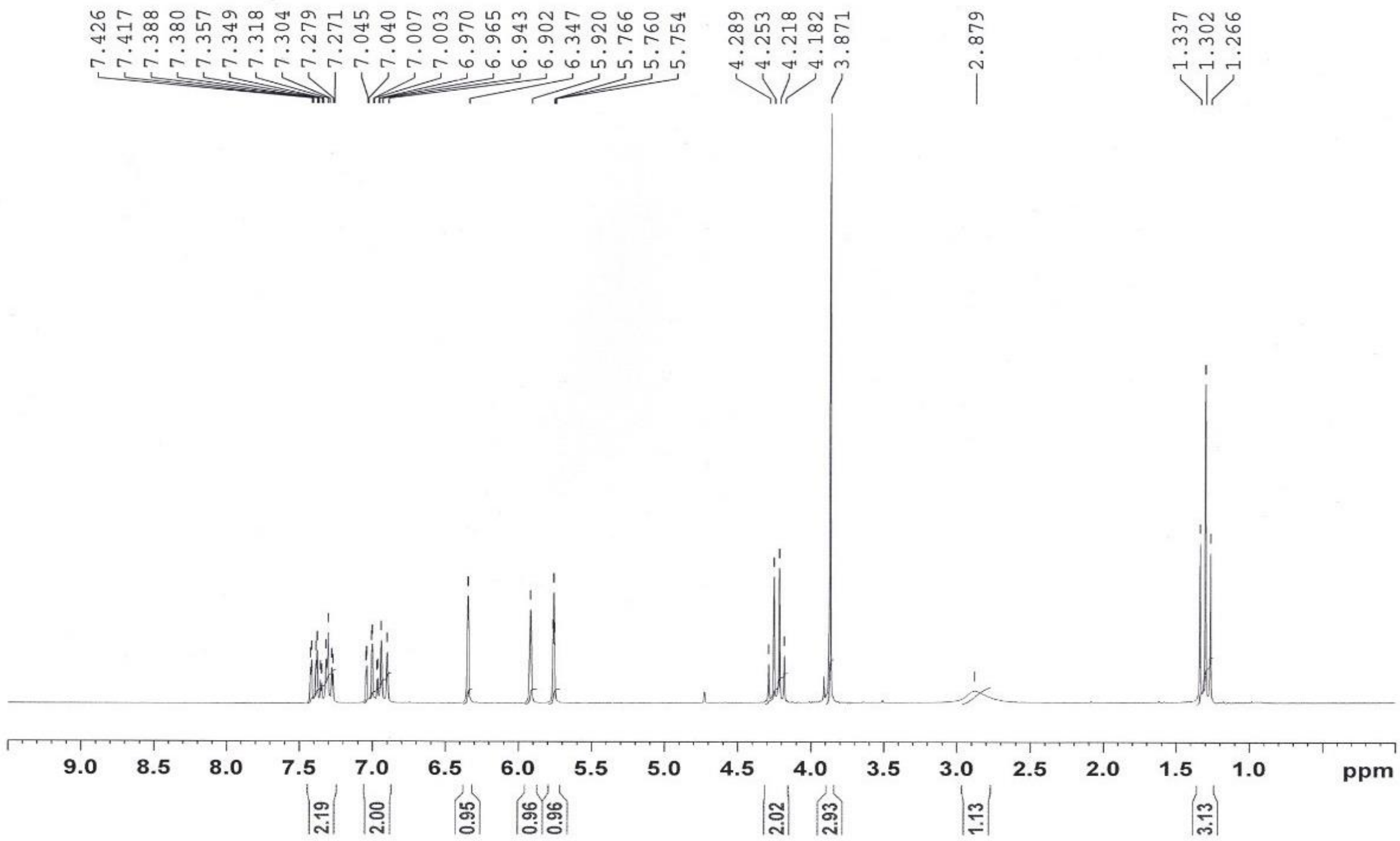

Figure S20. ^{1}H NMR spectrum of **2b** (200 MHz, $CDCl_3$).

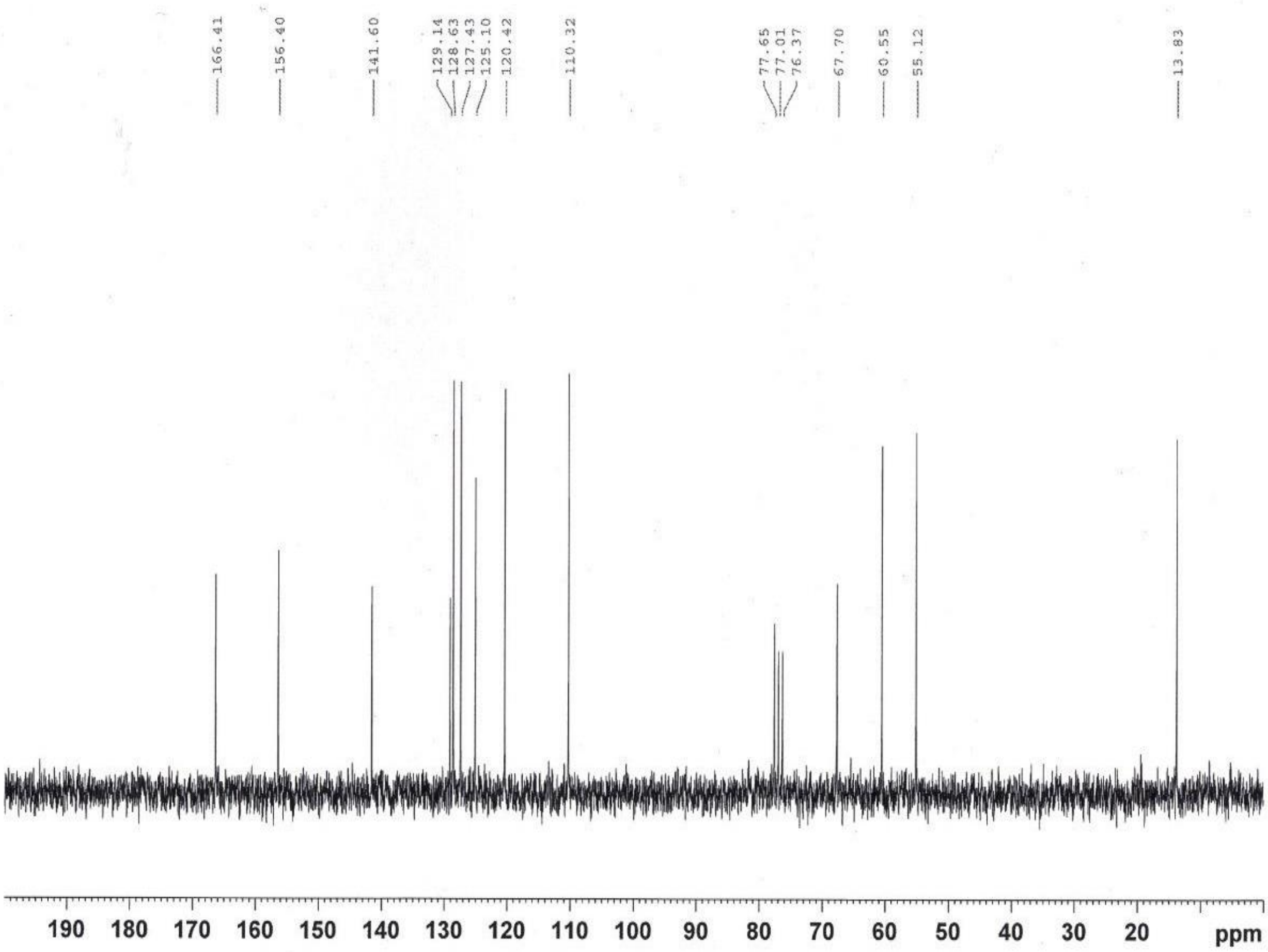

Figure S21. ^{13}C NMR spectrum of **2b** (50 MHz, $CDCl_3$).

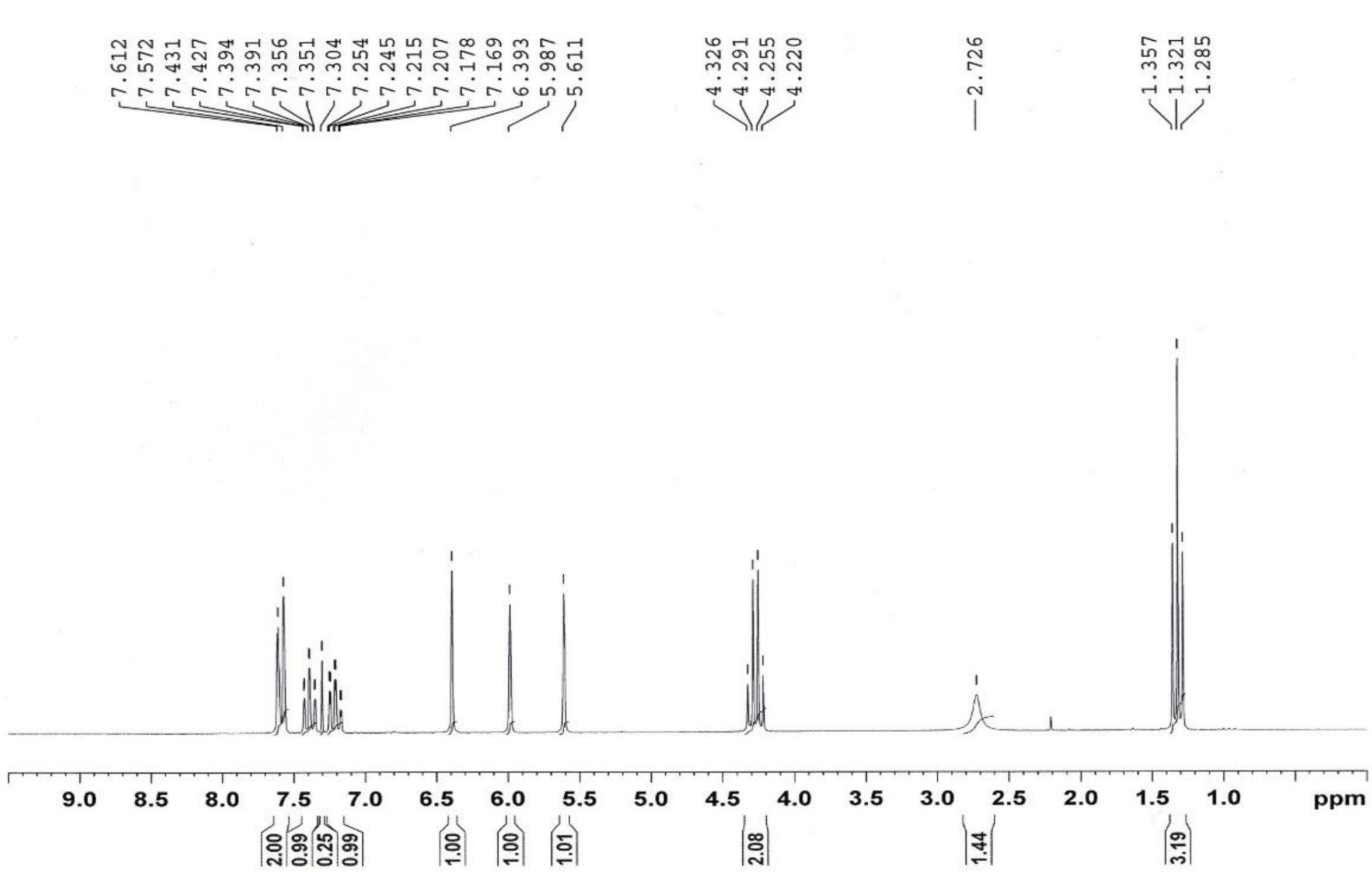

Figure S22. ^{1}H NMR spectrum of **2c** (200 MHz, $CDCl_3$).

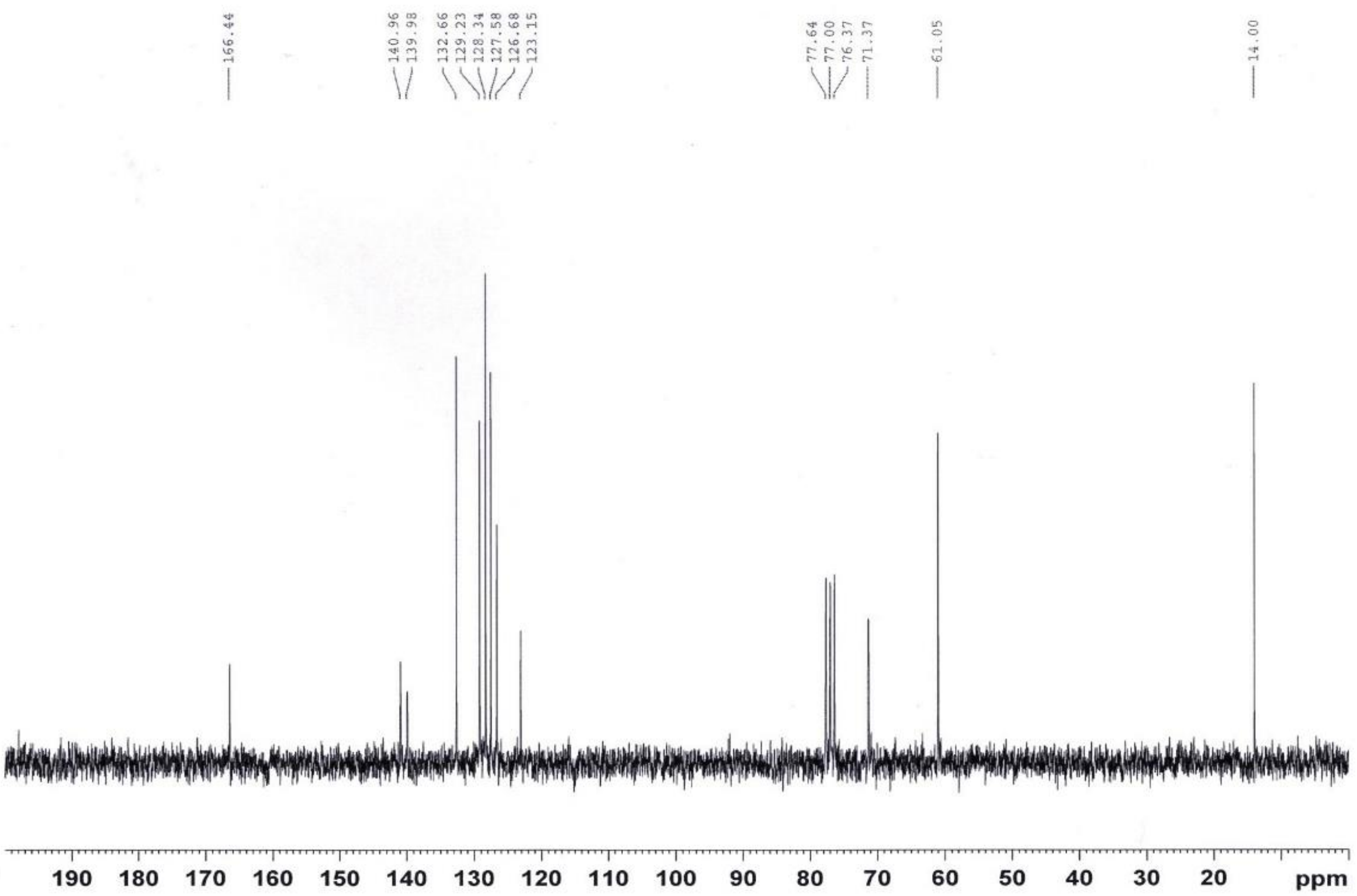

Figure S23. ^{13}C NMR spectrum of **2c** (50 MHz, $CDCl_3$).

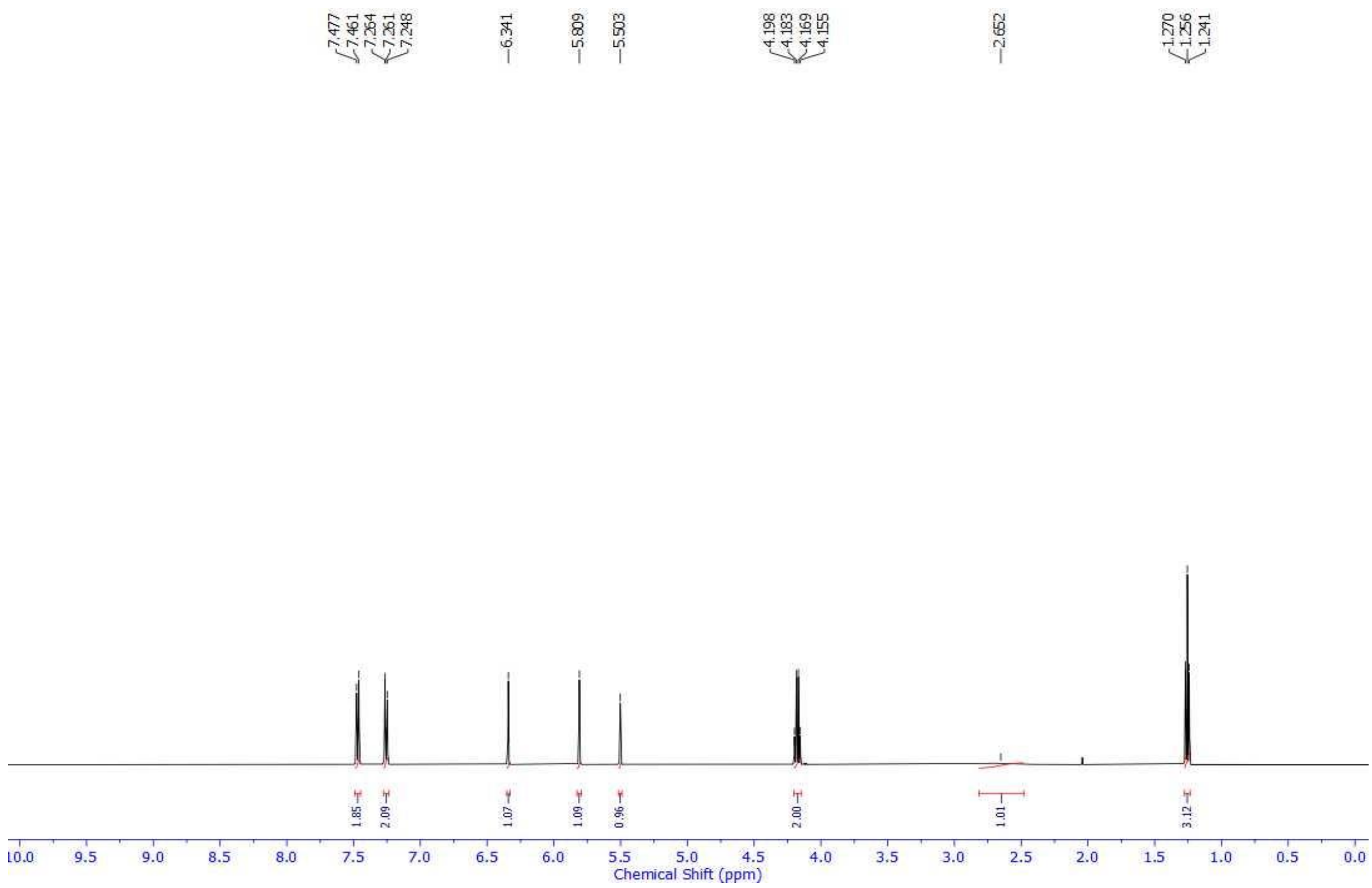

Figure S24. ^{1}H NMR spectrum of **2d** (500 MHz, $CDCl_3$).

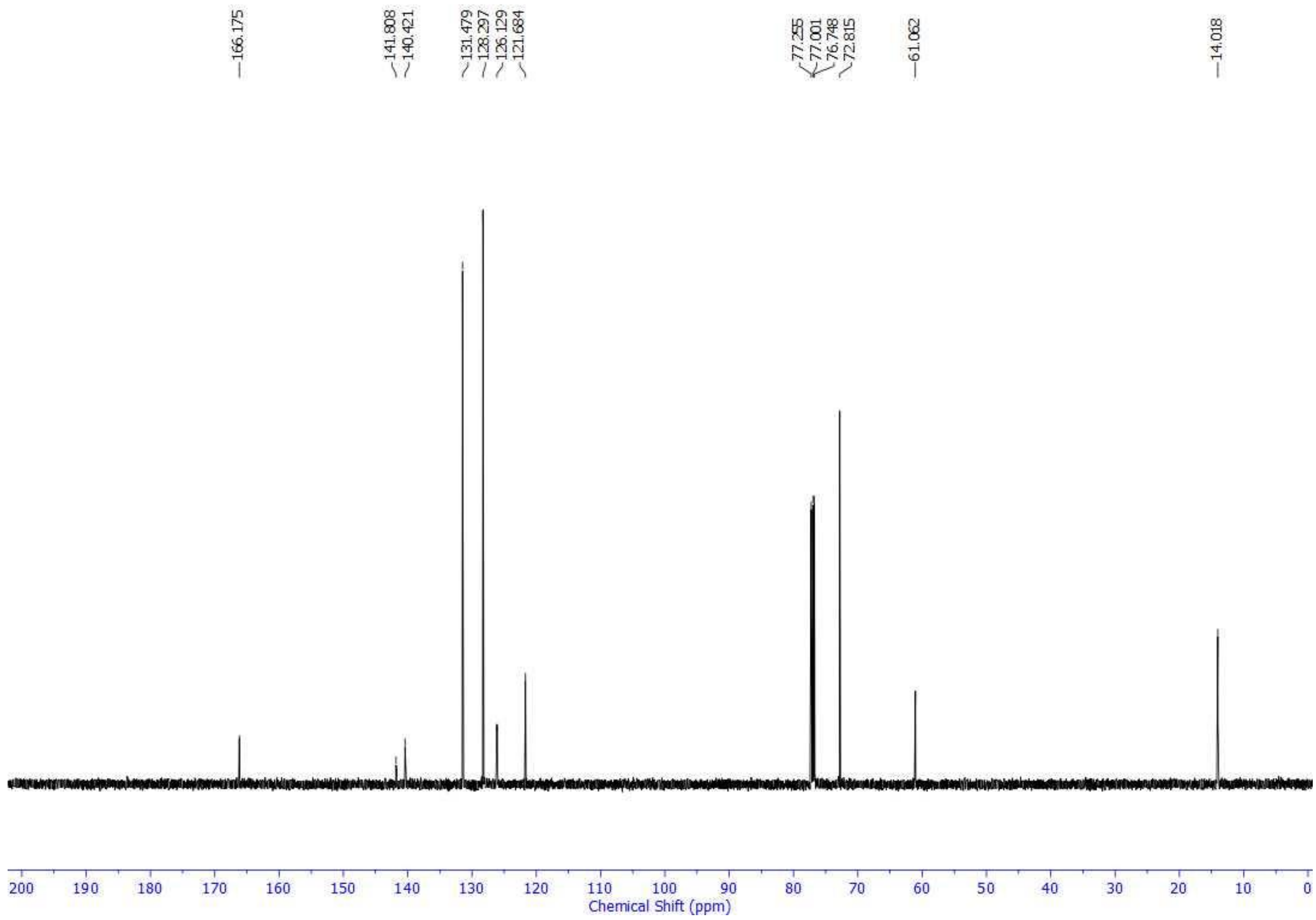

Figure S25. ^{13}C NMR spectrum of **2d** (125 MHz, $CDCl_3$).

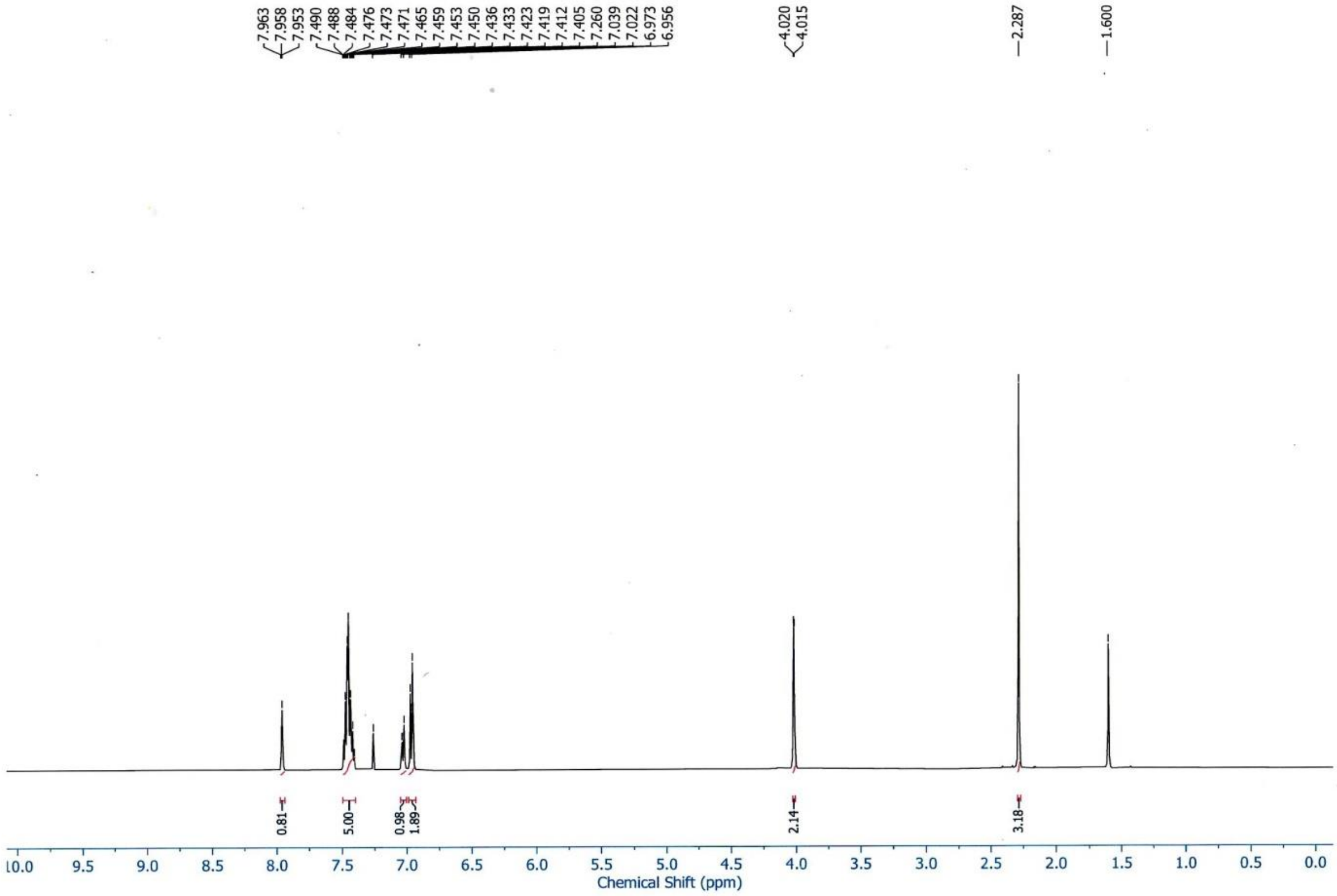

Figure S26. ^{1}H NMR spectrum of **3a** (500 MHz, $CDCl_3$).

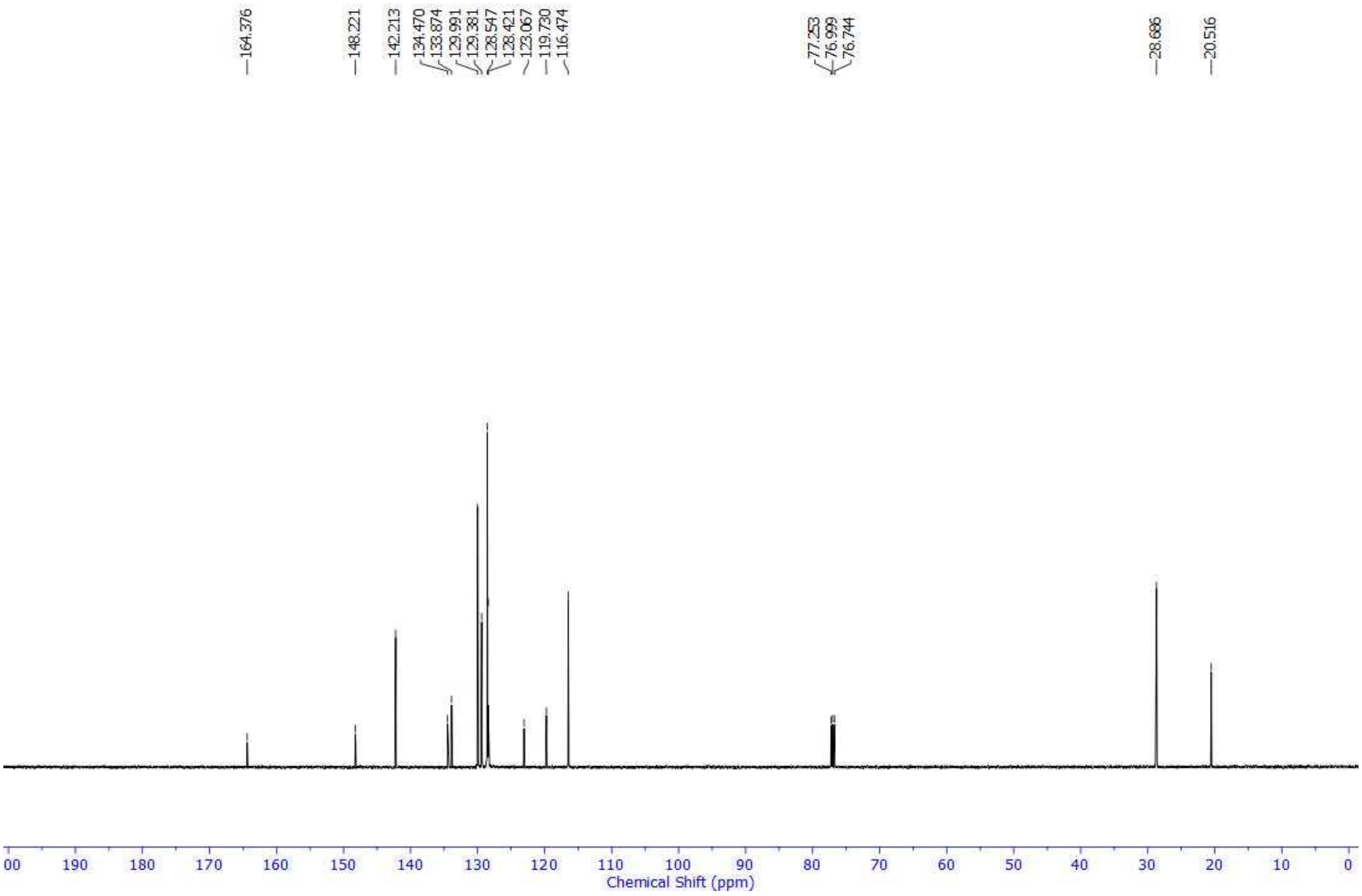

Figure S27. ^{13}C NMR spectrum of **3a** (125 MHz, $CDCl_3$).

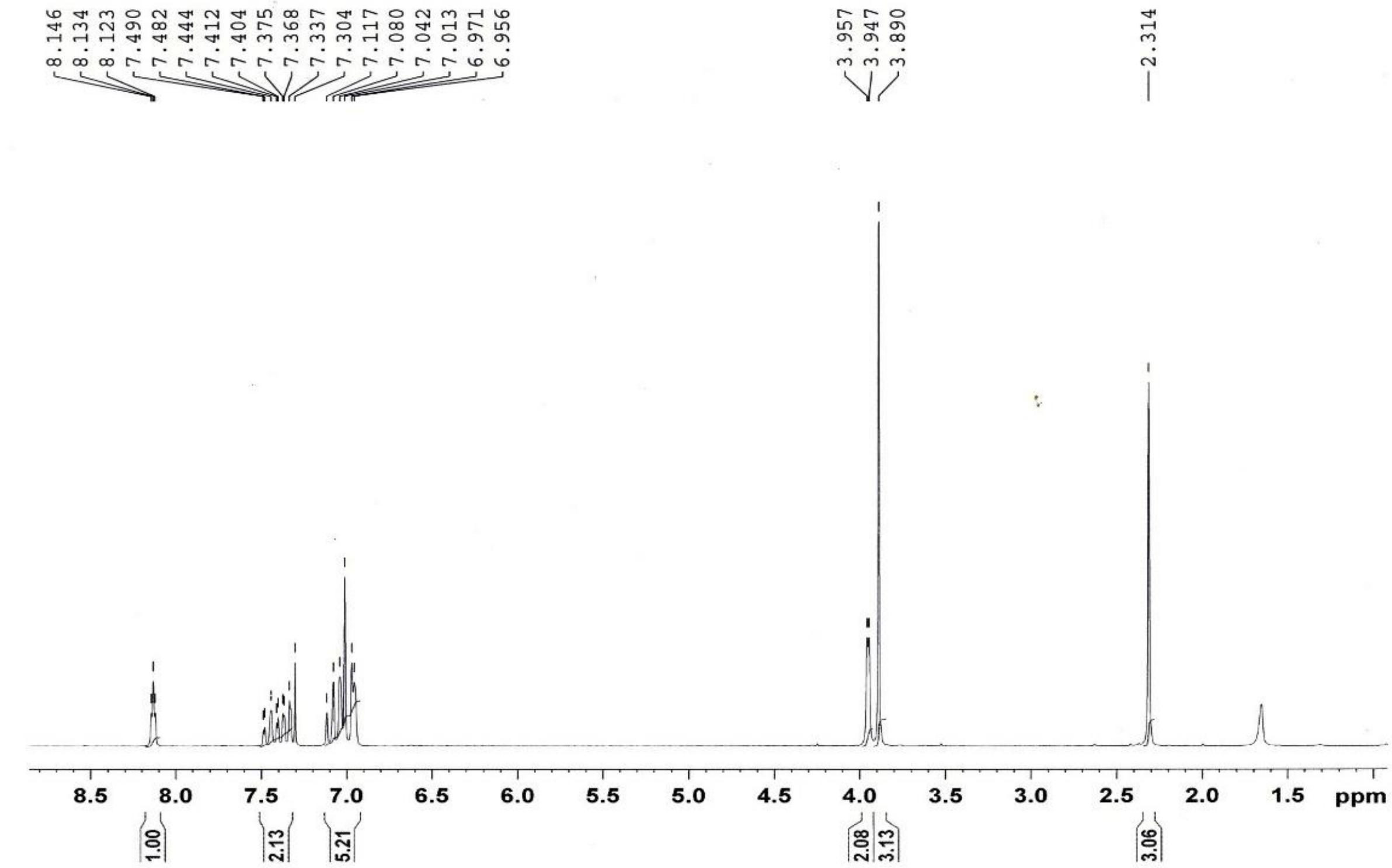

Figure S28. ^{1}H NMR spectrum of **3b** (200 MHz, $CDCl_3$).

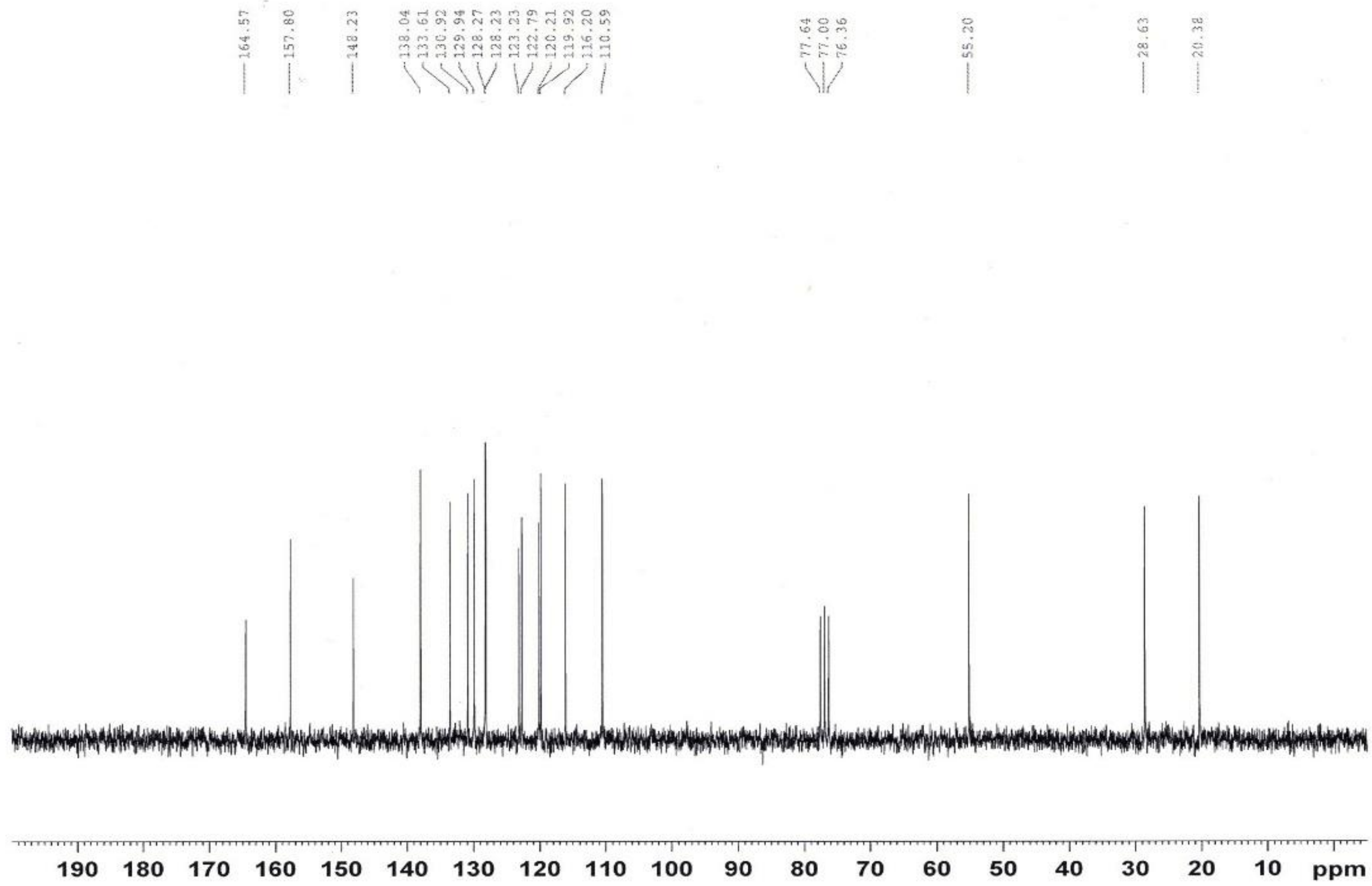

Figure S29. ^{13}C NMR spectrum of **3b** (50 MHz, $CDCl_3$).

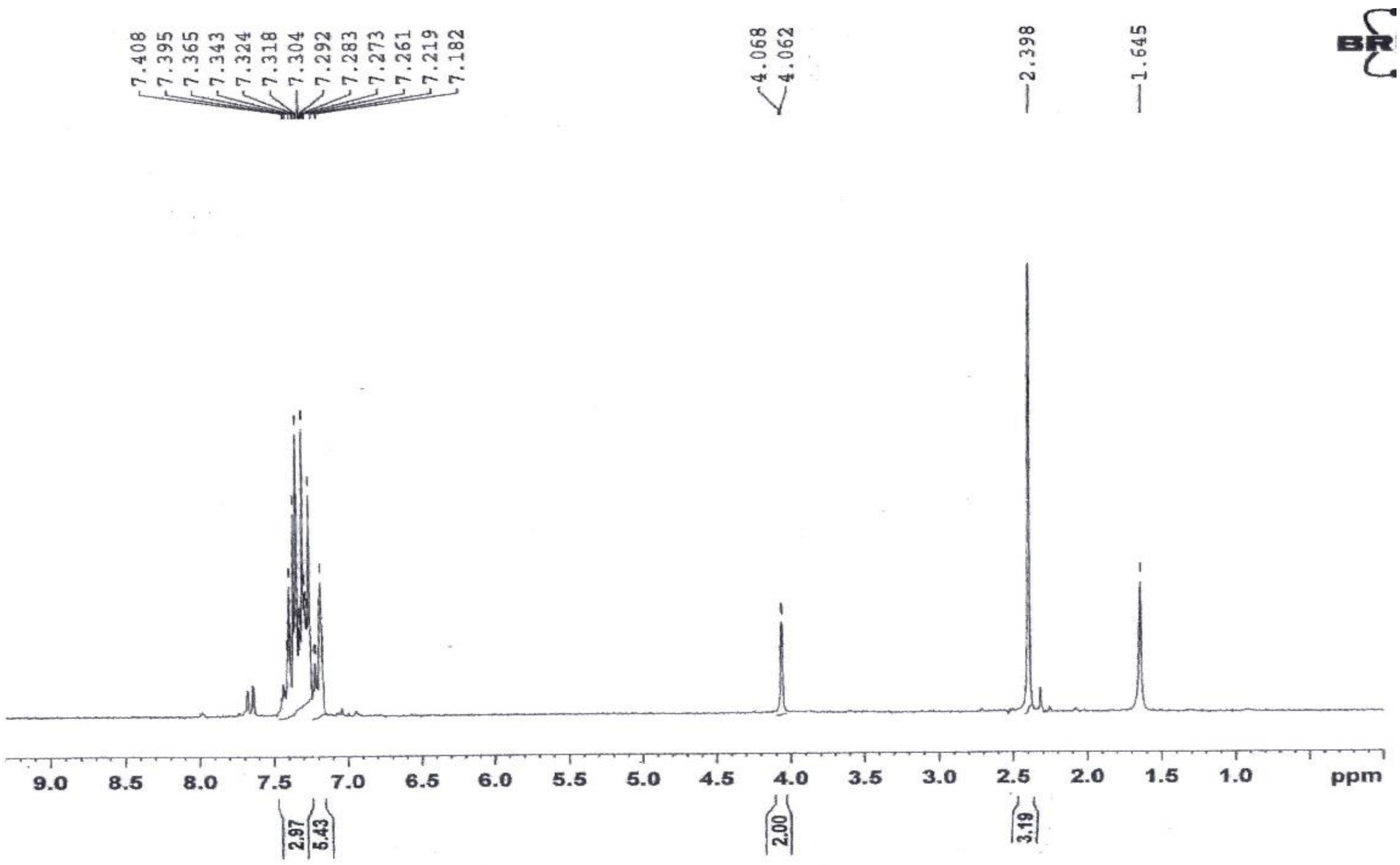

Figure S30. ^{1}H NMR spectrum of **3c** (200 MHz, $CDCl_3$).

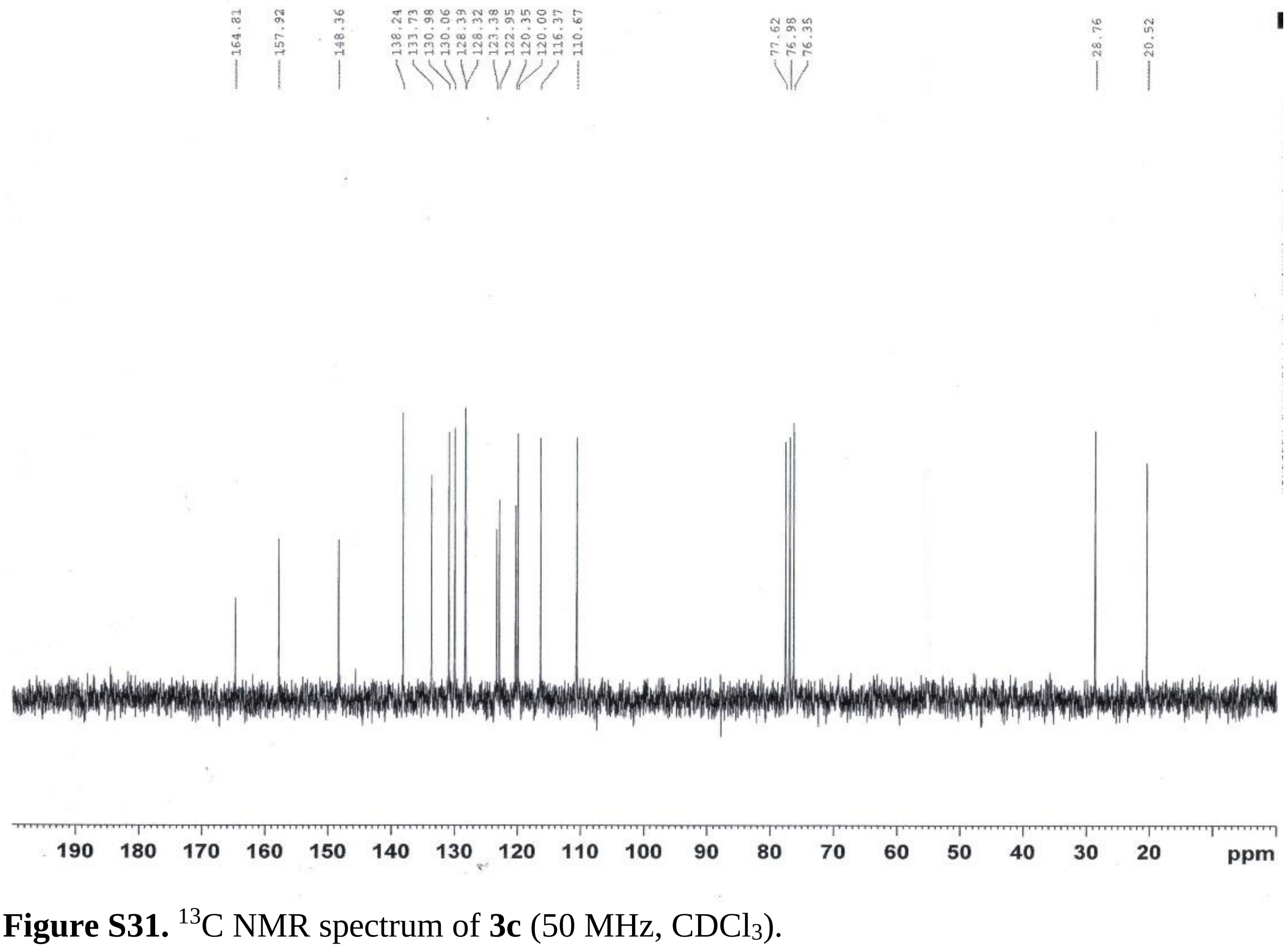

Figure S31. ^{13}C NMR spectrum of **3c** (50 MHz, $CDCl_3$).

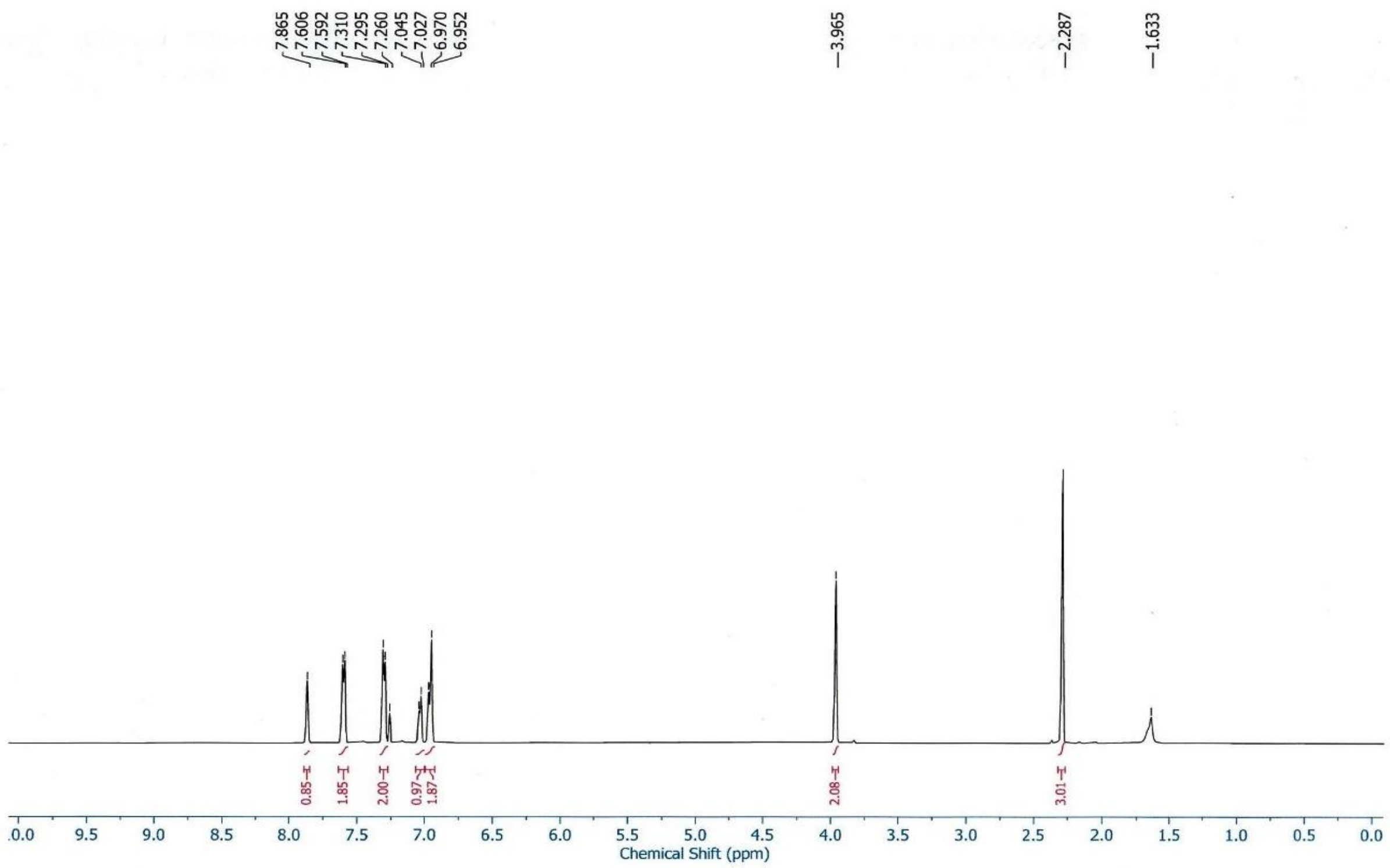

Figure S32. ^{1}H NMR spectrum of **3d** (500 MHz, $CDCl_3$).

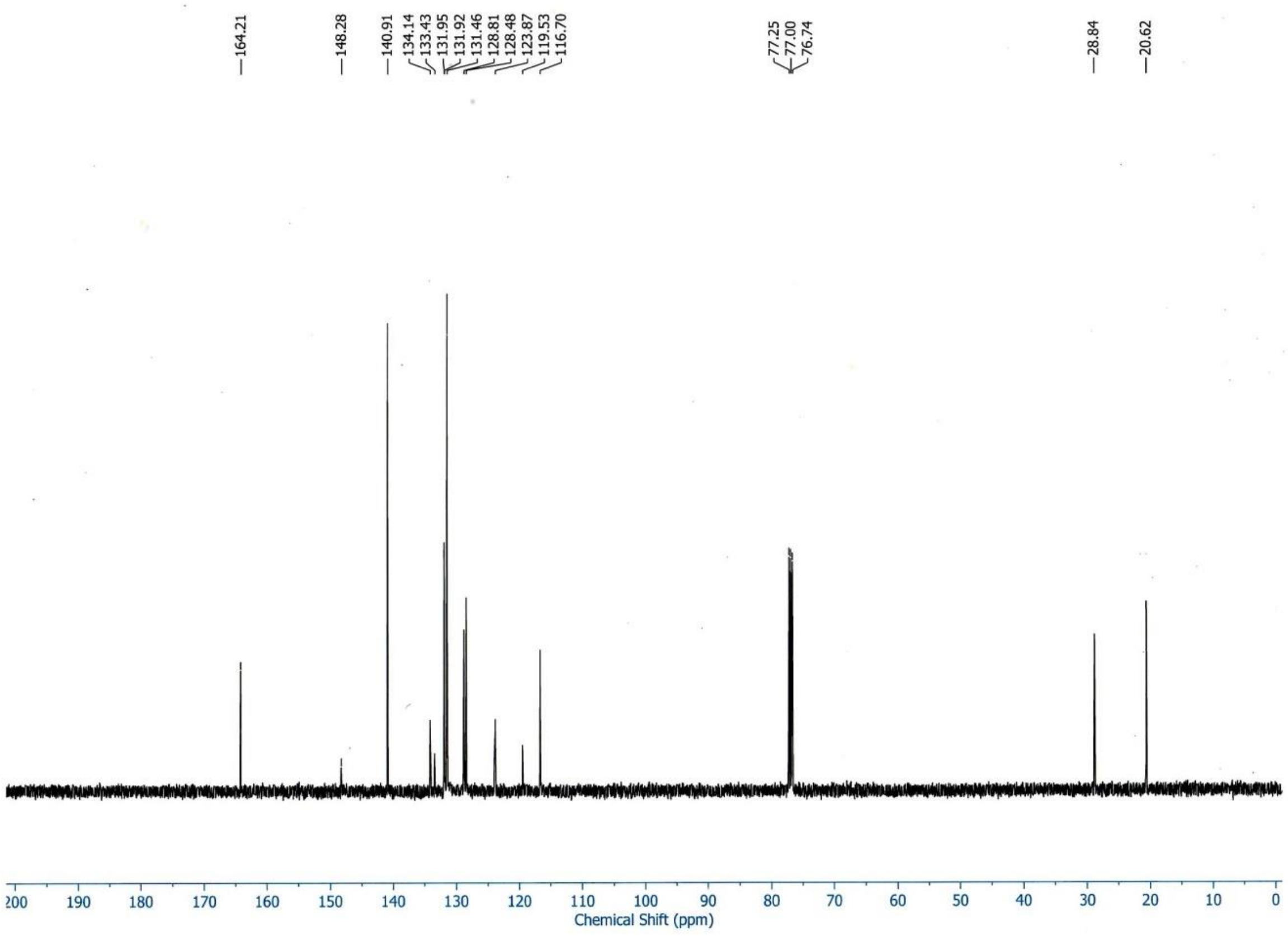

Figure S33. ^{13}C NMR spectrum of **3d** (125 MHz, $CDCl_3$).

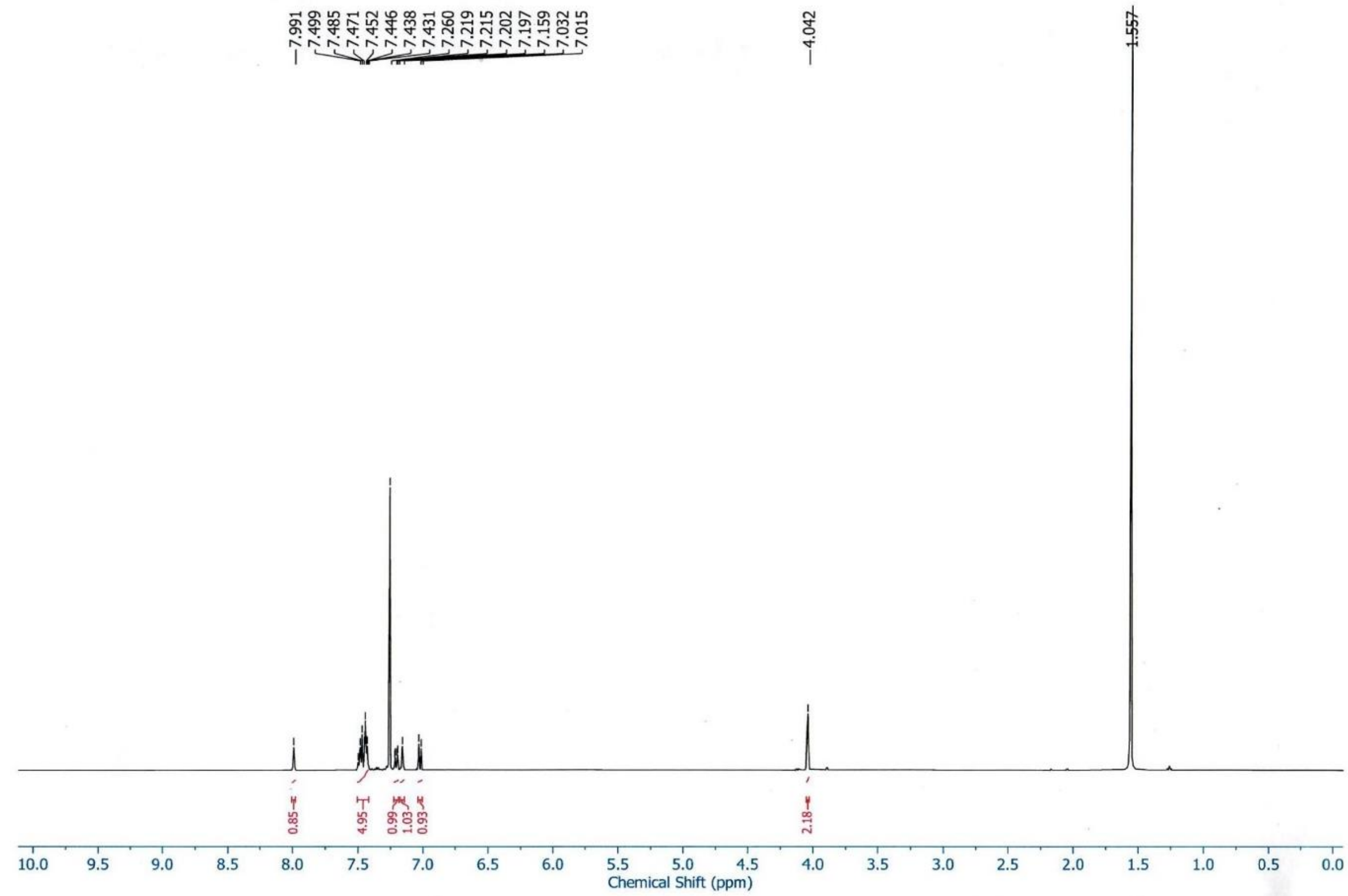

Figure S34. ^{1}H NMR spectrum of **3e** (500 MHz, $CDCl_3$).

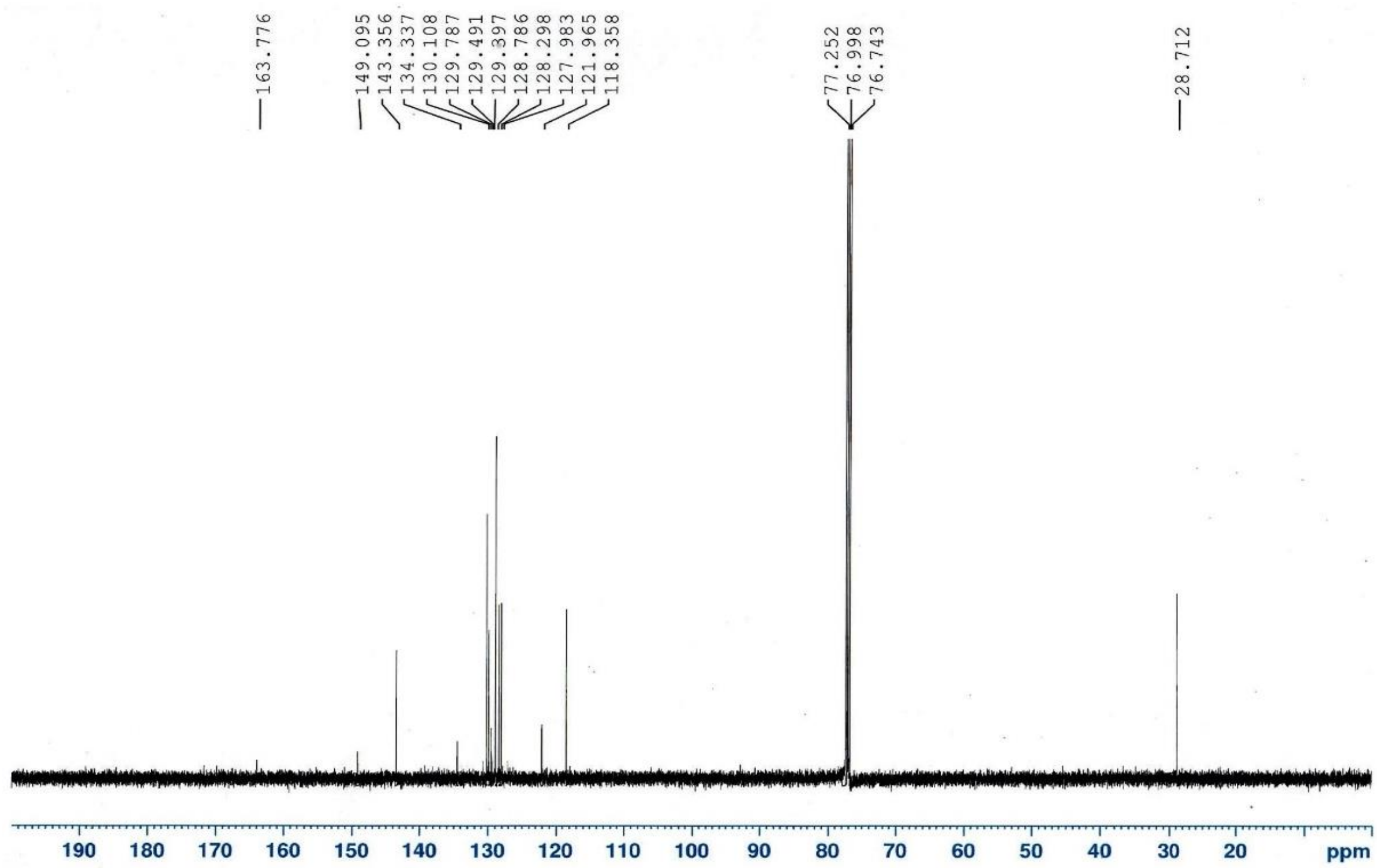

Figure S35. ^{13}C NMR spectrum of **3e** (125 MHz, $CDCl_3$).

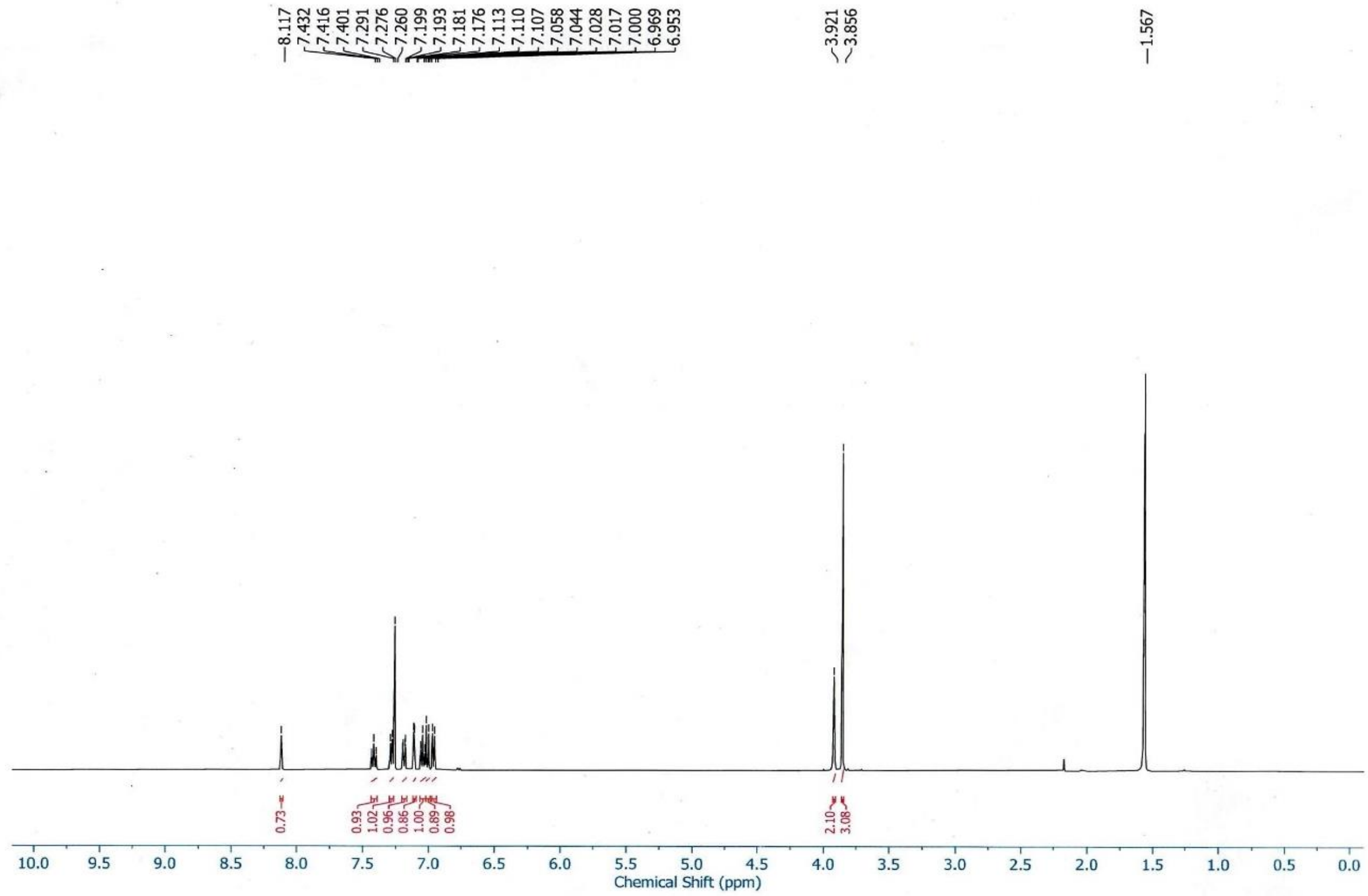

Figure S36. ^{1}H NMR spectrum of **3f** (500 MHz, $CDCl_3$).

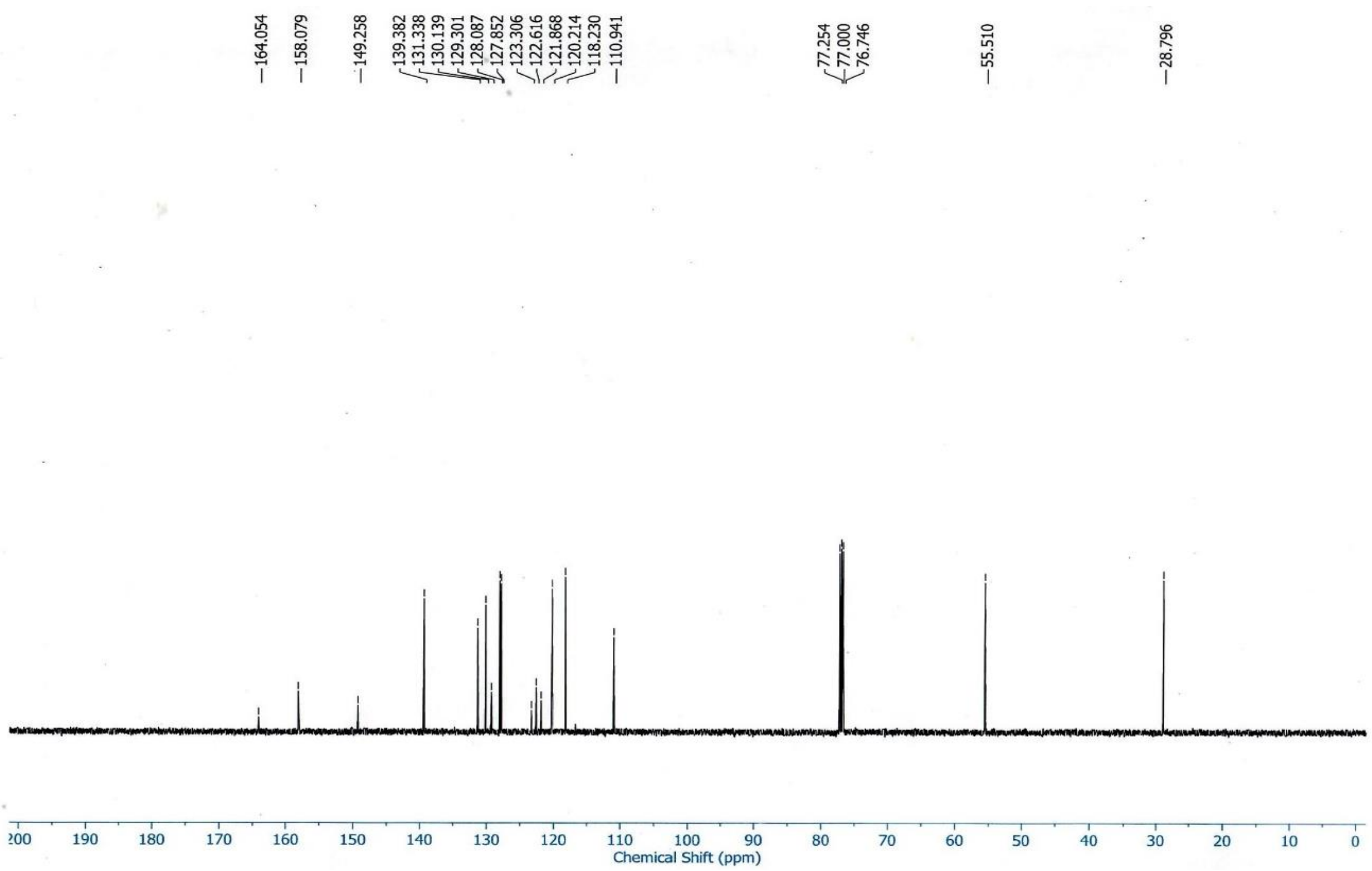

Figure S37. ^{13}C NMR spectrum of **3f** (125 MHz, $CDCl_3$).

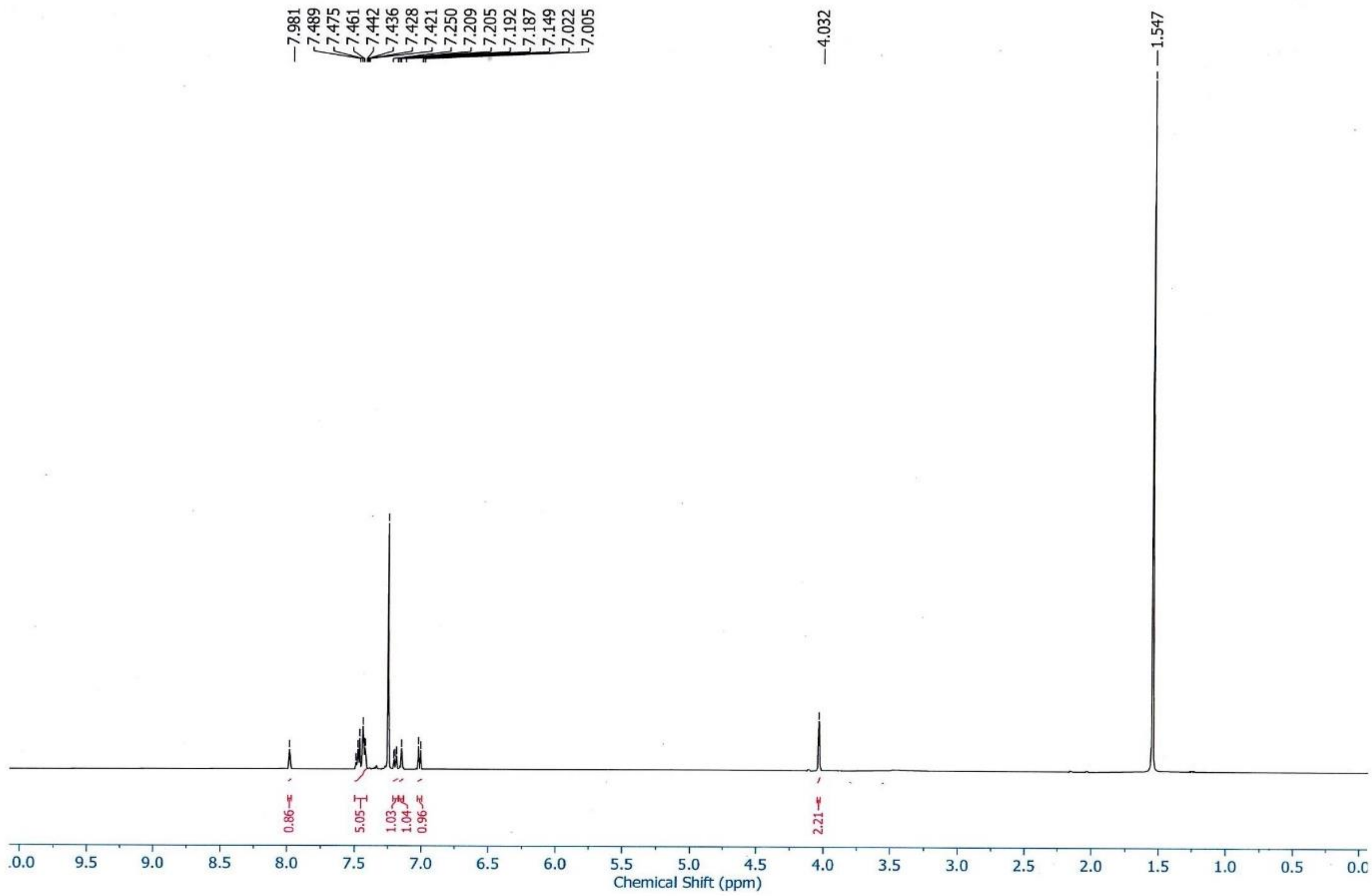

Figure S38. ^{1}H NMR spectrum of **3g** (500 MHz, $CDCl_3$).

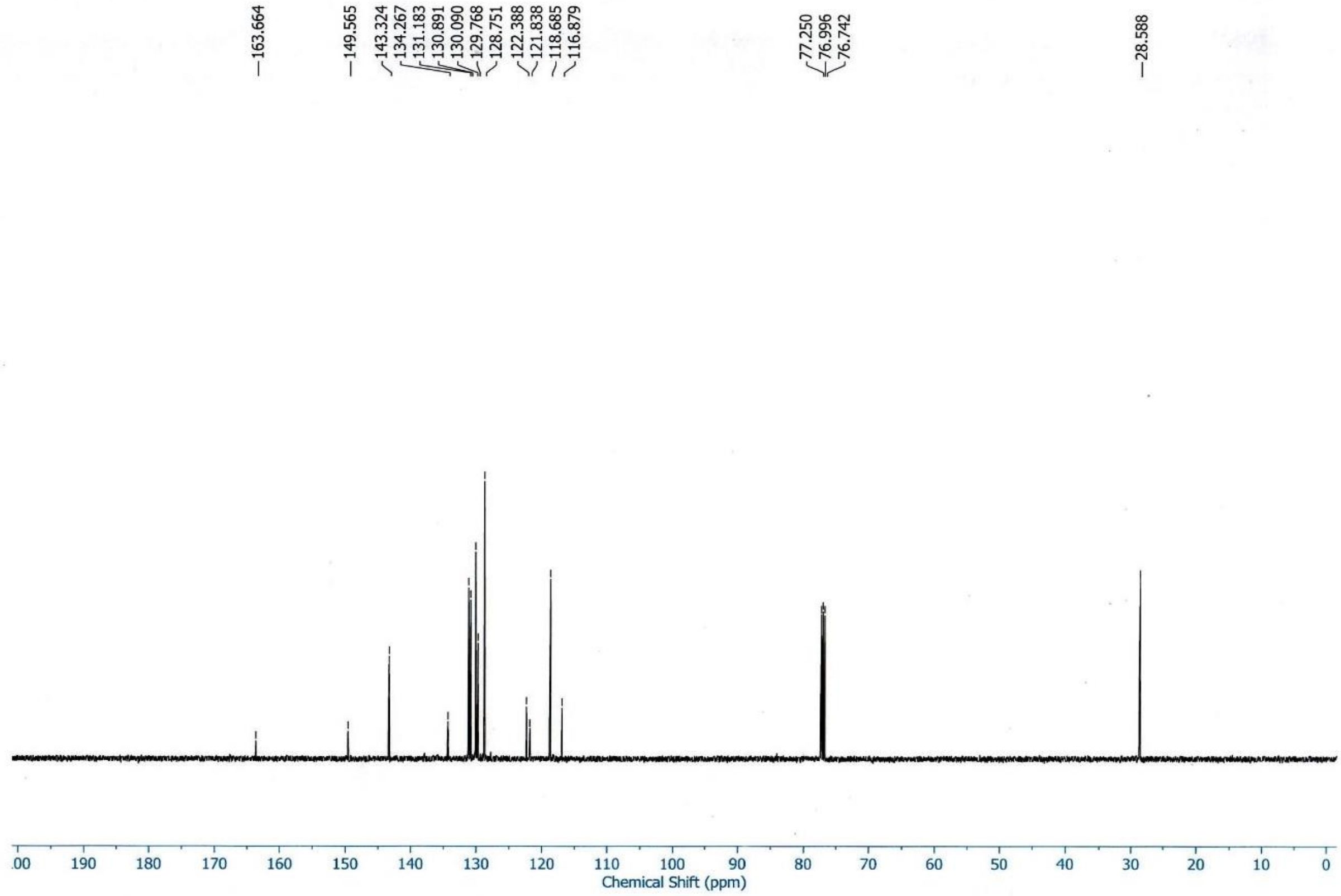

Figure S39. ^{13}C NMR spectrum of **3g** (125 MHz, $CDCl_3$).

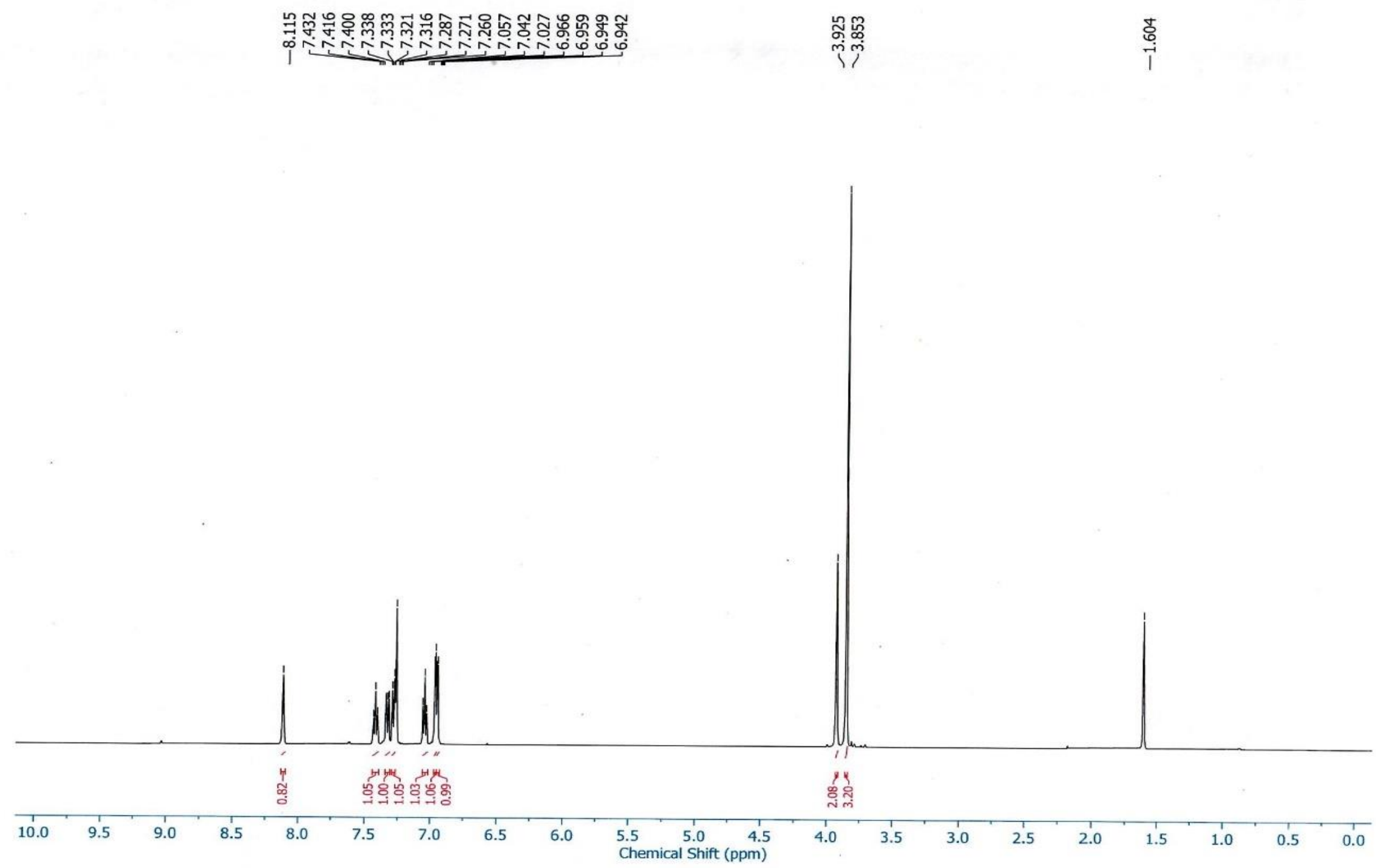

Figure S40. ^{1}H NMR spectrum of **3h** (500 MHz, $CDCl_3$).

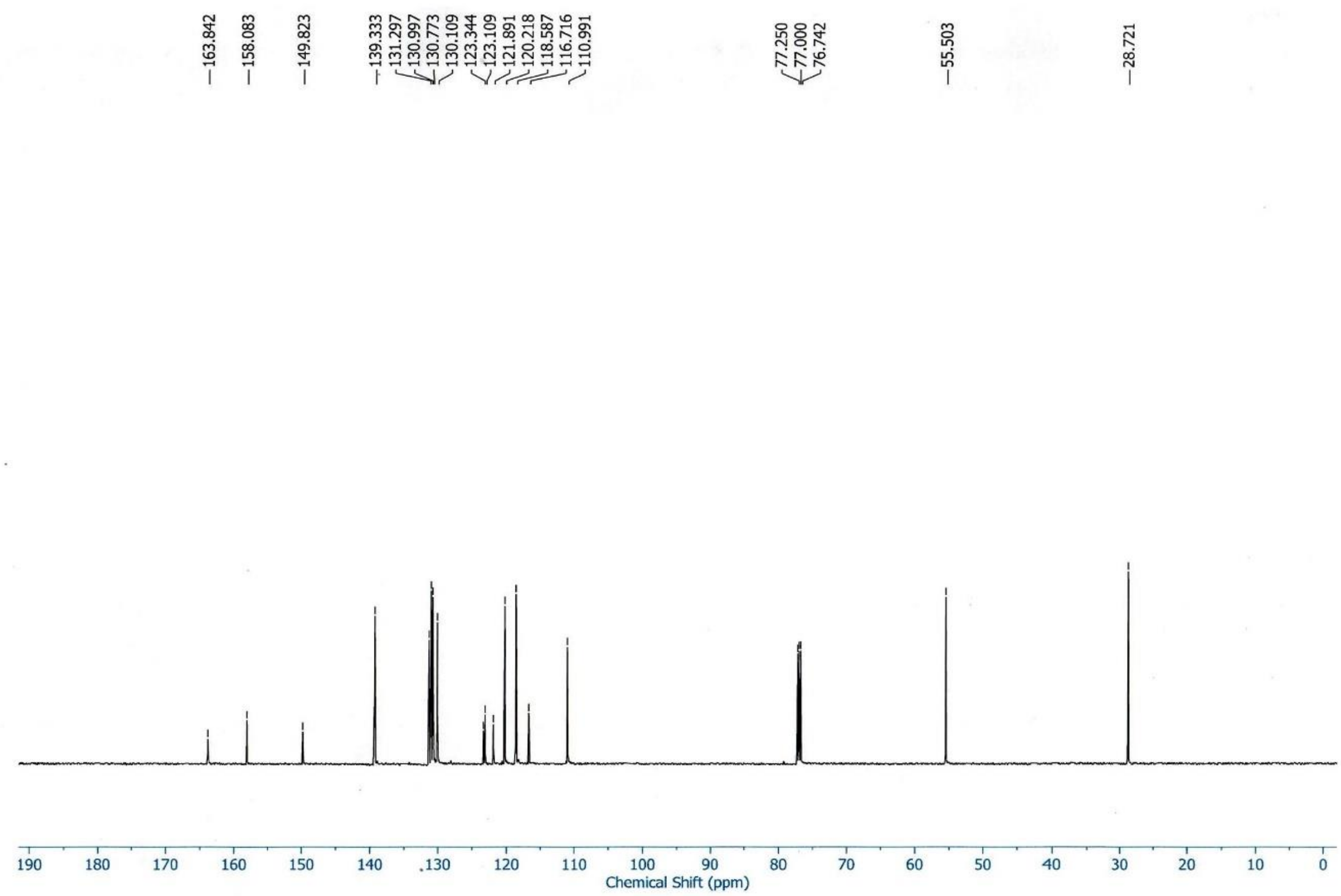

Figure S41. ^{13}C NMR spectrum of **3h** (125 MHz, CDCl3).

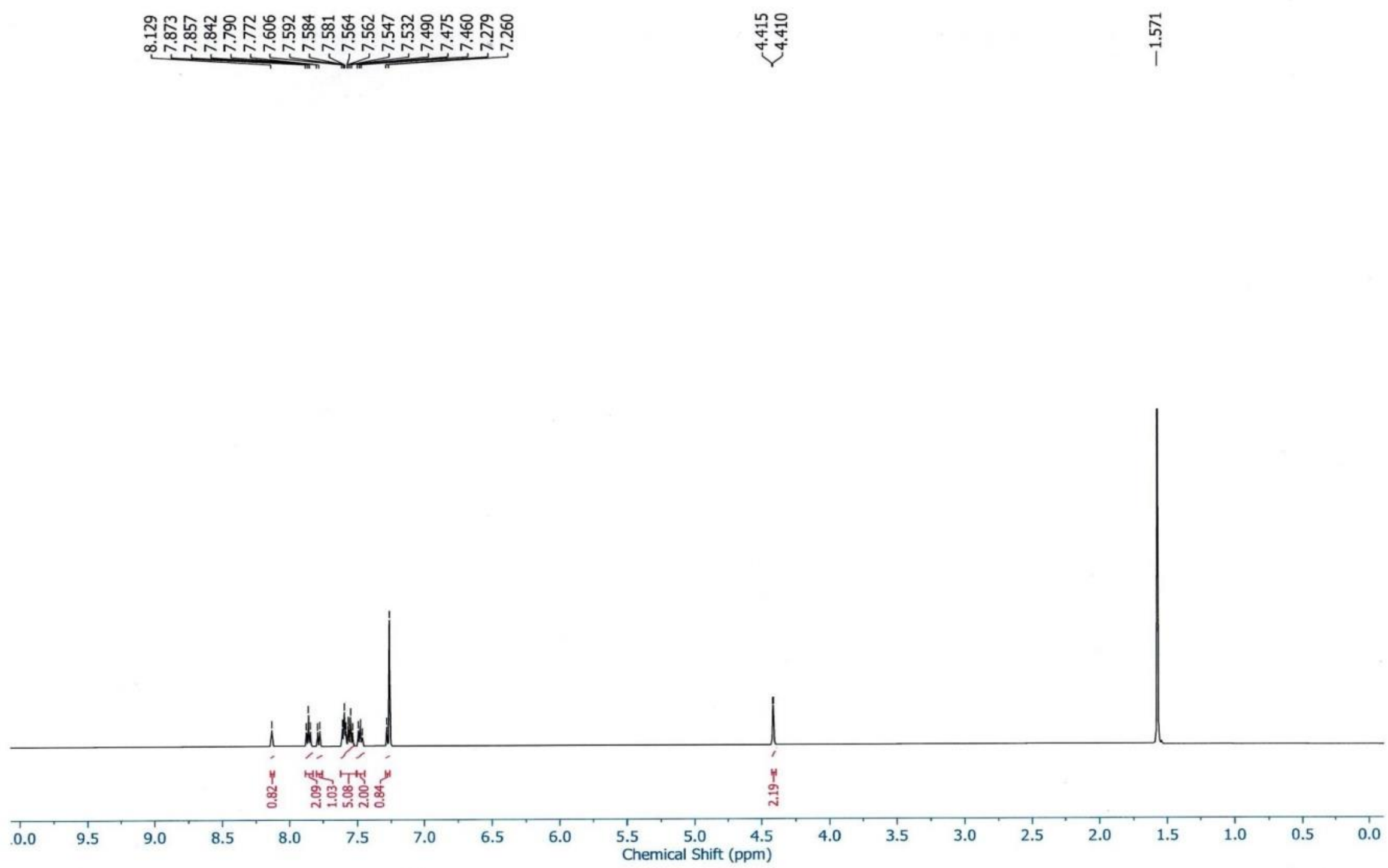

Figure S42. ^{1}H NMR spectrum of **6a** (500 MHz, $CDCl_3$).

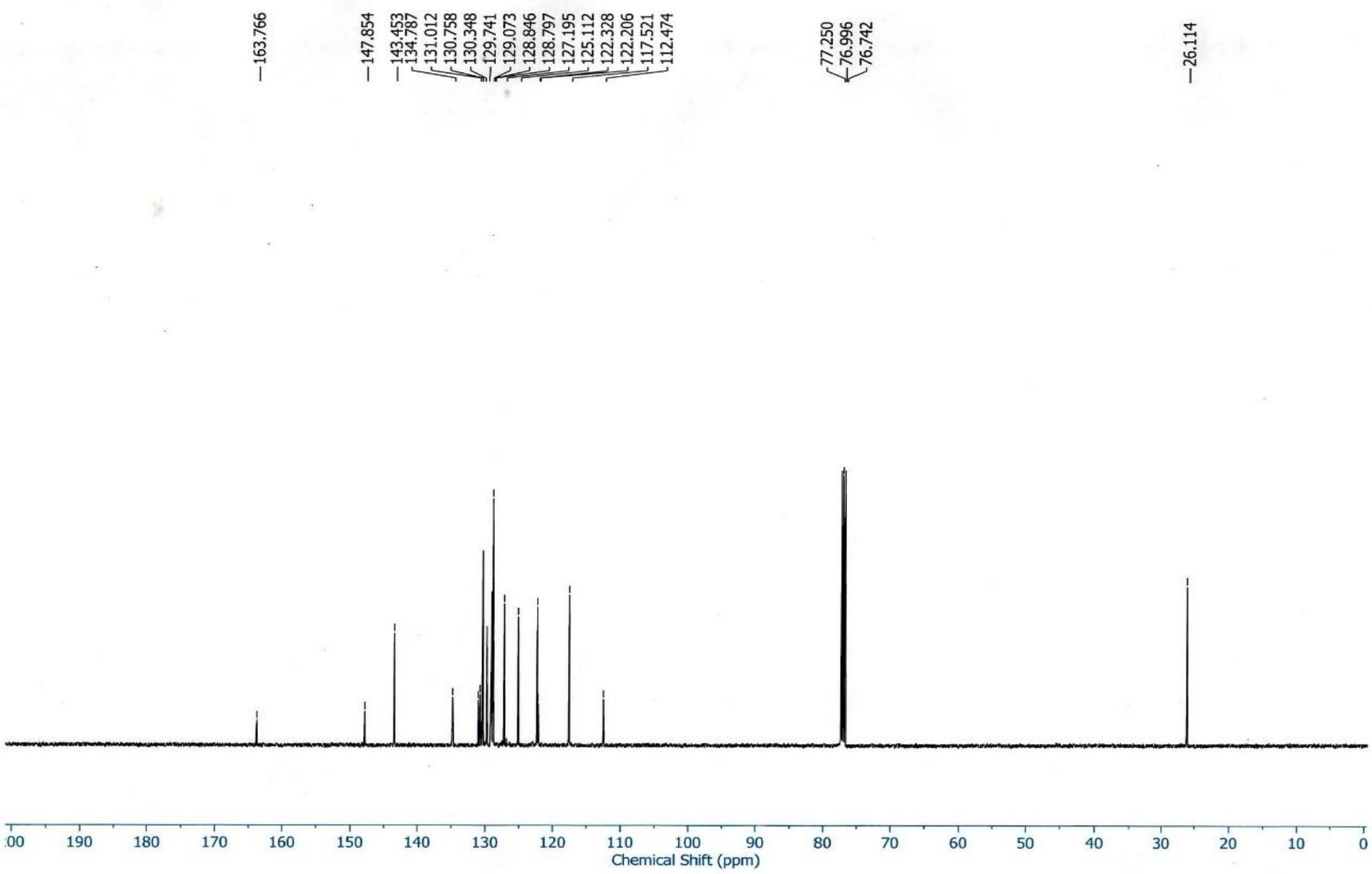

Figure S43. ^{13}C NMR spectrum of **6a** (125 MHz, CDCl3).

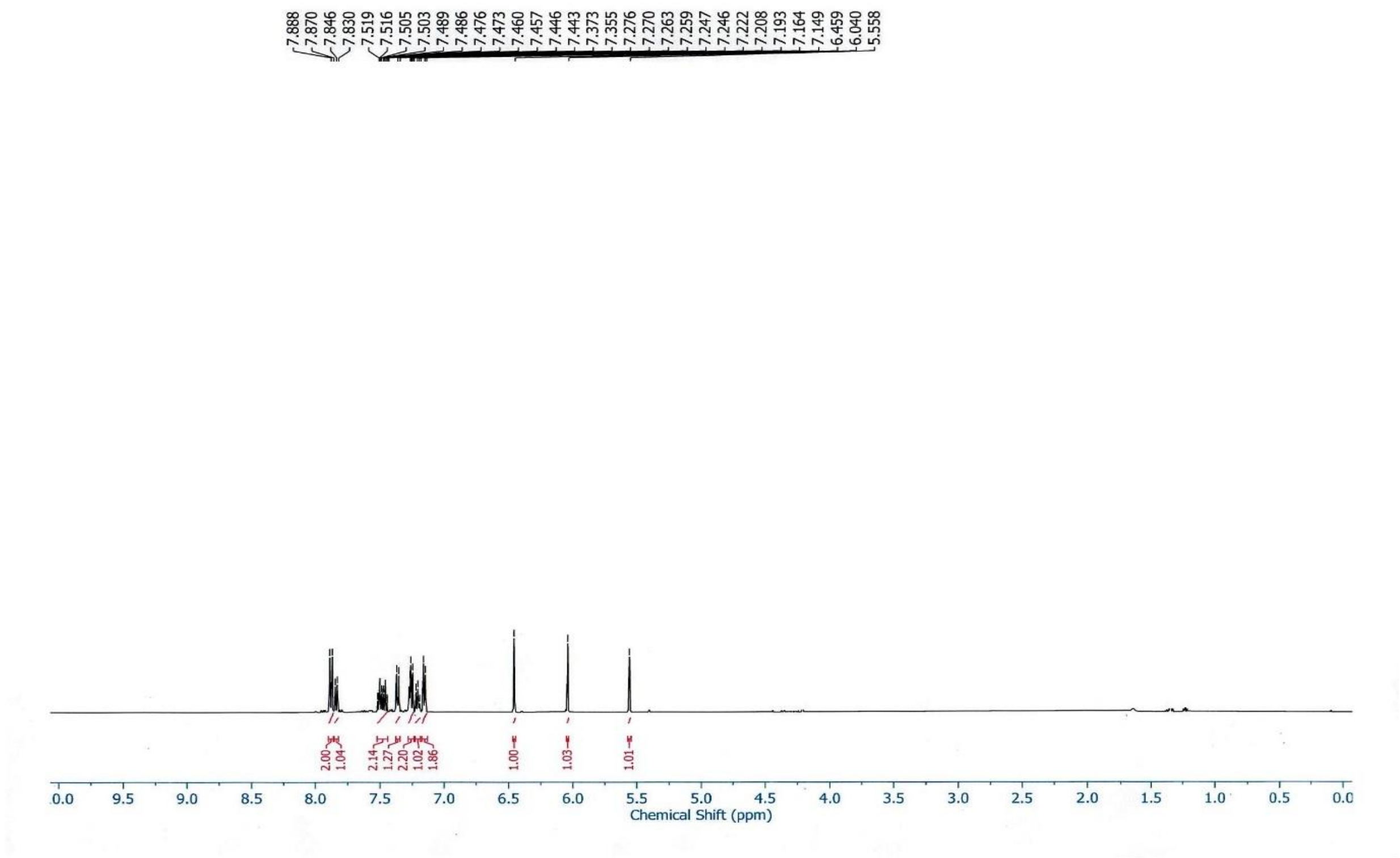

Figure S44. ^{1}H NMR spectrum of **6b** (500 MHz, $CDCl_3$).

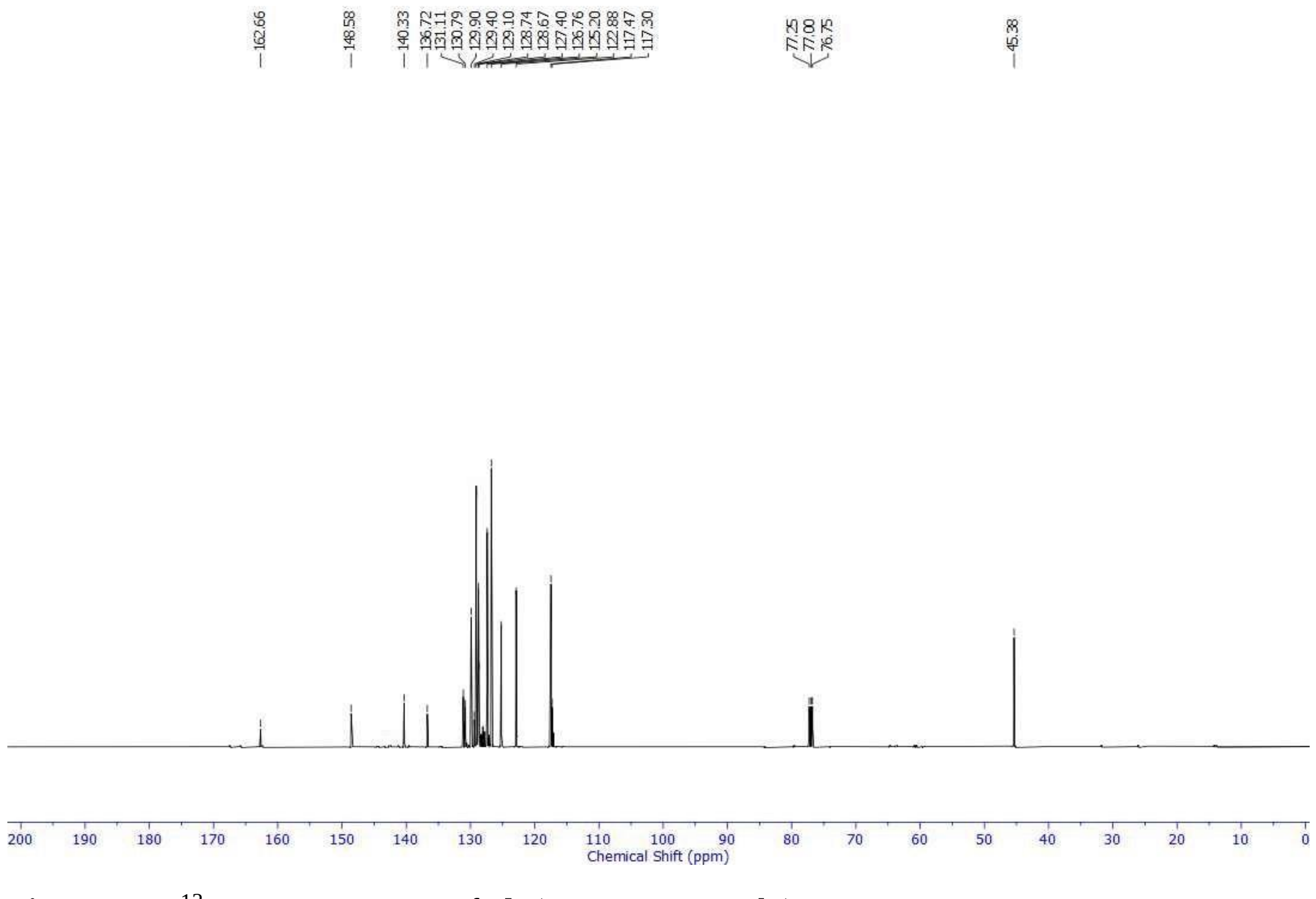

Figure S45. ^{13}C NMR spectrum of **6b** (125 MHz, CDCl3).

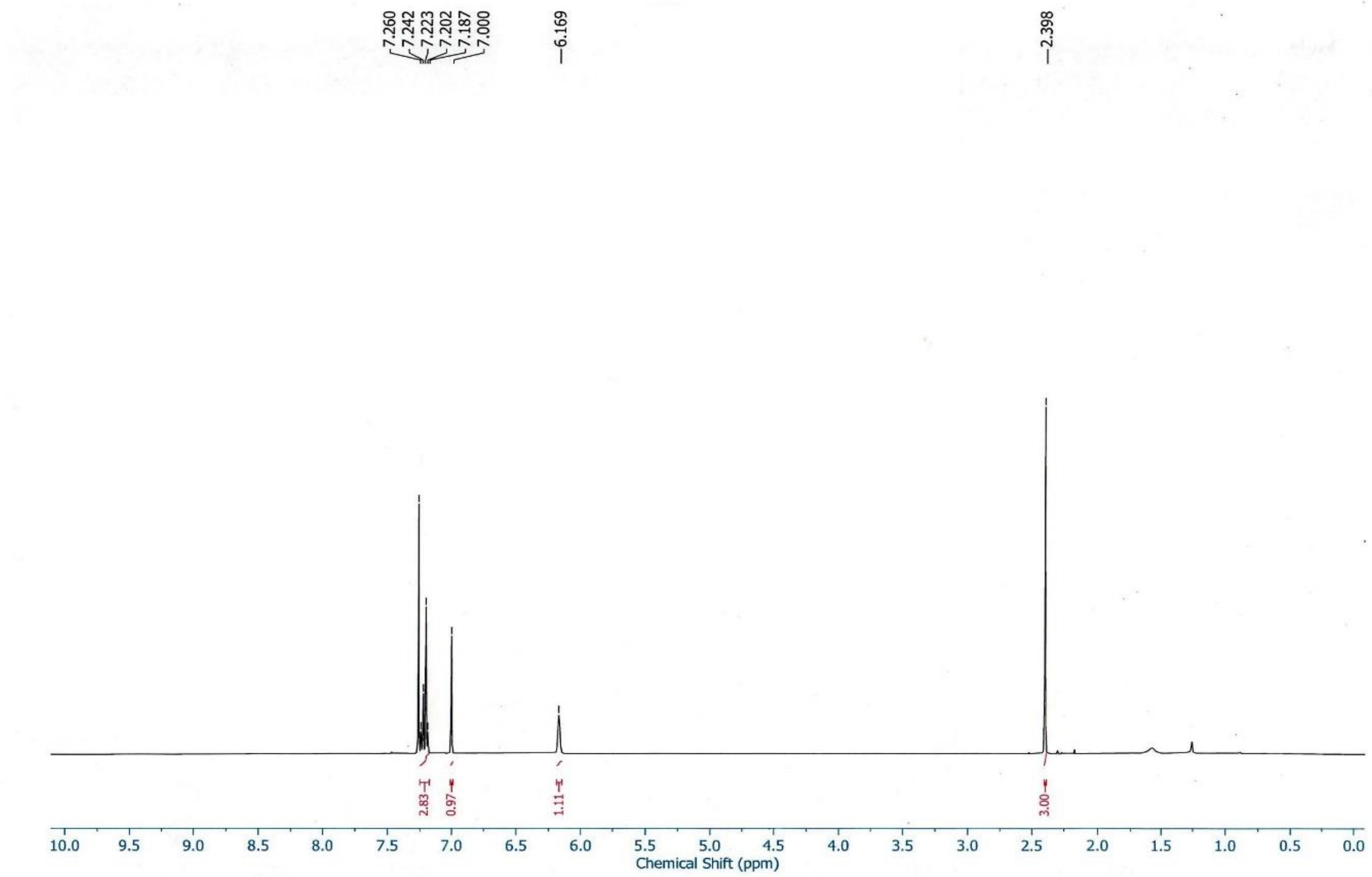

Figure S46. ^{1}H NMR spectrum of **8a** (500 MHz, $CDCl_3$).

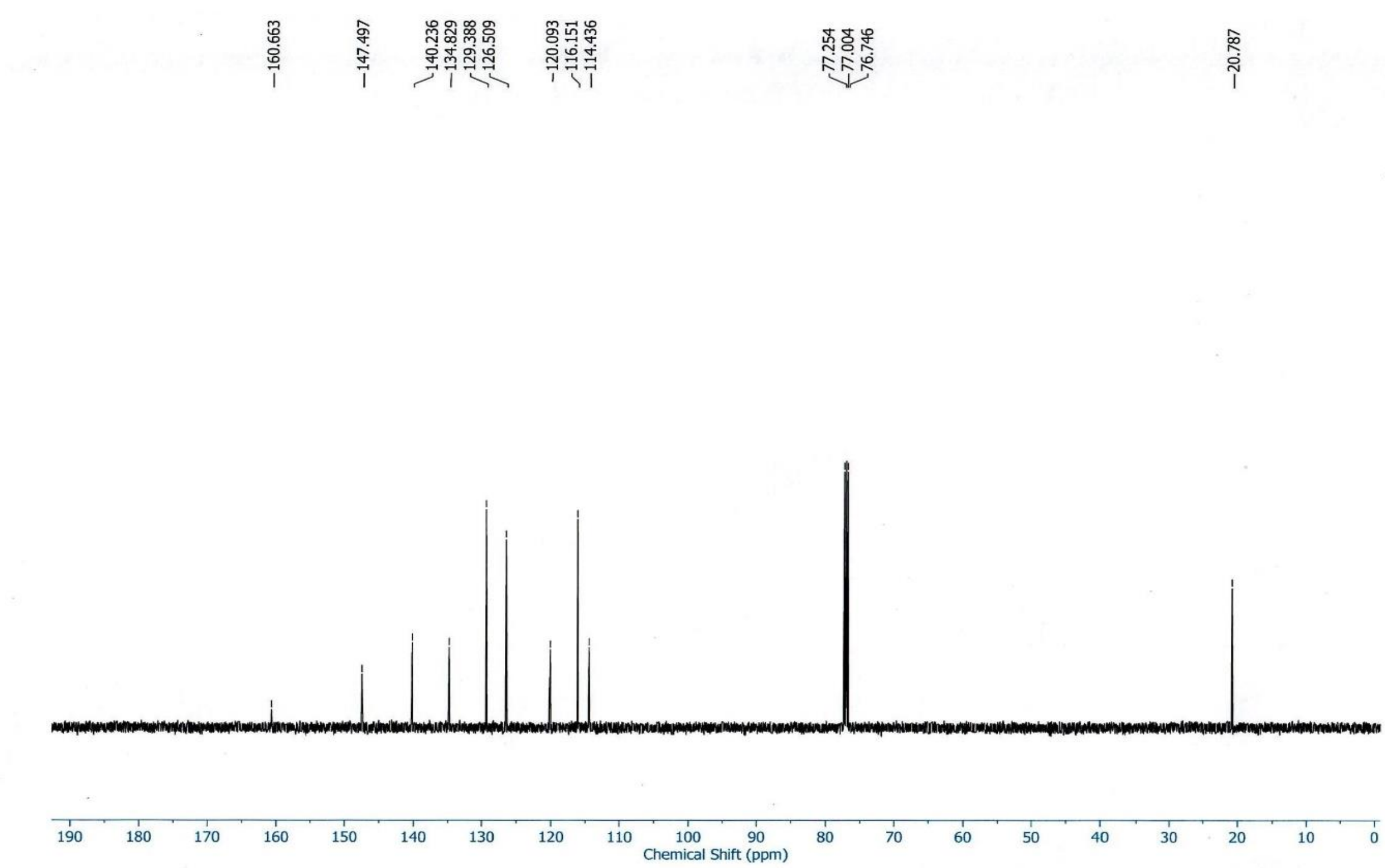

Figure S47. ^{13}C NMR spectrum of **8a** (125 MHz, $CDCl_3$).

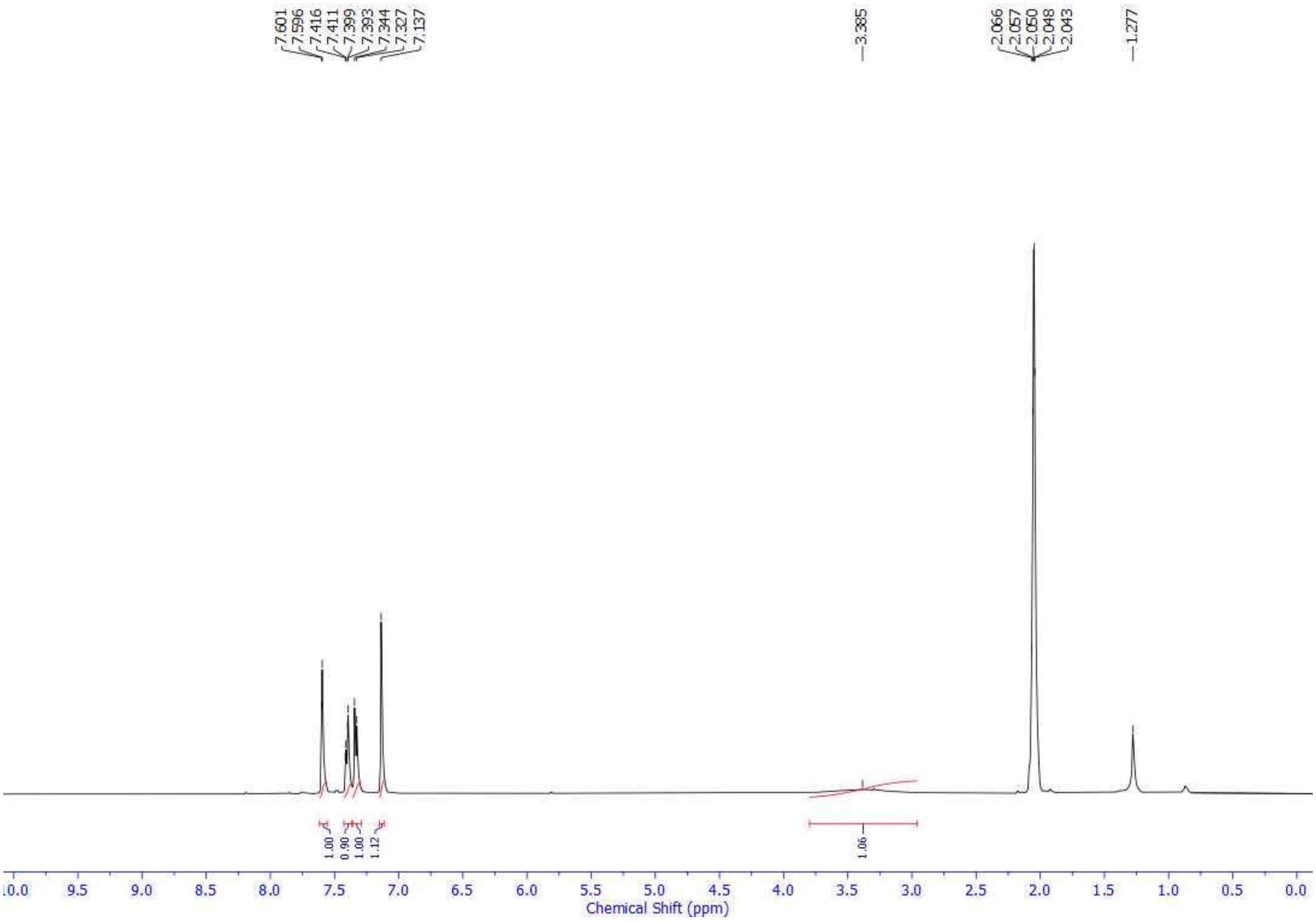

Figure S48. ^{1}H NMR spectrum of **8b** (500 MHz, $(CD_3)_2CO$).

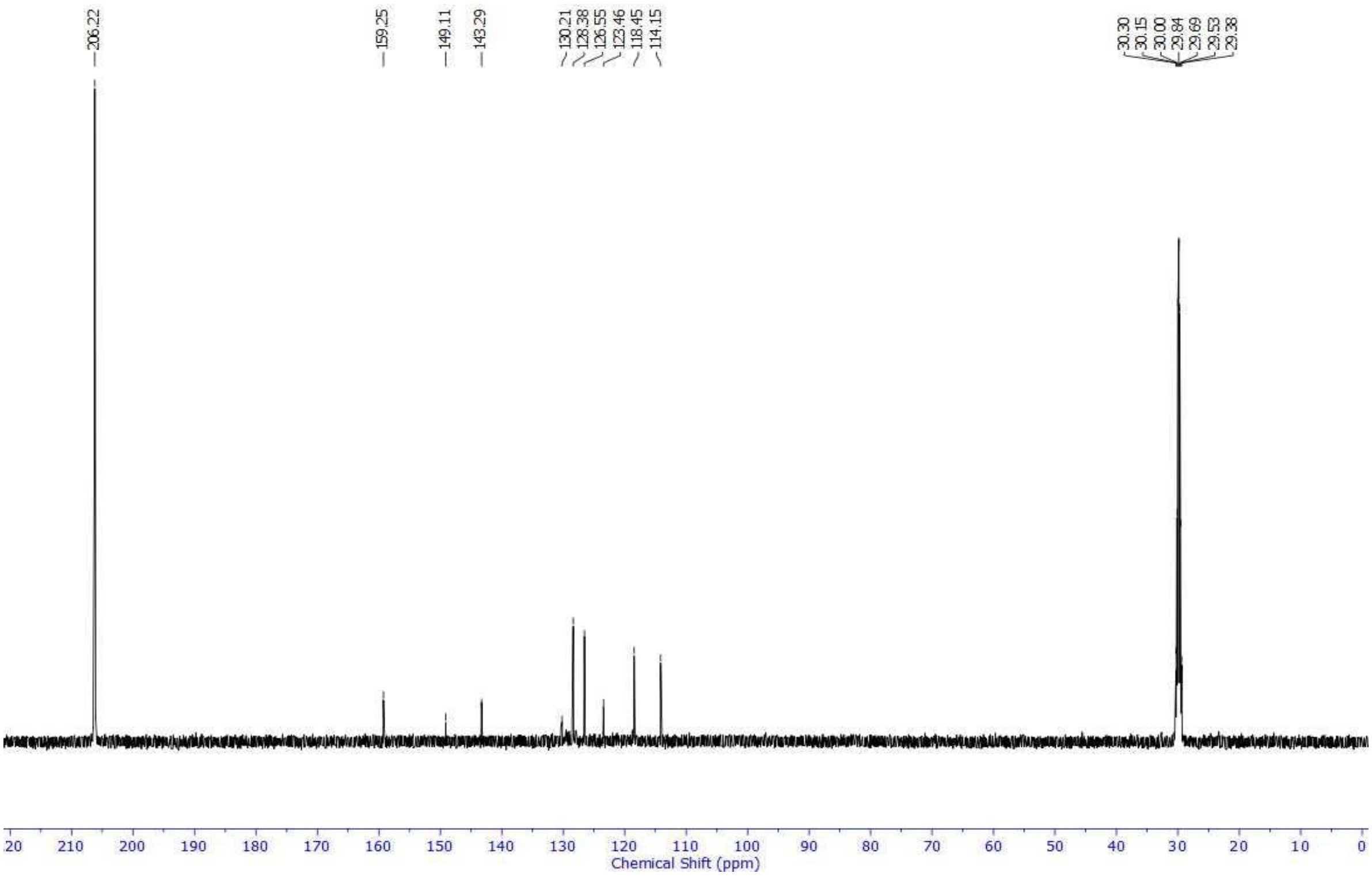

Figure S49. ^{13}C NMR spectrum of **8b** (125 MHz, $(CD_3)_2CO$).

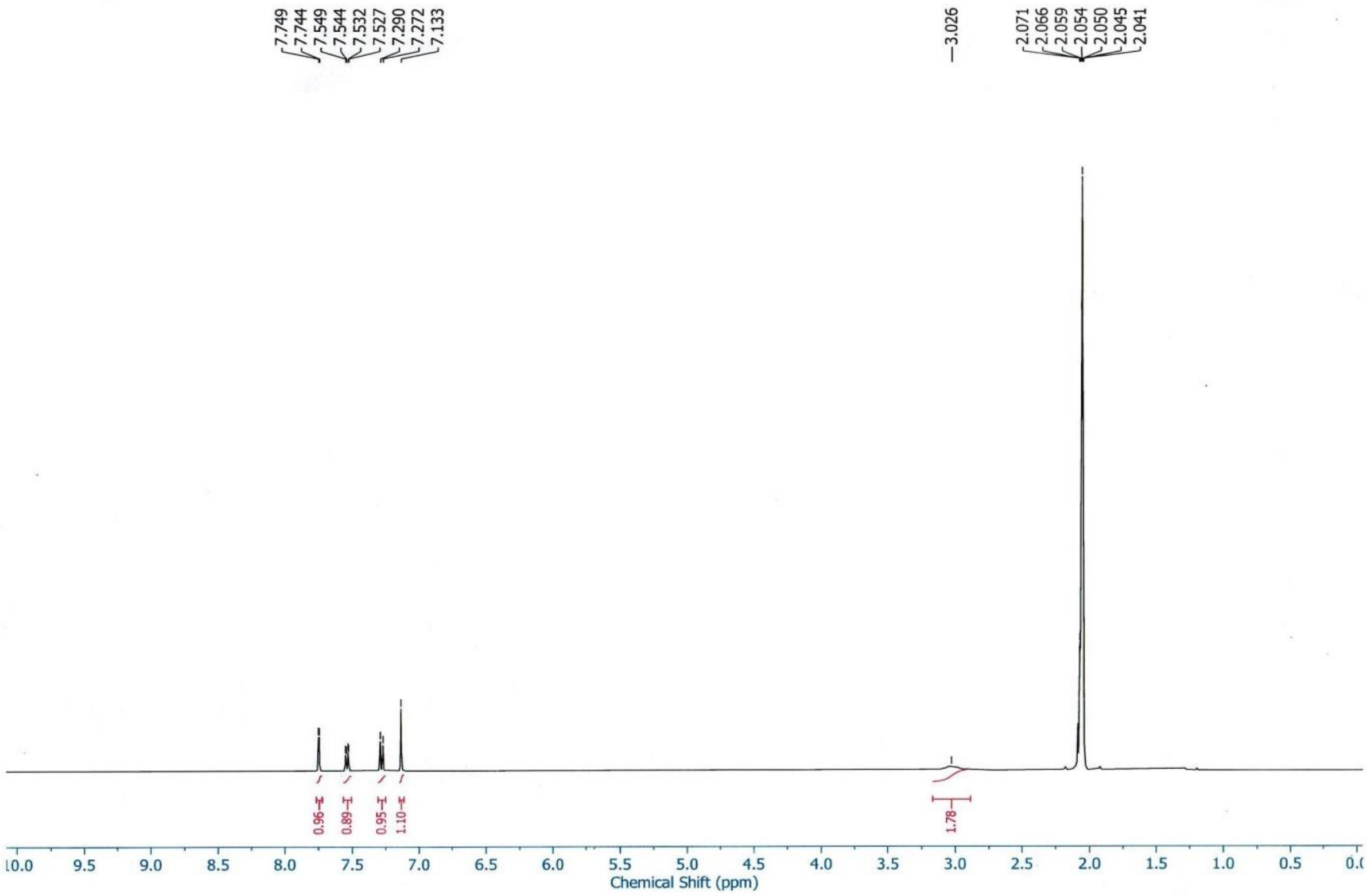

Figure S50. ^{1}H NMR spectrum of **8c** (500 MHz, $(CD_3)_2CO$).

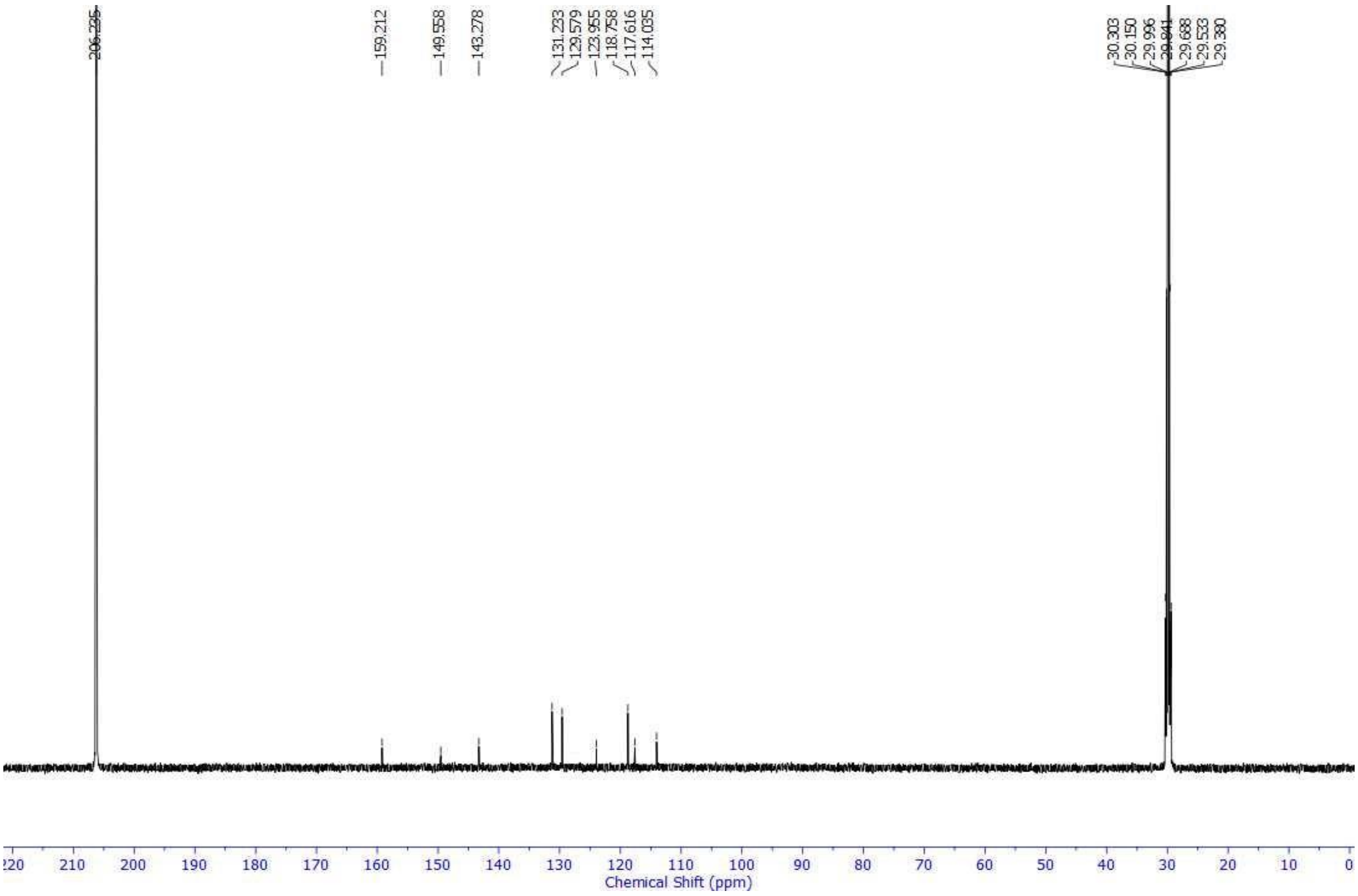

Figure S51. ^{13}C NMR spectrum of **8c** (125 MHz, $(CD_3)_2CO$).

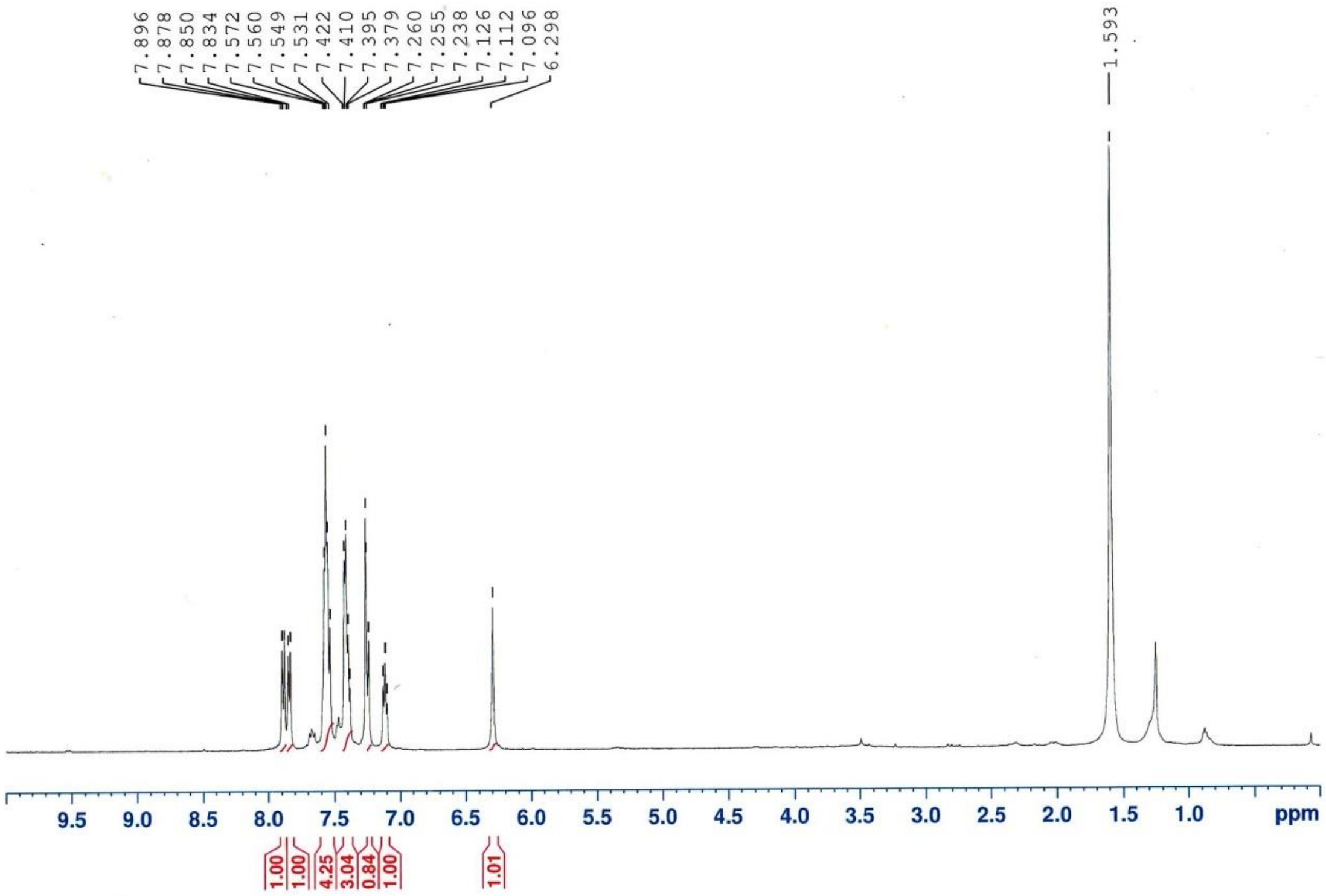

Figure S52. ^{1}H NMR spectrum of **9** (500 MHz, $CDCl_3$).

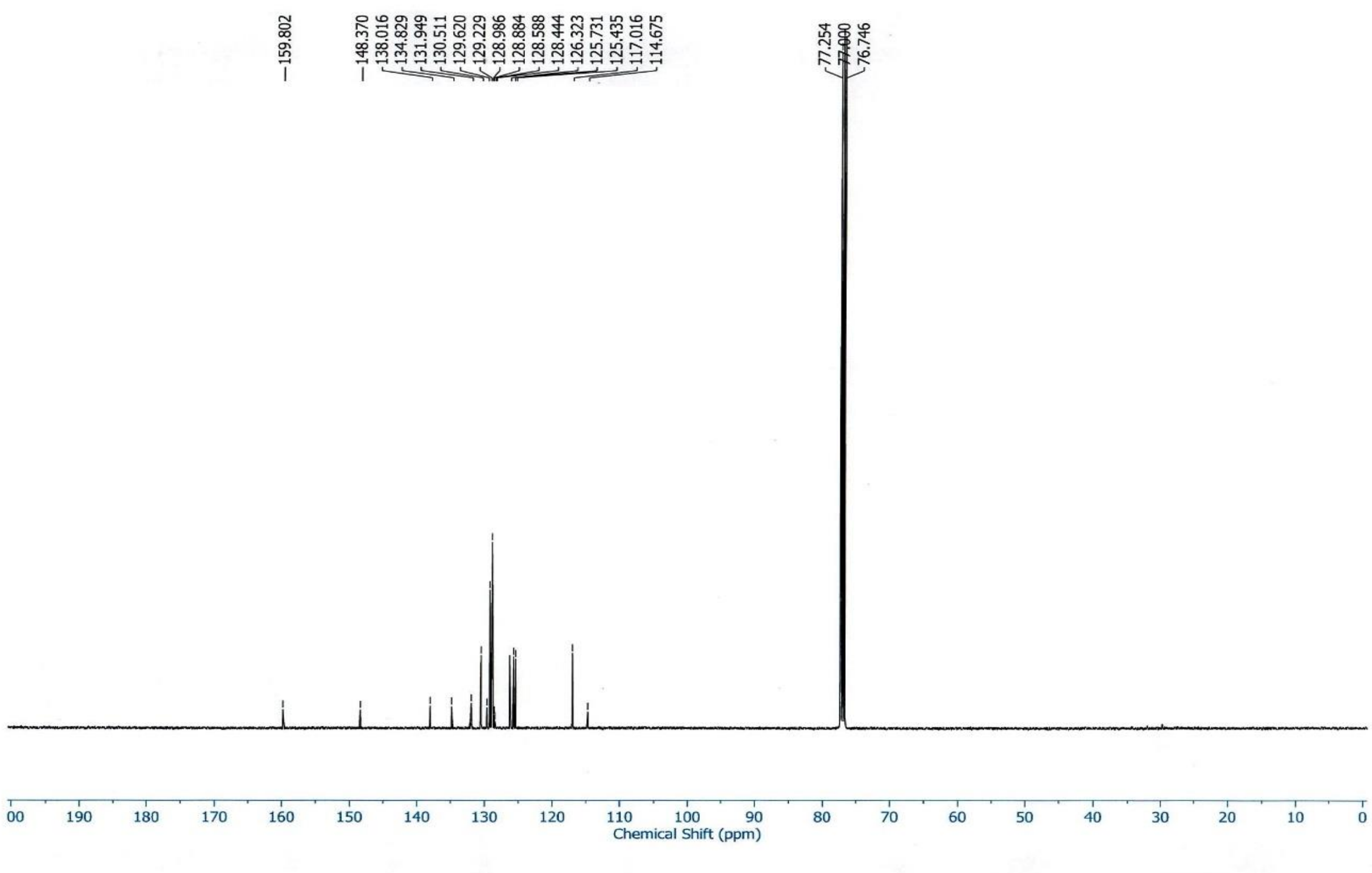

Figure S53. ^{13}C NMR spectrum of **9** (125 MHz, $CDCl_3$).

6. HRMS Spectra

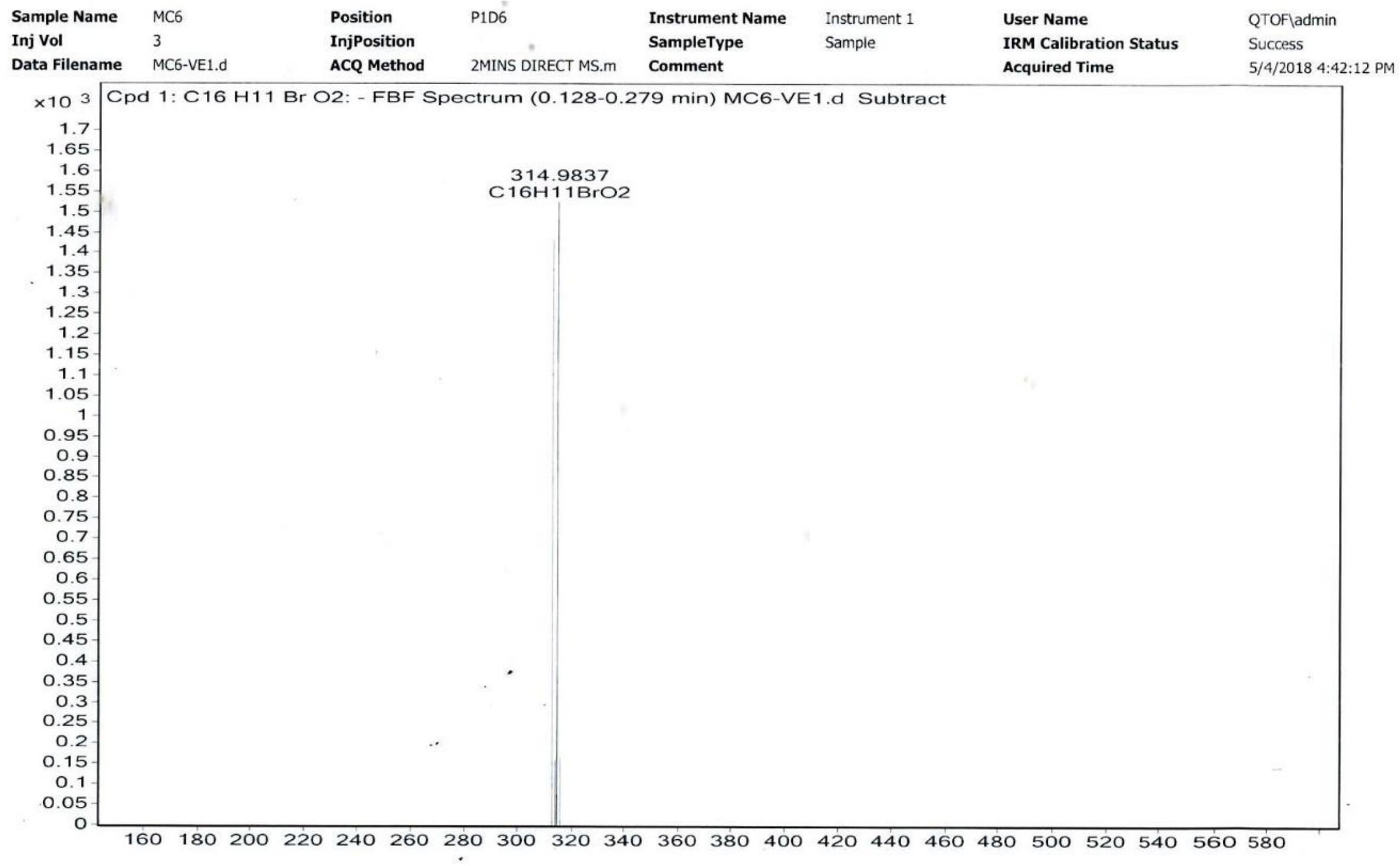

Figure S54. HRMS spectrum of **3g**.

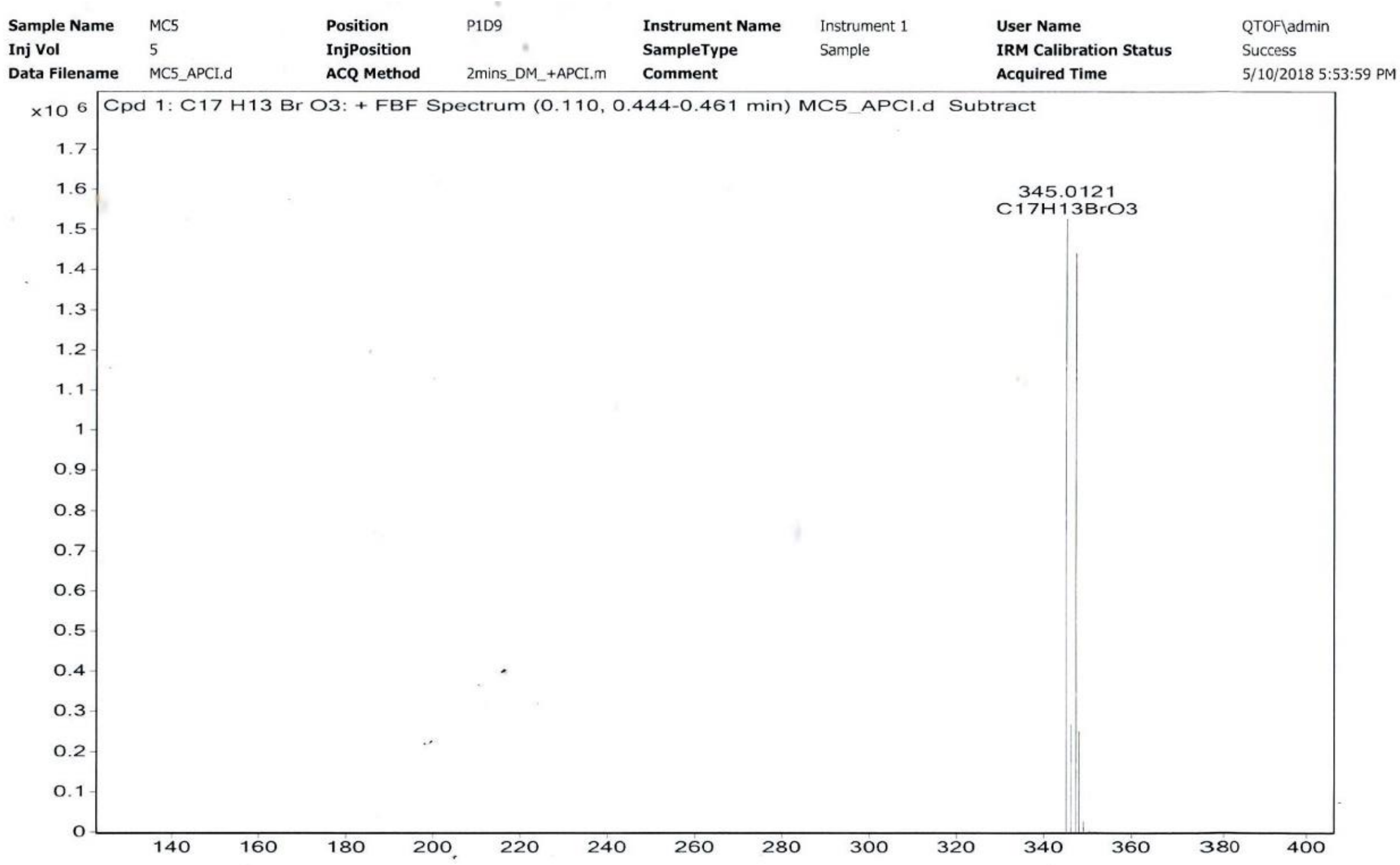

Figure S55. HRMS spectrum of **3h**.

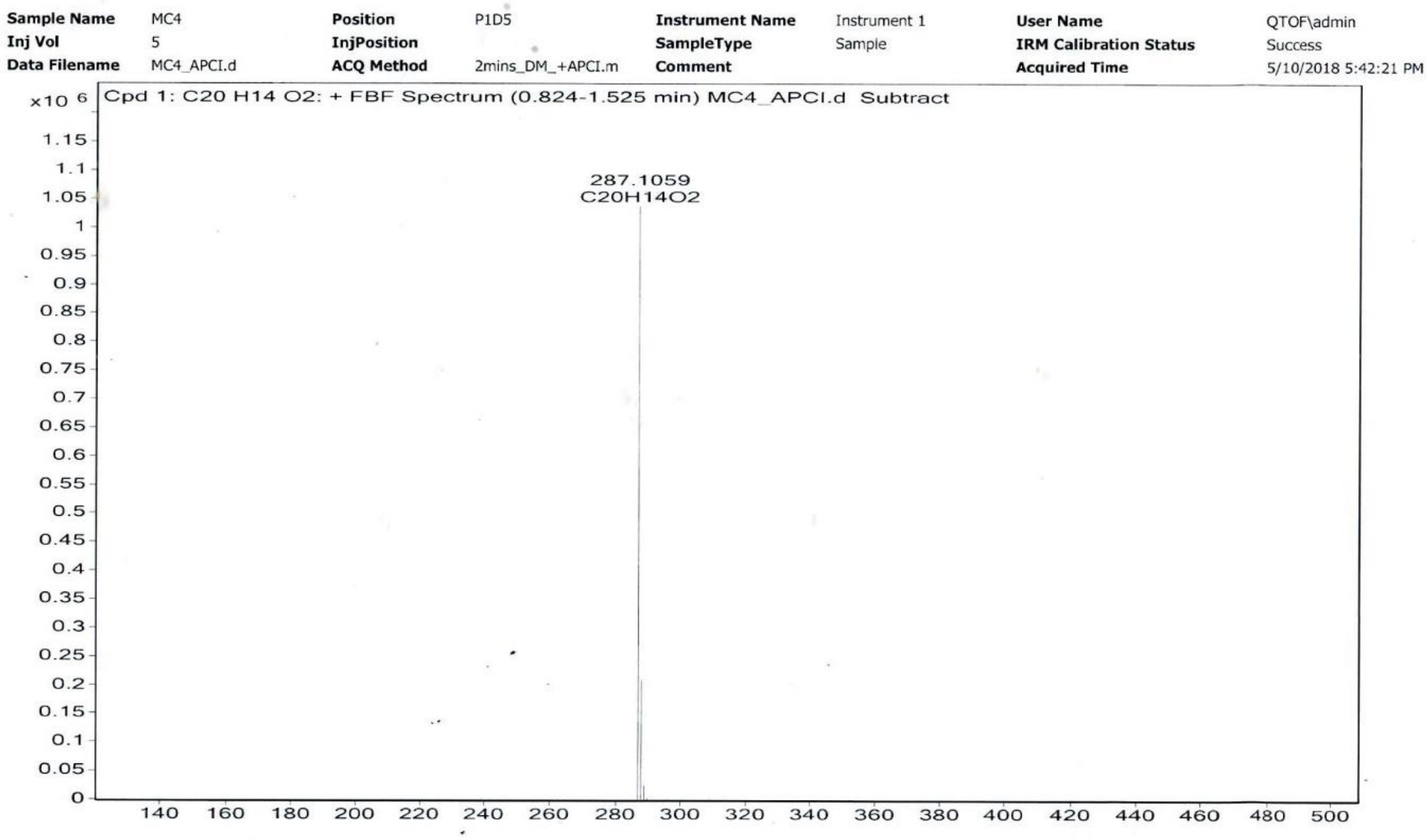

Figure S56. HRMS spectrum of **6a**.

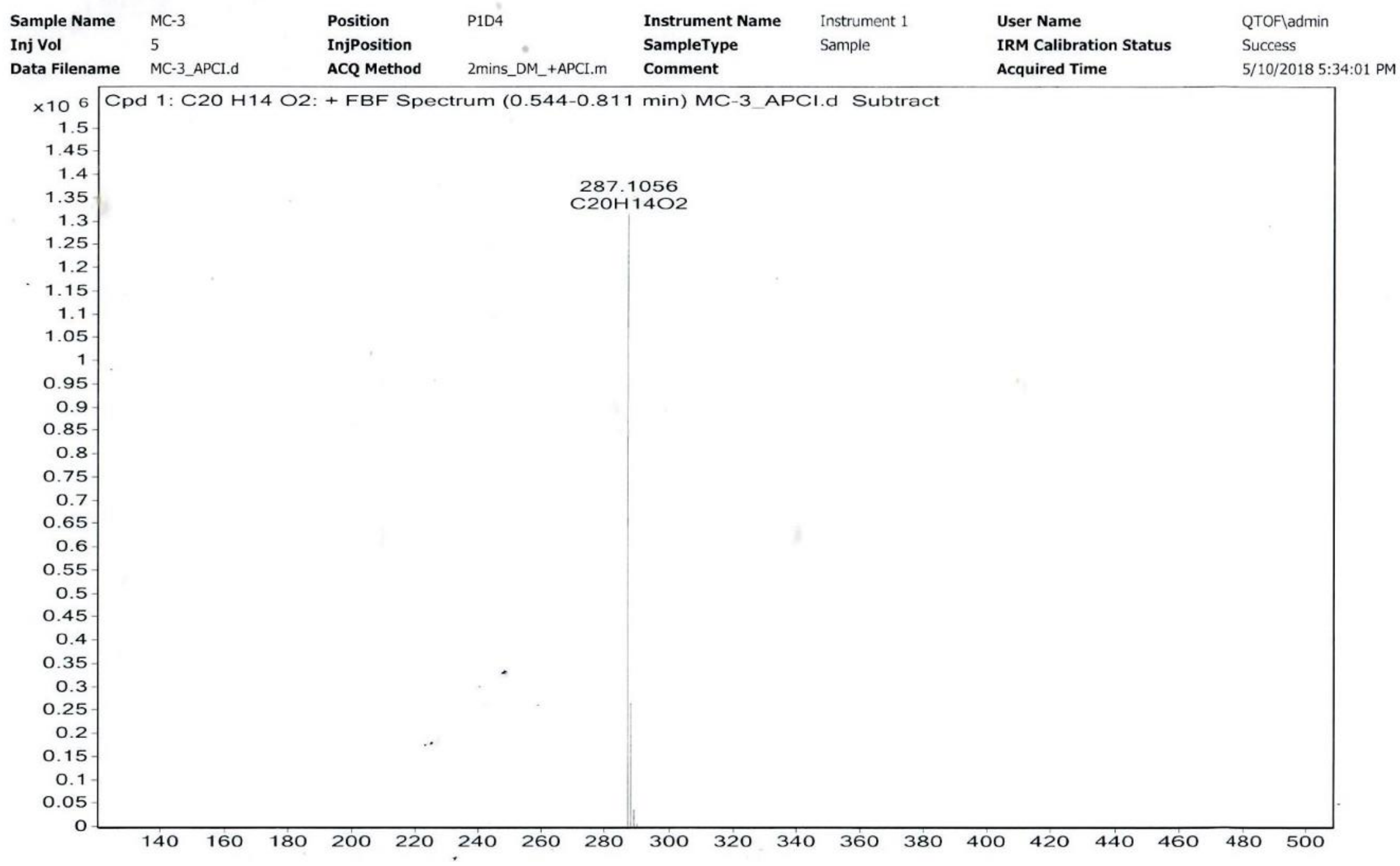

Figure S57. HRMS spectrum of **6b**.

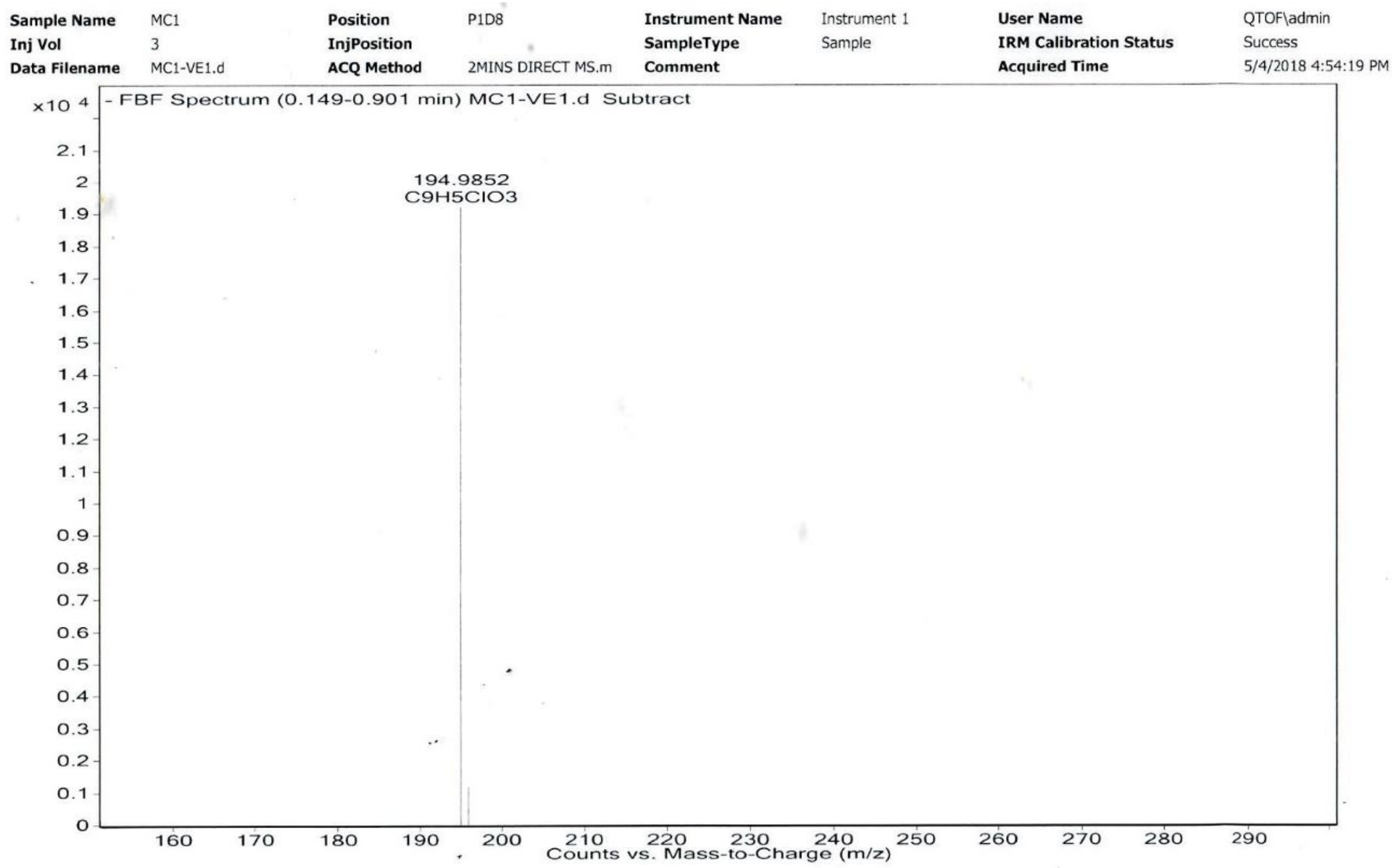

Figure S58. HRMS spectrum of **8b**.

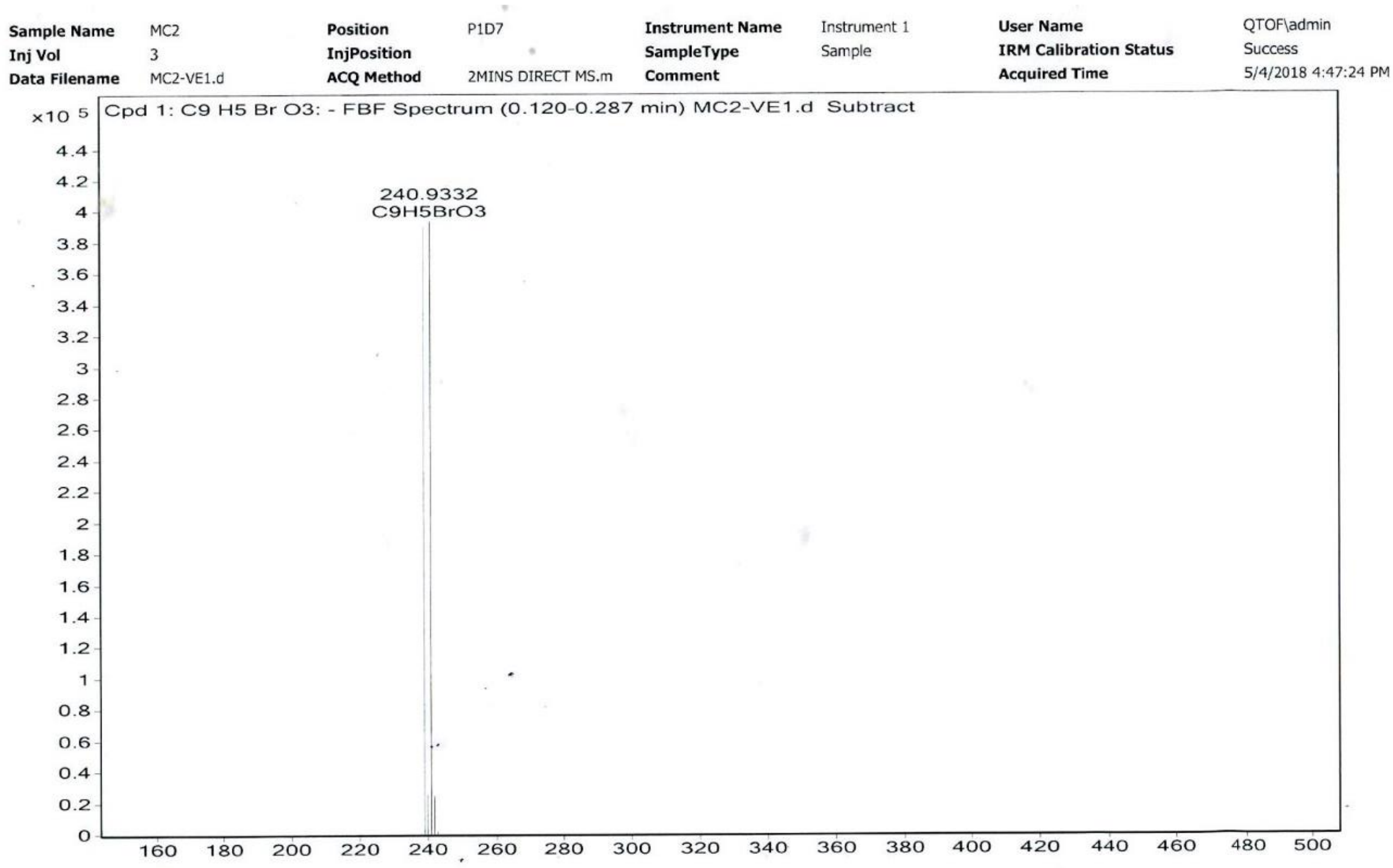

Figure S59. HRMS spectrum of **8c**.
